# Supplementary material for: How do trends in mortality inequalities by deprivation and education in Scotland and England & Wales compare? A repeat cross-sectional study
Source: BMJ Open. 2017 Jul 21;7(7):e017590. doi: 10.1136/bmjopen-2017-017590 (PMC5642664; doi:10.1136/bmjopen-2017-017590)
Supplement: Supplementary file 1 [file bmjopen-2017-017590supp001.pdf]

Supplement Table 1 - Crude and age-adjusted rates by age, sex, year, deprivation decile and country.

group	country	sex	year	decile	crude_rate	age_adj_rate	age_adj_rate_lower_CI	age_adj_rate_upper_CI	Age-adjusted mortality per 100,000 per year
E&W 1981 Women	E&W	Women	1981	1	0.009612889	0.007755622	0.007672978	0.007838266	775.5622
E&W 1981 Women	E&W	Women	1981	2	0.010476029	0.007906884	0.007825425	0.007988344	790.6884
E&W 1981 Women	E&W	Women	1981	3	0.011498301	0.008332084	0.008249331	0.008414837	833.2084
E&W 1981 Women	E&W	Women	1981	4	0.011785442	0.008367835	0.008285735	0.008449935	836.7835
E&W 1981 Women	E&W	Women	1981	5	0.012349643	0.008772567	0.008687877	0.008857258	877.2567
E&W 1981 Women	E&W	Women	1981	6	0.012604976	0.008939335	0.008853824	0.009024845	893.9335
E&W 1981 Women	E&W	Women	1981	7	0.013215572	0.009388286	0.009299824	0.009476748	938.8286
E&W 1981 Women	E&W	Women	1981	8	0.013763275	0.009750938	0.00966083	0.009841046	975.0938
E&W 1981 Women	E&W	Women	1981	9	0.014304536	0.01028942	0.010195368	0.010383472	1028.942
E&W 1981 Women	E&W	Women	1981	10	0.015758992	0.01145973	0.011357135	0.011562326	1145.973
E&W 1991 Women	E&W	Women	1991	1	0.007720282	0.006422884	0.006349762	0.006496005	642.2884
E&W 1991 Women	E&W	Women	1991	2	0.008475934	0.006741511	0.006666873	0.00681615	674.1511
E&W 1991 Women	E&W	Women	1991	3	0.009114578	0.006902071	0.006827492	0.006976651	690.2071
E&W 1991 Women	E&W	Women	1991	4	0.009720711	0.00722439	0.007147987	0.007300793	722.439
E&W 1991 Women	E&W	Women	1991	5	0.010306351	0.007550758	0.007472328	0.007629189	755.0758
E&W 1991 Women	E&W	Women	1991	6	0.010769808	0.007825069	0.007744471	0.007905667	782.5069
E&W 1991 Women	E&W	Women	1991	7	0.011524274	0.008384602	0.008300254	0.00846895	838.4602
E&W 1991 Women	E&W	Women	1991	8	0.012323026	0.008901691	0.008813651	0.00898973	890.1691
E&W 1991 Women	E&W	Women	1991	9	0.012662662	0.009270408	0.009179103	0.009361713	927.0408
E&W 1991 Women	E&W	Women	1991	10	0.013763391	0.010391625	0.010289922	0.010493329	1039.163
E&W 2001 Women	E&W	Women	2001	1	0.00579012	0.004941082	0.004879425	0.005002738	494.1082
E&W 2001 Women	E&W	Women	2001	2	0.00664026	0.005331322	0.005268285	0.005394358	533.1322
E&W 2001 Women	E&W	Women	2001	3	0.007079005	0.005542715	0.005478404	0.005607025	554.2715
E&W 2001 Women	E&W	Women	2001	4	0.007456318	0.005833322	0.005766699	0.005899944	583.3322
E&W 2001 Women	E&W	Women	2001	5	0.008136969	0.00624155	0.006172043	0.006311057	624.155
E&W 2001 Women	E&W	Women	2001	6	0.008767891	0.006741833	0.006668222	0.006815443	674.1833
E&W 2001 Women	E&W	Women	2001	7	0.009152499	0.007223277	0.007145289	0.007301265	722.3277
E&W 2001 Women	E&W	Women	2001	8	0.009599881	0.007714746	0.007632123	0.00779737	771.4746
E&W 2001 Women	E&W	Women	2001	9	0.010201663	0.008228177	0.008141838	0.008314516	822.8177

E&W 2001 Women	E&W	Women	2001	10	0.010587698	0.009121022	0.009022989	0.009219056	912.1022
E&W 2011 Women	E&W	Women	2011	1	0.00452512	0.003655192	0.003606717	0.003703668	365.5192
E&W 2011 Women	E&W	Women	2011	2	0.005046366	0.004001625	0.003950601	0.004052648	400.1625
E&W 2011 Women	E&W	Women	2011	3	0.005369199	0.004198358	0.004145849	0.004250867	419.8358
E&W 2011 Women	E&W	Women	2011	4	0.005613362	0.004515388	0.004459541	0.004571235	451.5388
E&W 2011 Women	E&W	Women	2011	5	0.006173427	0.004944128	0.004884818	0.005003437	494.4128
E&W 2011 Women	E&W	Women	2011	6	0.00644843	0.005288613	0.005225253	0.005351974	528.8613
E&W 2011 Women	E&W	Women	2011	7	0.006972373	0.005895116	0.005826416	0.005963816	589.5116
E&W 2011 Women	E&W	Women	2011	8	0.007102966	0.006229234	0.006155617	0.006302851	622.9234
E&W 2011 Women	E&W	Women	2011	9	0.00742091	0.006763139	0.006682834	0.006843444	676.3139
E&W 2011 Women	E&W	Women	2011	10	0.007504293	0.007255963	0.007166445	0.007345481	725.5963
E&W 1981 Men	E&W	Men	1981	1	0.013836215	0.013282844	0.01316358	0.013402108	1328.284
E&W 1981 Men	E&W	Men	1981	2	0.015771348	0.014010972	0.013892168	0.014129777	1401.097
E&W 1981 Men	E&W	Men	1981	3	0.01732869	0.014876871	0.014755229	0.014998513	1487.687
E&W 1981 Men	E&W	Men	1981	4	0.017783471	0.014983149	0.014861826	0.015104471	1498.315
E&W 1981 Men	E&W	Men	1981	5	0.018750443	0.01581156	0.015685979	0.015937141	1581.156
E&W 1981 Men	E&W	Men	1981	6	0.019528808	0.016484809	0.016356086	0.016613532	1648.481
E&W 1981 Men	E&W	Men	1981	7	0.020527071	0.017374747	0.017241317	0.017508177	1737.475
E&W 1981 Men	E&W	Men	1981	8	0.021516646	0.018263609	0.018126787	0.018400431	1826.361
E&W 1981 Men	E&W	Men	1981	9	0.022342745	0.019196938	0.019054611	0.019339266	1919.694
E&W 1981 Men	E&W	Men	1981	10	0.024349277	0.021351602	0.021197785	0.02150542	2135.16
E&W 1991 Men	E&W	Men	1991	1	0.01103878	0.010488629	0.010388499	0.010588758	1048.863
E&W 1991 Men	E&W	Men	1991	2	0.01226422	0.011254249	0.011150527	0.011357971	1125.425
E&W 1991 Men	E&W	Men	1991	3	0.01325232	0.01165966	0.011554955	0.011764365	1165.966
E&W 1991 Men	E&W	Men	1991	4	0.014156857	0.012279371	0.012171604	0.012387137	1227.937
E&W 1991 Men	E&W	Men	1991	5	0.014950535	0.012817366	0.012706626	0.012928105	1281.737
E&W 1991 Men	E&W	Men	1991	6	0.015800782	0.013509515	0.013394194	0.013624835	1350.951
E&W 1991 Men	E&W	Men	1991	7	0.017159268	0.014640691	0.014519764	0.014761619	1464.069
E&W 1991 Men	E&W	Men	1991	8	0.018230566	0.015567663	0.015441112	0.015694214	1556.766
E&W 1991 Men	E&W	Men	1991	9	0.019024402	0.016314241	0.016183029	0.016445452	1631.424
E&W 1991 Men	E&W	Men	1991	10	0.021058194	0.018420098	0.018274859	0.018565338	1842.01
E&W 2001 Men	E&W	Men	2001	1	0.008202026	0.007523487	0.007444123	0.007602851	752.3487

E&W 2001 Men	E&W	Men	2001	2	0.009261247	0.008104411	0.00802296	0.008185863	810.4411
E&W 2001 Men	E&W	Men	2001	3	0.010043937	0.008723544	0.008638119	0.008808969	872.3544
E&W 2001 Men	E&W	Men	2001	4	0.010621459	0.009262298	0.009173214	0.009351383	926.2298
E&W 2001 Men	E&W	Men	2001	5	0.011491073	0.009944548	0.009851023	0.010038073	994.4548
E&W 2001 Men	E&W	Men	2001	6	0.01228122	0.010788395	0.010688931	0.010887858	1078.839
E&W 2001 Men	E&W	Men	2001	7	0.01297947	0.011653791	0.011547806	0.011759775	1165.379
E&W 2001 Men	E&W	Men	2001	8	0.013801846	0.01256548	0.012453197	0.012677762	1256.548
E&W 2001 Men	E&W	Men	2001	9	0.015039413	0.013770089	0.013651084	0.013889093	1377.009
E&W 2001 Men	E&W	Men	2001	10	0.016328106	0.015628966	0.015493243	0.01576469	1562.897
E&W 2011 Men	E&W	Men	2011	1	0.006531269	0.005324398	0.005264408	0.005384389	532.4398
E&W 2011 Men	E&W	Men	2011	2	0.007209251	0.005844454	0.005780896	0.005908012	584.4454
E&W 2011 Men	E&W	Men	2011	3	0.00771371	0.006289675	0.006222895	0.006356455	628.9675
E&W 2011 Men	E&W	Men	2011	4	0.008046375	0.006782732	0.006711645	0.00685382	678.2732
E&W 2011 Men	E&W	Men	2011	5	0.008744033	0.007441684	0.00736579	0.007517578	744.1684
E&W 2011 Men	E&W	Men	2011	6	0.009159381	0.008064742	0.007983004	0.00814648	806.4742
E&W 2011 Men	E&W	Men	2011	7	0.009774983	0.008875782	0.008787907	0.008963658	887.5782
E&W 2011 Men	E&W	Men	2011	8	0.010086383	0.009604045	0.009508757	0.009699333	960.4045
E&W 2011 Men	E&W	Men	2011	9	0.010646058	0.010524807	0.010420662	0.010628953	1052.481
E&W 2011 Men	E&W	Men	2011	10	0.011066971	0.011640176	0.011522596	0.011757755	1164.018
Scotland 1981 Women	Scotland	Women	1981	1	0.010525108	0.008241884	0.007898118	0.008585651	824.1884
Scotland 1981 Women	Scotland	Women	1981	2	0.013282962	0.00911192	0.008803342	0.009420497	911.192
Scotland 1981 Women	Scotland	Women	1981	3	0.013353083	0.009459393	0.009169682	0.009749104	945.9393
Scotland 1981 Women	Scotland	Women	1981	4	0.014387775	0.01028801	0.009965086	0.010610935	1028.801
Scotland 1981 Women	Scotland	Women	1981	5	0.014238784	0.010217297	0.009906958	0.010527636	1021.73
Scotland 1981 Women	Scotland	Women	1981	6	0.014408361	0.01067589	0.010387509	0.010964272	1067.589
Scotland 1981 Women	Scotland	Women	1981	7	0.014307882	0.011156961	0.010869841	0.011444081	1115.696
Scotland 1981 Women	Scotland	Women	1981	8	0.015429545	0.011470359	0.011197754	0.011742964	1147.036
Scotland 1981 Women	Scotland	Women	1981	9	0.015946955	0.012255514	0.011984849	0.012526179	1225.551
Scotland 1981 Women	Scotland	Women	1981	10	0.017832188	0.01393502	0.013631905	0.014238135	1393.502
Scotland 1991 Women	Scotland	Women	1991	1	0.007778107	0.006961045	0.006652506	0.007269583	696.1045
Scotland 1991 Women	Scotland	Women	1991	2	0.010644362	0.008145254	0.00784715	0.008443357	814.5254
Scotland 1991 Women	Scotland	Women	1991	3	0.010573639	0.008200427	0.007914969	0.008485886	820.0427

Scotland 1991 Women	Scotland	Women	1991	4	0.011319426	0.008554462	0.008286397	0.008822528	855.4462
Scotland 1991 Women	Scotland	Women	1991	5	0.011419762	0.008703245	0.008439098	0.008967393	870.3245
Scotland 1991 Women	Scotland	Women	1991	6	0.011959812	0.00912001	0.008847431	0.009392588	912.001
Scotland 1991 Women	Scotland	Women	1991	7	0.01254952	0.009723669	0.009461309	0.009986029	972.3669
Scotland 1991 Women	Scotland	Women	1991	8	0.014283347	0.010841657	0.010559872	0.011123442	1084.166
Scotland 1991 Women	Scotland	Women	1991	9	0.014621061	0.010985495	0.010716051	0.011254939	1098.55
Scotland 1991 Women	Scotland	Women	1991	10	0.017393045	0.013147151	0.012832815	0.013461487	1314.715
Scotland 2001 Women	Scotland	Women	2001	1	0.006054715	0.005369293	0.005119773	0.005618813	536.9293
Scotland 2001 Women	Scotland	Women	2001	2	0.007473842	0.006167007	0.005932055	0.006401959	616.7007
Scotland 2001 Women	Scotland	Women	2001	3	0.007940882	0.006723365	0.006462115	0.006984616	672.3365
Scotland 2001 Women	Scotland	Women	2001	4	0.008454126	0.006752374	0.00650612	0.006998627	675.2374
Scotland 2001 Women	Scotland	Women	2001	5	0.008833125	0.006991448	0.006751617	0.007231278	699.1448
Scotland 2001 Women	Scotland	Women	2001	6	0.009997091	0.007983593	0.007739673	0.008227513	798.3593
Scotland 2001 Women	Scotland	Women	2001	7	0.01012218	0.008270903	0.008026704	0.008515101	827.0903
Scotland 2001 Women	Scotland	Women	2001	8	0.011031647	0.008757081	0.00851662	0.008997541	875.7081
Scotland 2001 Women	Scotland	Women	2001	9	0.012063884	0.009551886	0.009302581	0.00980119	955.1886
Scotland 2001 Women	Scotland	Women	2001	10	0.014525514	0.011720127	0.011420843	0.01201941	1172.013
Scotland 2011 Women	Scotland	Women	2011	1	0.005188653	0.004390383	0.00421379	0.004566976	439.0383
Scotland 2011 Women	Scotland	Women	2011	2	0.005999477	0.004874167	0.004690536	0.005057797	487.4167
Scotland 2011 Women	Scotland	Women	2011	3	0.006649881	0.005285443	0.005095168	0.005475718	528.5443
Scotland 2011 Women	Scotland	Women	2011	4	0.007424119	0.005853295	0.005651062	0.006055528	585.3295
Scotland 2011 Women	Scotland	Women	2011	5	0.007461833	0.006041823	0.005833749	0.006249897	604.1823
Scotland 2011 Women	Scotland	Women	2011	6	0.00826023	0.006869481	0.006642285	0.007096677	686.9481
Scotland 2011 Women	Scotland	Women	2011	7	0.008382318	0.006941867	0.006709153	0.007174581	694.1867
Scotland 2011 Women	Scotland	Women	2011	8	0.00930957	0.007758332	0.00750849	0.008008174	775.8332
Scotland 2011 Women	Scotland	Women	2011	9	0.010235269	0.008278501	0.008024174	0.008532828	827.8501
Scotland 2011 Women	Scotland	Women	2011	10	0.01181909	0.010062415	0.009766534	0.010358295	1006.241
Scotland 1981 Men	Scotland	Men	1981	1	0.014829473	0.014645599	0.014114115	0.015177084	1464.56
Scotland 1981 Men	Scotland	Men	1981	2	0.018356204	0.01548973	0.015027761	0.0159517	1548.973
Scotland 1981 Men	Scotland	Men	1981	3	0.019749633	0.017033235	0.016591014	0.017475456	1703.323
Scotland 1981 Men	Scotland	Men	1981	4	0.020395399	0.0174934	0.017016793	0.017970006	1749.34
Scotland 1981 Men	Scotland	Men	1981	5	0.020899901	0.018031108	0.017562668	0.018499549	1803.111

Scotland 1981 Men	Scotland	Men	1981	6	0.021253547	0.018943333	0.018504819	0.019381847	1894.333
Scotland 1981 Men	Scotland	Men	1981	7	0.021440803	0.019655468	0.019223348	0.020087587	1965.547
Scotland 1981 Men	Scotland	Men	1981	8	0.023382957	0.020479833	0.020064179	0.020895487	2047.983
Scotland 1981 Men	Scotland	Men	1981	9	0.023369206	0.021274203	0.020864997	0.021683409	2127.42
Scotland 1981 Men	Scotland	Men	1981	10	0.026627273	0.02451257	0.024055239	0.0249699	2451.257
Scotland 1991 Men	Scotland	Men	1991	1	0.010887721	0.011516785	0.011076989	0.011956582	1151.679
Scotland 1991 Men	Scotland	Men	1991	2	0.01369037	0.012734436	0.012317722	0.01315115	1273.444
Scotland 1991 Men	Scotland	Men	1991	3	0.014336067	0.013406395	0.013000268	0.013812523	1340.64
Scotland 1991 Men	Scotland	Men	1991	4	0.015868404	0.014157409	0.013775834	0.014538983	1415.741
Scotland 1991 Men	Scotland	Men	1991	5	0.016307012	0.014769597	0.014385004	0.01515419	1476.96
Scotland 1991 Men	Scotland	Men	1991	6	0.016837016	0.015184471	0.014790523	0.015578419	1518.447
Scotland 1991 Men	Scotland	Men	1991	7	0.018238995	0.016798337	0.016411561	0.017185113	1679.834
Scotland 1991 Men	Scotland	Men	1991	8	0.019793804	0.01748933	0.017087907	0.017890753	1748.933
Scotland 1991 Men	Scotland	Men	1991	9	0.021303564	0.018721615	0.018327746	0.019115484	1872.161
Scotland 1991 Men	Scotland	Men	1991	10	0.025642796	0.022471745	0.02201256	0.02293093	2247.174
Scotland 2001 Men	Scotland	Men	2001	1	0.008092963	0.007992258	0.007663932	0.008320584	799.2258
Scotland 2001 Men	Scotland	Men	2001	2	0.009987633	0.009536619	0.009219267	0.009853972	953.6619
Scotland 2001 Men	Scotland	Men	2001	3	0.011075126	0.010522013	0.010168719	0.010875308	1052.201
Scotland 2001 Men	Scotland	Men	2001	4	0.011944081	0.01074405	0.010409199	0.011078901	1074.405
Scotland 2001 Men	Scotland	Men	2001	5	0.012804428	0.011731175	0.011391259	0.012071091	1173.117
Scotland 2001 Men	Scotland	Men	2001	6	0.01411566	0.012887867	0.012549888	0.013225846	1288.787
Scotland 2001 Men	Scotland	Men	2001	7	0.01443252	0.013449304	0.013108893	0.013789715	1344.93
Scotland 2001 Men	Scotland	Men	2001	8	0.016276902	0.015007962	0.014660355	0.015355569	1500.796
Scotland 2001 Men	Scotland	Men	2001	9	0.017687748	0.016079247	0.015721943	0.01643655	1607.925
Scotland 2001 Men	Scotland	Men	2001	10	0.022427071	0.020676718	0.02023799	0.021115446	2067.672
Scotland 2011 Men	Scotland	Men	2011	1	0.006641684	0.005838869	0.00562563	0.006052108	583.8869
Scotland 2011 Men	Scotland	Men	2011	2	0.008213557	0.006959545	0.00673004	0.007189049	695.9545
Scotland 2011 Men	Scotland	Men	2011	3	0.009326207	0.007787182	0.007543687	0.008030678	778.7182
Scotland 2011 Men	Scotland	Men	2011	4	0.009918607	0.008362189	0.008105543	0.008618836	836.2189
Scotland 2011 Men	Scotland	Men	2011	5	0.010430598	0.009189839	0.008916447	0.00946323	918.9839
Scotland 2011 Men	Scotland	Men	2011	6	0.01124958	0.010073004	0.009780454	0.010365554	1007.3
Scotland 2011 Men	Scotland	Men	2011	7	0.011551229	0.010486904	0.010182382	0.010791425	1048.69

Scotland 2011 Men	Scotland	Men	2011	8	0.012963478	0.011963323	0.011630806	0.012295839	1196.332
Scotland 2011 Men	Scotland	Men	2011	9	0.014115218	0.01268553	0.01234699	0.01302407	1268.553
Scotland 2011 Men	Scotland	Men	2011	10	0.017255414	0.01641974	0.016013286	0.016826194	1641.974

Supplement Figure 1 - Age-adjusted mortality rates per 100,000 per year by deprivation tenth, women, England & Wales

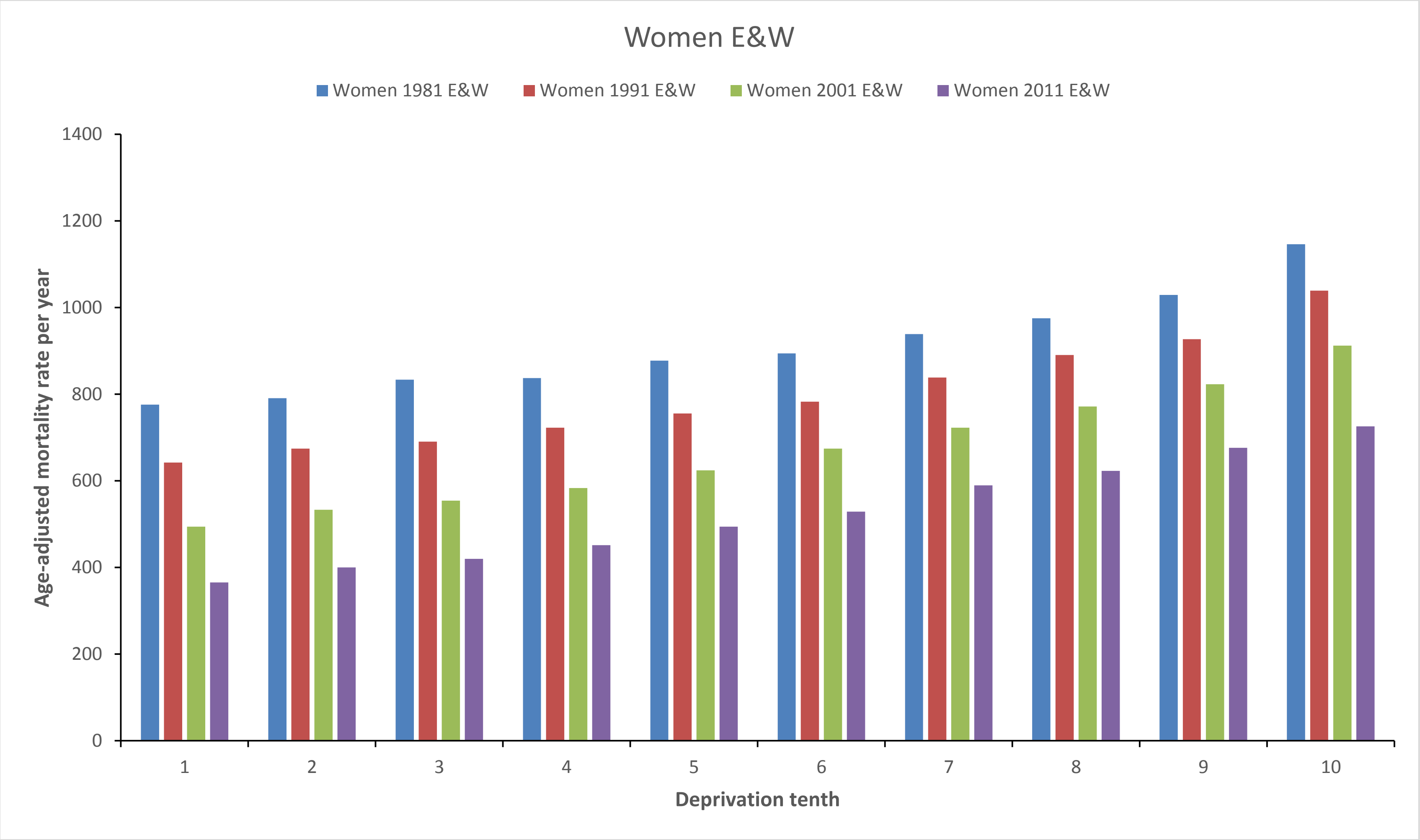

Supplement Figure 2 - Age-adjusted mortality rates per 100,000 per year by deprivation tenth, men, England & Wales

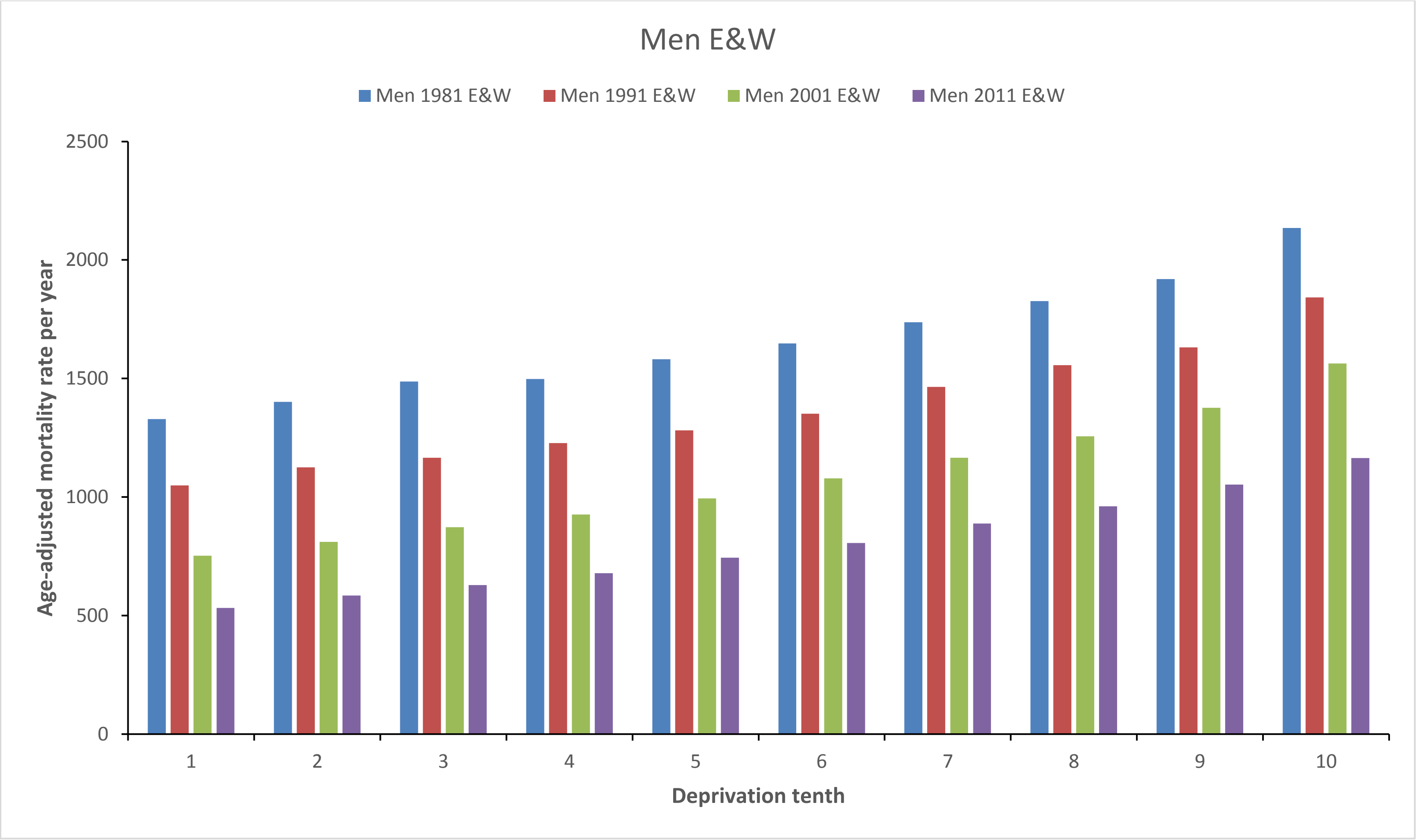

Supplement Figure 3 - Age-adjusted mortality rates per 100,000 per year by deprivation tenth, women, Scotland

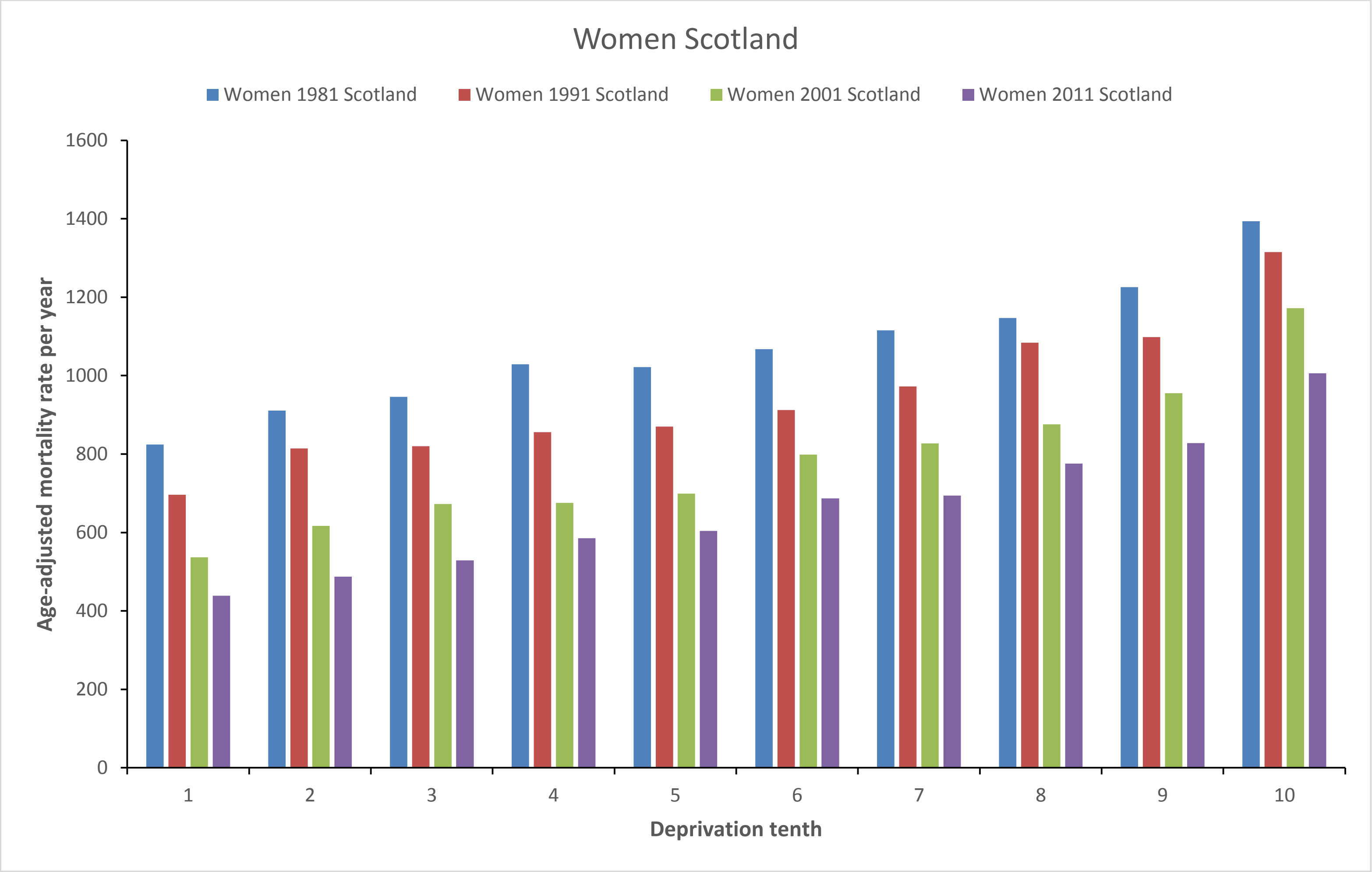

Supplement Figure 4 - Age-adjusted mortality rates per 100,000 per year by deprivation tenth, men, Scotland

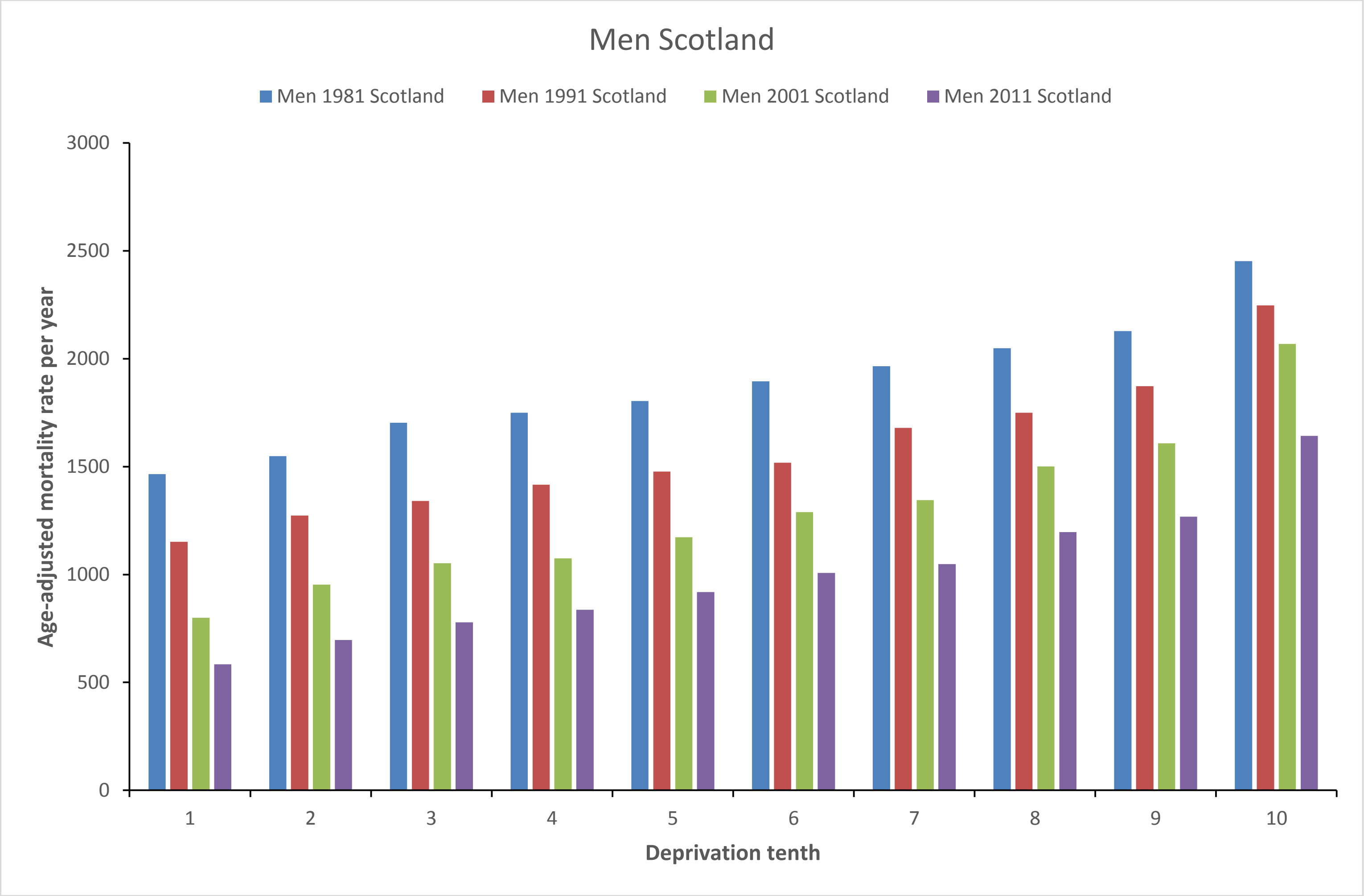

Supplement Table 2 - Relative Index of Inequality and Slope Index of Inequality for mortality by year, sex and country

group	country	year	sex	SII	SII -lower ci	SI -upper CI	RII	RII -lower	RII _upper	SII per 100,000 per year		
E&W 1981 Men	E&W	1981	Men	0.007681	0.007525	0.007837645	1.6054	1.5913	1.619499	768.1188	752.5133	783.7645453
E&W 1991 Men	E&W	1991	Men	0.007477	0.007338	0.007616669	1.7599	1.7433	1.776685	747.7353	733.837	761.6668559
E&W 2001 Men	E&W	2001	Men	0.007581	0.007463	0.007699371	2.1199	2.098	2.142026	758.1331	746.35	769.9370795
E&W 2011 Men	E&W	2011	Men	0.006076	0.005981	0.006170969	2.2948	2.2686	2.321268	607.6128	598.1439	617.0969031
Scotland 1981 Men	Scotland	1981	Men	0.008873	0.008331	0.009418183	1.5937	1.5528	1.635678	887.267	833.1399	941.8182932
Scotland 1991 Men	Scotland	1991	Men	0.009623	0.00913	0.01011968	1.8457	1.7942	1.898683	962.3261	913.0035	1011.967998
Scotland 2001 Men	Scotland	2001	Men	0.010619	0.010193	0.011046661	2.3369	2.2673	2.408671	1061.894	1019.289	1104.666135
Scotland 2011 Men	Scotland	2011	Men	0.008786	0.008449	0.009123655	2.6362	2.5511	2.724258	878.5842	844.9002	912.3654795
E&W 1981 Women	E&W	1981	Women	0.003245	0.003145	0.003345687	1.4349	1.4202	1.449761	324.519	314.5077	334.5687
E&W 1991 Women	E&W	1991	Women	0.003561	0.003467	0.003656371	1.5807	1.5633	1.598297	356.1266	346.65	365.6371
E&W 2001 Women	E&W	2001	Women	0.003954	0.003868	0.004040811	1.8584	1.836	1.881023	395.4196	386.7829	404.0811
E&W 2011 Women	E&W	2011	Women	0.003622	0.003548	0.003695703	2.1035	2.0754	2.13204	362.1699	354.7871	369.5703
Scotland 1981 Women	Scotland	1981	Women	0.004713	0.004368	0.005062102	1.5436	1.4993	1.589246	471.3322	436.7923	506.2102
Scotland 1991 Women	Scotland	1991	Women	0.004971	0.00464	0.005305539	1.6877	1.6352	1.741814	497.1226	463.9918	530.5539
Scotland 2001 Women	Scotland	2001	Women	0.005301	0.005001	0.005602494	1.9778	1.9109	2.047052	530.0732	500.1122	560.2494
Scotland 2011 Women	Scotland	2011	Women	0.004999	0.004739	0.005259704	2.2371	2.1557	2.321455	499.8823	473.9428	525.9704

Supplement Table 3 – Interpolation of trends in the Relative Index of Inequality by sex and country

Interpolated 2008 for Scotland and 2007.5 for E&W figures		Linear interpolation	Log linear interpolation
E&W	Men	2.2336	2.231999
Scotland	Men	2.5464	2.542626
E&W	Women	2.0177	2.014243
Scotland	Women	2.1593	2.15591
Interpolated 1993 for Scotland and 1993.5 for E&W figures			
E&W	Men	1.8499	1.843737
Scotland	Men	1.944	1.934912
E&W	Women	1.6501	1.645948
Scotland	Women	1.7457	1.742076

Supplement Table 4 - Percentage change in RII and SII by sex and country for comparable time periods to Mackenbach et al.

% change over time periods		by Carstairs deprivation				By educational attainment	Mackenbach comparable figures are for 1991-5 to 2006-2010 for Scotland, and 1991-6 to 2006-9 for E&W
**RII**		**1982-1991**	**1991-2001**	**1991-2011**	**1993-2008**	**1993-2008**	**Scotland midpoints: 1993 and 2008; E&W midpoints 1993.5 and 2007.5**
E&W	Men	9.6%	1.4%		20.7%		20.7%
Scotland	Men	15.8%	10.3%		31.0%	-45.5%	31.0%
E&W	Women	10.2%			22.3%		22.3%
Scotland	Women	9.3%			23.7%	-20.0%	23.7%
**SII**							
E&W	Men	-2.7%			-12.0%		-12.0%
Scotland	Men	5.5%			-5.0%	-63.8%	-5.0%
E&W	Women	9.7%			2.1%		2.1%
Scotland	Women	5.5%			1.0%	-47.3%	1.0%

Supplement Table 5 – Comparison of trends in RII and SII by Carstairs deprivation and educational attainment

		Educational attainment			Carstairs deprivation		
		1991-1995	2006-2010	% change	1993	2008	% change
**RII**							
Scotland	Men	3.3	1.8	-45%	1.9	2.5	31%
Scotland	Women	2	1.6	-20%	1.7	2.2	24%
England & Wales	Men				1.8	2.5	38%
England & Wales	Women				1.6	2.0	23%
**Relative differences**							
Scotland	Men	1.81	1.83	1%			
Scotland	Women	1.57	1.75	11%			
England & Wales	Men	1.55	1.57	1%			
England & Wales	Women	1.46	1.46	0%			
**SII**							
Scotland	Men	1,634	591	-64%	982	934	-5%
Scotland	Women	601	317	-47%	504	509	1%
England & Wales	Men				750	660	-12%
England & Wales	Women				366	374	2%
**Absolute differences**							
Scotland	Men	681	502	-26%			
Scotland	Women	335	307	-8%			

England & Wales	Men	494	317	-36%
England & Wales	Women	254	193	-24%
