# Supplementary material for: How do trends in mortality inequalities by deprivation and education in Scotland and England & Wales compare? A repeat cross-sectional study
Source: BMJ Open. 2017 Jul 21;7(7):e017590. doi: 10.1136/bmjopen-2017-017590 (PMC5642664; doi:10.1136/bmjopen-2017-017590)
Supplement: Author's manuscript [file bmjopen-2017-017590.draft_revisions.pdf]

BMJ Open

How do trends in different measures of mortality inequalities in Scotland and England & Wales compare?

Journal:	*BMJ Open*
Manuscript ID	bmjopen-2017-017590
Article Type:	Research
Date Submitted by the Author:	03-May-2017
Complete List of Authors:	McCartney, Gerry; NHS Health Scotland<br>Popham, Frank; University of Glasgow, MRC/CSO Social and Public Health Sciences Unit<br>Katikireddi, Srinivasa; MRC/CSO Social & Public Health Sciences Unit, Evaluation of Social Interventions programme; NHS Lothian, Public Health and Health Policy<br>Walsh, David; Glasgow Centre for Population Health,<br>Schofield, Lauren; Information Services Division
**Primary Subject Heading**:	Public health
Secondary Subject Heading:	Epidemiology, Research methods
Keywords:	Health inequalities, Education, Deprivation, Scotland, England, Wales
Note: The following files were submitted by the author for peer review, but cannot be converted to PDF. You must view these files (e.g. movies) online.	
20170510 Supplement 1.xls	

SCHOLARONE™
Manuscripts

How do trends in mortality inequalities by deprivation and education in Scotland and England & Wales compare?

Gerry McCartney[1]*, Frank Popham[2], Srinivasa Vittal Katikireddi[2], David Walsh[3], Lauren Schofield[4]

[1] Public Health Science Directorate, NHS Health Scotland, Glasgow, Scotland.

[2] CSO/MRC Social and Public Health Sciences Unit, Institute of Health and Wellbeing, University of Glasgow, Glasgow, Scotland.

[3] Glasgow Centre for Population Health, Glasgow, Scotland.

[4] Public Health Intelligence, NHS National Services Scotland, Edinburgh, Scotland.

* Corresponding author. Gerry McCartney, Meridian Court, 5 Cadogan Street, Glasgow, gmccartney@nhs.net, 0141 201 2750.

Word count: 2,403 (excluding title page, abstract, summary box, references, figures, tables and notes).

For peer review only

Abstract

Objective

To compare the trends in mortality inequalities by educational attainment for with trends using area deprivation.

Setting

Scotland and England & Wales (E&W)

Participants

All people resident in Scotland and E&W between 1981 and 2011 aged 35-79 years.

Primary outcome measures

Absolute inequalities (measured using the Slope Index of Inequality (SII)) and relative inequalities (measured using the Relative Index of Inequality (RII) in all-cause mortality.

Results

Relative inequalities in mortality by area deprivation have consistently increased for men and women in Scotland and E&W between 1981-3 and 2010-2. Absolute inequalities increased for men and women in Scotland, and for women in E&W, between 1981-3 and 2000-2 before subsequently falling. For men in E&W absolute inequalities were more stable until 2000-2 before a subsequent decline. Both absolute and relative inequalities were consistently higher in men and in Scotland. These trends contrast markedly with the reported declines in mortality inequalities by educational attainment and apparent improvement of Scotland's inequalities with those in E&W.

Conclusions

Trends in health inequalities differ when assessed using different measures of socioeconomic status, reflecting either genuinely variable trends in relation to different aspects of social stratification or varying error or bias. There are particular issues with the educational attainment data in Great Britain prior to 2001 that make these education-based estimates less certain.

Keywords

Health inequalities, education, deprivation, Scotland, England & Wales.

For peer review only - http://bmjopen.bmj.com/site/about/guidelines.xhtml

Strengths and limitations of the study

This study uses data on the whole population over time stratified into ten groups to calculate mortality inequalities and therefore avoids sampling bias and aggregation of heterogeneous groups.

Unlike educational attainment, the use of area deprivation as a measure of socio-economic position avoids difficulties relating to compositional change in the population over time.

Our area-based measure of socio-economic position applies a mean deprivation score to all people living within a postcode sector which will misclassify some individuals.

The measures used to calculate the Carstairs deprivation index are a pragmatic collection of indicators drawn from the census and do not fully capture the lived reality of deprivation and may be insensitive to changes in that experience over time.

Background

Health inequalities are systematic, avoidable and unjust differences in health outcomes[1] representing substantial unnecessary mortality and morbidity across countries.[2,3] There has been a renewed policy focus on reducing health inequalities in recent years which has in turn led to debate about what should constitute success.[4]

Measures of population health tend to be seen as a relative phenomenon in that comparisons between populations are what help to contextualise whether a particular population health outcome is (relatively) good or bad. Health inequalities measures are similar in this way – it is how a health inequality measure for a population compares to those for other populations, including itself over time, that allows progress to be assessed and decisions made on priorities for action.[5] However, the health inequality measured for a single population can also be presented as either an absolute or relative difference. The interpretation of the trend in health inequalities within a country and differences in health inequality between countries can differ by whether assessed as absolute or relative difference.

Mackenbach et al. have argued from a pragmatic position that reduction in absolute difference should be considered a success because reductions in relative differences are more difficult to achieve when health is improving.[6,7] However, relative measures of inequalities in mortality improved in both Great Britain and the USA using different measures prior to the changing political context in both countries from the 1980s, despite there being improvements in the mean health of the populations at this time.[8,9] This suggests that it is possible and feasible to expect both absolute and relative measures of health inequalities to improve simultaneously given a conducive policy context.

In addition to relative and absolute measures of health inequality, there are also a variety of means of measuring socio-economic position (e.g. by income, social class, area deprivation, educational attainment) and many different health outcomes which are of interest (e.g. all-cause mortality, well-being, cause-specific morbidity, self-rated health). All of these options

For peer review only - http://bmjopen.bmj.com/site/about/guidelines.xhtml

have merit from a theoretical perspective and are likely to represent the operation of different social processes.[10,11]; [12] Monitoring trends in health inequalities using relative and absolute measures, for a number of different social ranking and outcome measures, is therefore likely to be important to provide a comprehensive understanding of health inequality trends.[13] However, it is common for there to be either no available data for many measures or for the data to have substantial limitations, leading to pragmatic decisions being made on the preferred measures for monitoring health inequality trends within and between countries.

Recent observed declines in absolute measures of inequality for mortality rates between populations ranked by educational attainment between 1990 and 2010 in Europe have been interpreted as demonstrating substantial progress in achieving a reduction in health inequality.[7] However, these reductions need to be interpreted alongside other analyses because some of the data are not nationally representative (such as those drawn from cities), and because of problems with the educational attainment data upon which some of the analyses are based.[14,15] Cautious interpretation of these data is necessary, as the priority given by policymakers to reducing health inequalities may be dependent on their understanding of these trends. Furthermore, the perceived success or failure of previous policy decisions may also be judged using these data – therefore, guiding future decision-making.

In order to assess whether the improvements in absolute inequalities reported by Mackenbach et al. for mortality inequalities by educational attainment[7] are also seen using other measures of social status, we compare the health inequalities trends for the same population strata (men and women aged 35-79 years) resident in Scotland and England & Wales using area deprivation. We then discuss the strengths and limitations of the different measures before reflecting on the implications for policy.

Methods

We report our methods here in line with the GATHER statement.[16]

Data

Individual mortality records were obtained from the National Records of Scotland (NRS) and the Office for National Statistics (ONS). Data were for residents of Scotland, and England & Wales only. Mortality records were linked to census wards (for England and Wales) and postcode sectors (for Scotland) of usual residence to allow allocation of area deprivation.

We extracted data on all deaths amongst those aged 35-79 years (to match the age strata used in Mackenbach et al.) in five year age bands, separately for men and women, for three years close to each census year (1981-1983[a], 1990-1992, 2000-2002, 2010-12) to provide more stable figures. The only exclusions were for those without a valid postcode (which tended to be for non-residents).

Socioeconomic exposure variable

[a] 1981-83 was used instead of 1980-82 because English deaths data were not available for 1980

For peer review only - http://bmjopen.bmj.com/site/about/guidelines.xhtml

For peer review only

The Carstairs deprivation score is derived from four Census variables calculated for small geographical areas: the proportion of economically active males seeking work; the proportion of people living in private households at a density of more than one person per room; the proportion of economically active males in occupational social class four or five on the Registrar General's categorisation; and the proportion of all persons in private households without access to a car or van.[17] Using the mean and standard deviations of this derived deprivation variable, postcode sectors (for Scotland, mean population 5,600 in 2011) and census wards (for England, mean population 6,540 in 2011) were ranked and divided into tenths (population weighted such that each decile had an equal population size) and each mortality record allocated a corresponding area deprivation score (1-10). The postcode sectors/wards and their deprivation scores were calculated according to the geographies and census data available at each time point. The deprivation scores for Scotland and E&W were calculated separately (i.e. the Scottish population was divided into ten equally sized units according to the deprivation score, and the E&W population was similarly divided into ten equally sized units).

Statistical analysis

We used the revised Census population estimates for each of the Census years published by NRS and the ONS as our denominator populations, multiplying the denominator by three to match the numerators (which were three years of deaths). As population undercounts were a feature of the 1981 and 1991 censuses,[18] census populations for both Scotland and England & Wales were uniformly scaled up to the revised mid-year population estimates for these census years by age group and sex.

We standardised the mortality rates using the 1976 European standard population for each tenth of the population ranked by Carstairs deprivation, for comparability with Mackenbach et al.

We calculated mortality rates for each population, sex and time period using Poisson regression, in Stata, with population as the offset and used the margins command to standardise rates.[19]. The Relative Indices of Inequality (RIIs) were also obtained from Poisson models. For the RIIs, we calculated the mid-point of the cumulative distribution of people in each decile based on the mid-year population of 35-79 year olds for each country, sex and year combination. Following Mackenbach et al., Slope Indices of Inequality (SII) were calculated as 2*ASMR*(RII-1)/(RII+1) where ASMR = age standardised mortality rate for that country, sex, year combination. The SII confidence intervals (CIs) were calculated from the CIs for the RII and ASMR.

To compare with published mortality inequalities based on educational attainment, we interpolated the trends between Census years, using log-linear interpolation for the RIIs and linear interpolation for the SIIs, to obtain estimates for the same years for which Mackenbach et al. provide data (1993, 1993/4, 2007/8 and 2008). SII and RII estimates were published by Mackenbach using educational attainment data for Scotland but not E&W (as the latter were based on only two categories of educational attainment). The published time periods for the trends in inequalities (RII and SII for Scotland; rate ratio differences and absolute differences for Scotland and E&W) by educational attainment were 1991-1995 to 2006-2010 for Scotland, and 1991-1996 to 2006-2009 for E&W.[7]

For peer review only - http://bmjopen.bmj.com/site/about/guidelines.xhtml

Simple percentage changes for Scotland and E&W for men and women and for changes in RII and SII changes were then calculated using our Carstairs deprivation-based estimates and compared with the published estimates by Mackenbach et al.

Results

Mortality rates for those aged 35-79 years were consistently higher in the more deprived areas (for men and women, and in Scotland and E&W), with a linear relationship between deprivation and mortality (Supplement 1). Relative inequalities increased steadily between 1981-3 and 2010-12 in all groups, with RIIs consistently higher in men and in Scotland. The trends for absolute inequalities across the population were more mixed: for men and women in Scotland the SIIs increased between 1981-3 and 2000-2 before falling; whilst in E&W the trends were stable for men until 2000-2 before a subsequent decline, and increased for women until 2000-2 before a subsequent decline. The SII figures were again consistently higher for men and in Scotland (Table 1 and Figure 1).

Table 1 – Relative Indices of Inequality (RII) and Slope Indices of Inequality (SII) by Carstairs deprivation decile over time (men and women aged 35-79 years)

	Males		Females	
**Year**	**RII (95% CI)**	**SII per 100,000 per year (95% CI)**	**RII (95% CI)**	**SII per 100,000 per year (95% CI)**
**England & Wales**				
1981-3	1.61 (1.59 to 1.62)	768 (753 to 784)	1.43 (1.42 to 1.45)	325 (315 to 335)
1990-2	1.76 (1.74 to 1.78)	748 (734 to 762)	1.58 (1.56 to 1.60)	356 (347 to 366)
2000-2	2.12 (2.10 to 2.14)	758 (746 to 770)	1.86 (1.84 to 1.88)	395 (387 to 404)
2010-12	2.30 (2.27 to 2.32)	608 (598 to 617)	2.10 (2.08 to 2.13)	362 (355 to 370)
**Scotland**				
1981-3	1.59 (1.55 to 1.64)	887 (833 to 941)	1.54 (1.50 to 1.59)	471 (437 to 506)
1990-2	1.85 (1.79 to 1.90)	962 (913 to 1012)	1.69 (1.64 to 1.74)	497 (464 to 531)
2000-2	2.34 (2.27 to 2.41)	1,062 (1,019 to 1,105)	1.98 (1.91 to 2.05)	530 (500 to 560)
2010-12	2.64 (2.55 to 2.72)	879 (845 to 912)	2.24 (2.16 to 2.32)	500 (474 to 526)

These trends in mortality inequalities by Carstairs deprivation are markedly different from those reported by educational attainment. Between the early 1990s and late 2000s, the reported RIIs for mortality inequalities by educational attainment[7] declined in Scotland by 45% and 20% for men and women respectively. In contrast, the RIIs by Carstairs deprivation *increased* by 31% and 24% for men and women. The reported declines in educational attainment by SII for Scotland were even larger at 64% and 47% respectively,[7] but again these were markedly different from the observed stability amongst men (5% reduction) and

For peer review only - http://bmjopen.bmj.com/site/about/guidelines.xhtml

small increase of 1% for women using Carstairs deprivation for the same period (Figure 2, Table 2).

Table 2 – Changes in Relative Index of Inequality (RII), Slope Index of Inequality (SII), absolute and relative differences in mortality for those aged 35-79 years by educational attainment and Carstairs deprivation

		Educational attainment*			Carstairs deprivation		
		1991-1995	2006-2010	% change	1993	2008	% change
**RII**							
Scotland	Men	3.3	1.8	-45%	1.9	2.5	+31%
Scotland	Women	2	1.6	-20%	1.7	2.2	+24%
**SII (per 100,000 population per year)**							
Scotland	Men	1,634	591	-64%	982	934	-5%
Scotland	Women	601	317	-47%	504	509	+1%

* Data extracted from Mackenbach 2016.

There was no trend in RII or SII for E&W by educational attainment available for direct comparison, but the reported trends in relative differences were almost zero for educational attainment whereas our analyses showed increases of 38% and 23% for relative changes in Carstairs deprivation using the RII. The trends in absolute differences by educational attainment reported for E&W were -36% and -24% for men and women.[7] The equivalent changes in absolute inequalities (using the SII) for E&W using Carstairs deprivation were -12% for men and an increase of 2% for women (Supplement 1).

Discussion

Main results

Relative inequalities in mortality by area deprivation consistently increased for men and women in Scotland and E&W between 1981-3 and 2010-2. Absolute inequalities increased for men and women in Scotland, and for women in E&W, between 1981-3 and 2000-2 before subsequently falling. For men in E&W absolute inequalities were more stable until 2000-2 before a subsequent decline. Both absolute and relative inequalities were consistently higher in men and in Scotland. The increase in absolute inequalities between 1981 and 2001, and the increase in relative inequalities between 1981 and 2011 amongst men was much greater in Scotland than in E&W. The trends in inequalities amongst women were more similar, although consistently higher in Scotland.

A comparison of trends for inequalities by educational attainment with those by area deprivation show highly divergent patterns. Between the early 1990s and late 2000s, Mackenbach et al reported large decreases in absolute and relative inequalities in Scotland, whereas the trends by area deprivation showed large increases in relative inequalities and little change in absolute inequalities for the same period. Although the data for E&W could not be compared directly, the trends in relative and absolute inequalities using deprivation do not seem to be consistent with the trends using educational attainment.

For peer review only - http://bmjopen.bmj.com/site/about/guidelines.xhtml

Strengths and weaknesses

We have used data from the whole population based on area of residency to rank the population into ten approximately equal-sized groups to calculate RIIs and SIIs for four time points. In doing so, we have been able to include the distribution of mortality across the whole population (rather than just the extremes) and have been able to avoid aggregating heterogeneous populations into large groups. Area deprivation also has the advantage of avoiding compositional change over time, in that the population can be divided and ranked easily into ten groups at each time point, unlike educational attainment data which is subject to marked secular trends in attainment which impact differentially by age.[20] The deprivation-mortality relationship we describe in this paper is not subject to the lagged selection bias effects of some other approaches.{{9735 Dowd, Jennifer B. 2014;}}

As with all area-based measures of social ranking, the socioeconomic status applied to each individual is not a function of their personal socioeconomic status, but of the mean of all those living in that area. It therefore measures a different aspect of socioeconomic status and classifies some people who are individually socioeconomically disadvantaged as non-disadvantaged and vice-versa. The measures used to create the Carstairs deprivation index may also be becoming a less sensitive means of characterising socioeconomic position as car ownership becomes increasingly common and overcrowding of housing less common in the UK.[21–23] It is also possible that there is some reverse causation in the relationship between area deprivation and health status (i.e. with poor health leading to movement to a more deprived area), although this is unlikely to be a substantial {{9736 Vittal 2017; 9370 McCartney, Gerry 2013;}}.

How this fits with the existing literature

The rise in health inequalities in Scotland and E&W and the causes of this are well described.[24] In particular, the rise in income inequalities and unemployment associated with the changed political context of the 1980s is likely to be a large part of any explanation.[21,25,26] It has been argued that the recent small declines in absolute inequalities for working age adults may be in part due to cohorts who were most adversely impacted by the application of neoliberalism and who were at high risk of death from alcohol- and drug-related deaths, suicide and violence, passing through the age of highest risk.[21,27–29] In this way, there may be some lagged effects of political exposures during the 1980s and 1990s which contributed to the observed rise in health inequalities demonstrated here. Further exploration of how health inequalities have impacted on different age groups over time is merited.

The trends we describe for area deprivation here for E&W are very similar to those reported using the National Statistics Socio-Economic Classification (NS-SEC) (Webfigure 1) and for Scotland using another small-area measure of multiple deprivation.[30,31] The similarity in the trends casts further doubt on the much more optimistic trends in inequalities described using educational attainment data to rank the population.[7]

Part of the reason for the markedly different trends may be the limited data available for educational attainment in Great Britain prior to 2001 which does not facilitate adequate stratification of the population to examine differences in outcomes.[14,15,32]. The secular trends in educational attainment, and the differences in these trends by age group, also make it important to examine narrow age groups in any cross-sectional analysis.[10]

Implications

For peer review only - http://bmjopen.bmj.com/site/about/guidelines.xhtml

Relative mortality inequalities using area deprivation in those aged 35-79 years has continuously increased since the early 1980s in Britain, and if there has been any decline in absolute inequalities since 2001 it has been small and limited to men. It is possible that there are different trends using different measures of socioeconomic status, or that one or more of the measures is subject to error or bias that has given rise to the divergent trends. There are particular issues with the educational attainment data in Great Britain prior to 2001 that make these estimates less certain. Further work to find adequate means to compare the extent of inequalities internationally should be undertaken.[33]

Notes

The Corresponding Author has the right to grant on behalf of all authors and does grant on behalf of all authors, an exclusive licence (or non exclusive for government employees) on a worldwide basis to the BMJ Publishing Group Ltd to permit this article (if accepted) to be published in BMJ editions and any other BMJPGL products and sublicences such use and exploit all subsidiary rights, as set out in our licence.

All authors have completed the Unified Competing Interest form (available on request from the corresponding author) and declare that no specific funding was received for this work. GM, DW and LS are employees of the National Health Service (NHS) in Scotland. FP and SVK are funded by the Medical Research Council and Chief Scientist's Office (MC_UU_12017/13 and SPHSU13), as part of the core funding for the MRC/CSO Social & Public Health Sciences Unit. In addition, SVK is funded by a NRS Senior Clinical Fellowship (SCAF/15/02).

We also declare that we have no financial relationships with any organisations that might have an interest in the submitted work in the previous three years and have no other relationships or activities that could appear to have influenced the submitted work.

We affirm that the manuscript is an honest, accurate, and transparent account of the study being reported; that no important aspects of the study have been omitted; and that any discrepancies from the study as planned (and, if relevant, registered) have been explained.

Ethical approval was not required for this study as the data were provided to us in aggregate form.

Data sharing – all of the available data are provided in the supplementary appendix.

GM, FP, VK and DW came up with the idea for this work. LS and FP did the data analysis. GM drafted the manuscript. All authors provided substantial edits and redrafting and approved the final draft.

References

1. Graham H. Understanding health inequalities. Maidenhead: Open University Press; 2009.

For peer review only - http://bmjopen.bmj.com/site/about/guidelines.xhtml

2. Braveman PA, Kumanykia S, Fielding J, LaVeist T, Borrell LN, Mandescheid R, Troutman A. Health Disparities and Health Equity: The Issue Is Justice. *American Journal of Public Health* 2011; **101 (supplement 1)**: S149-55.
3. Woodward A, Kawachi I. Why reduce health inequalities? *Journal of epidemiology and community health* 2000; **54**: 923-9.
4. Closing the gap in a generation: Health equity through action on the social determinants of health. Final Report of the Commission on Social Determinants of Health. 2008; .
5. Popham F. Deprivation is a relative concept? Absolutely! *Journal of epidemiology and community health* 2014; **0**: 1-2.
6. Mackenbach JP. Should we aim to reduce relative or absolute inequalities in mortality? *European Journal of Public Health* 2015; **25**(2): 185.
7. Mackenbach JP, Kulhanova I, Artnik B, et al. Changes in mortality inequalities over two decades: register based study of European countries. *BMJ* 2016; **353**.
8. Thomas B, Dorling D, Davey Smith G. Inequalities in premature mortality in Britain: observational study from 1921 to 2007. *BMJ* 2010; **341**: c3639.
9. Krieger N, Rehkopf DH, Chen JT, et al. The fall and rise of US inequities in premature mortality: 1960-2002. *PLoS Medicine / Public Library of Science* 2008; **5**(2): e46.
10. Chen JT, Beckfield J, Waterman PD, Krieger N. Can Changes in the Distributions of and Associations Between Education and Income Bias Temporal Comparisons of Health Disparities? An Exploration With Causal Graphs and Simulations. *American Journal of Epidemiology* 2013; **177**(9): 870-81.
11. Geyer S, Hemström Ö, Peter R, Vågerö D. Education, income, and occupational class cannot be used interchangeably in social epidemiology. Empirical evidence against a common practice. *Journal of Epidemiology and Community Health* 2006; **60**(9): 804-10.
12. Wright EO. Understanding Class. London: Verso; 2015.
13. Frank J, Haw S. Best Practice Guidelines for Monitoring Socioeconomic Inequalities in Health Status: Lessons from Scotland. *Millbank Quarterly* 2011; **89**(4): 658-93.
14. McCartney G, Katikireddi SV, Walsh D. Interpreting the apparent absolute reduction in educational inequalities in mortality in much of Europe: a response to Mackenbach et al. *BMJ* 2016; **353**: i1732.
15. Flanagan L, McCartney G. How robust is the calculation of health inequality trends by educational attainment in England and Wales using the Longitudinal Study? *Public health* 2015; **129**(6): 621-8.
16. Stevens GA, Alkema L, Black RE, et al. Guidelines for Accurate and Transparent Health Estimates Reporting: the GATHER statement. *PLoS Medicine* 2016; **13**(6): e1002056.
17. Carstairs V, Morris R. Deprivation and mortality: and alternative to social class? *Community Medicine* 1989; **11**(3): 210-9.
18. Dale A, Marsh C. The 1991 Census user's guide. London: HMSO; 1993.
19. Muller CJ, MacLehose RF. Estimating predicted probabilities from logistic regression: different methods correspond to different target populations. *International Journal of Epidemiology* 2014; **43**: 962-70.
20. Goujon A, S KC, Speringer M, et al. A harmonized dataset on global educational attainment between 1970 and 2060 - an analytical window into recent trends and future prospects in human capital development . *Journal of Demographic Economics* 2016; **82**(3): 315-63.

For peer review only - http://bmjopen.bmj.com/site/about/guidelines.xhtml

21. Walsh D, McCartney G, Collins C, Taulbut M, Batty GD. History, politics and vulnerability: explaining excess mortality. 2016; .

22. Schofield L, Walsh D, Munoz-Arroyo R, et al. Dying younger in Scotland: Trends in mortality and deprivation relative to England and Wales, 1981–2011. *Health and Place* 2016; **40**: 106-15.

23. Allik M, Brown D, Dundas R, Leyland AH. Developing a new small-area measure of deprivation using 2001 and 2011 census data from Scotland. *Health Place* 2016; **39**: 122-30.

24. Marmot M, Allen J, Goldblatt P, Boyce T, McNeish D, Grady M, Geddes I. Fair Society, Healthy Lives. The Marmot review. 2010; .

25. Beeston C, McCartney G, Ford J, Wimbush E, Beck S, MacDonald W, Fraser A. Health Inequalities Policy review for the Scottish Ministerial Task Force on Health Inequalities. 2013; .

26. McCartney G, Collins C, Mackenzie M. What (or who) causes health inequalities: Theories, evidence and implications? *Health Policy* 2013; **113**(3): 221-7.

27. McCartney G, Bouttell J, Craig N, et al. Explaining trends in alcohol-related harms in Scotland 1991-2011 (II): policy, social norms, the alcohol market, clinical changes and a synthesis. *Public health* 2016; **132**: 24-32.

28. McCartney G, Bouttell J, Craig N, et al. Explaining trends in alcohol-related harms in Scotland, 1991-2011 (I): the role of incomes, effects of socio-economic and political adversity and demographic change. 2016; **132**: 13-23.

29. Parkinson J, Minton J, Lewsey j, Bouttell J, McCartney G. Recent cohort effects in suicide in Scotland: a legacy of the 1980s? . *Journal of Epidemiology and Community Health* 2016; .

30. Trend in life expectancy at birth and at age 65 by socio-economic position based on the National Statistics Socio-economic Classification, England and Wales: 1982—1986 to 2007—2011. 2015; .

31. Long-Term Monitoring of Health Inequalities. 2015; .

32. Schneider SL. The application of the ISCED-97 to the UK's educational qualifications. In: Schneider SL, editor. The International Standard Classification of Education: an Evaluation of Content and Criterion Validity for 15 European Countries. Mannheim: MZES; 2008.

33. Popham F, Dibben C, Bambra C. Are health inequalities really not the smallest in the Nordic welfare states? A comparison of mortality inequality in 37 countries. *Journal of Epidemiology & Community Health* 2013; **67**(5): 412-8.

For peer review only

For peer review only - http://bmjopen.bmj.com/site/about/guidelines.xhtml

Figure 1 – Trends in SII and RII by Carstairs area deprivation (1982-2011, Scotland and England & Wales, men and women)

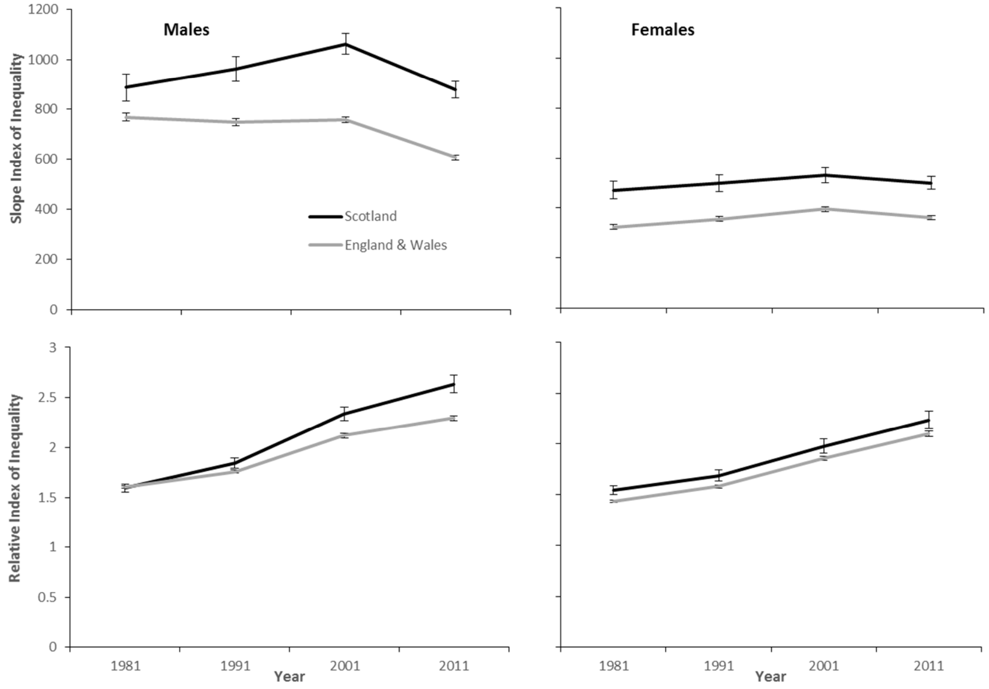

1

Figure 2 – Percentage change in SII and RII by Carstairs area deprivation and educational attainment (1993 to 2008, Scotland)

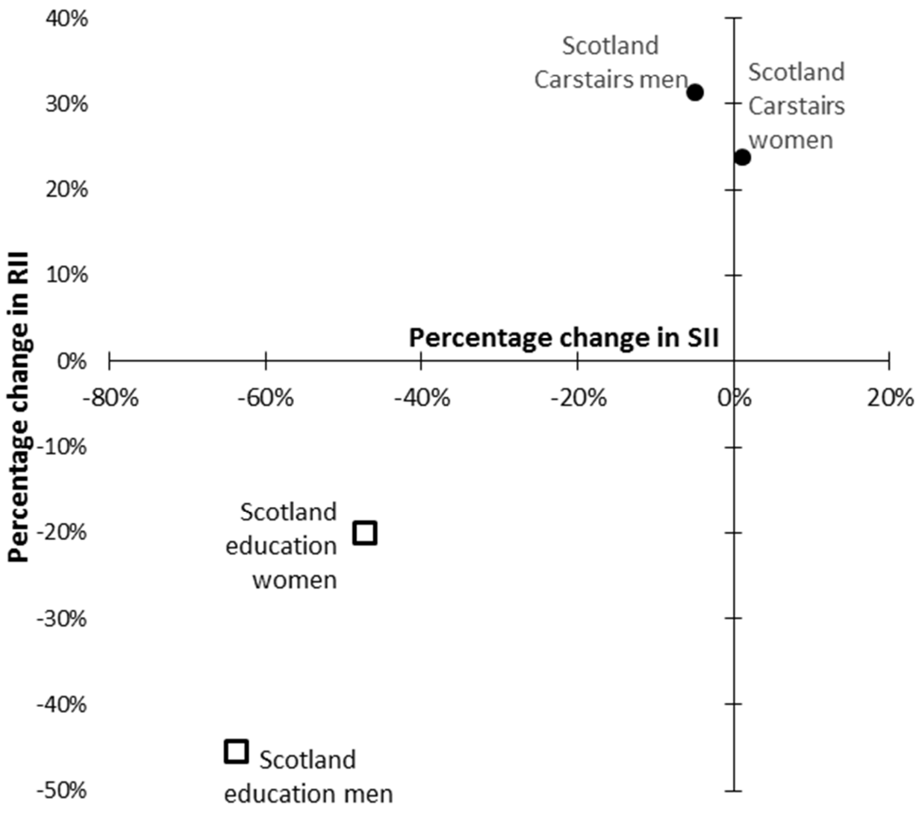

For peer review only

Table 1 – Relative Indices of Inequality (RII) and Slope Indices of Inequality (SII) by Carstairs deprivation decile over time (men and women aged 35-79 years)

	Males		Females	
Year	RII (95% CI)	SII per 100,000 per year (95% CI)	RII (95% CI)	SII per 100,000 per year (95% CI)
**England & Wales**				
1981-3	1.61 (1.59 to 1.62)	768 (753 to 784)	1.43 (1.42 to 1.45)	325 (315 to 335)
1990-2	1.76 (1.74 to 1.78)	748 (734 to 762)	1.58 (1.56 to 1.60)	356 (347 to 366)
2000-2	2.12 (2.10 to 2.14)	758 (746 to 770)	1.86 (1.84 to 1.88)	395 (387 to 404)
2010-12	2.30 (2.27 to 2.32)	608 (598 to 617)	2.10 (2.08 to 2.13)	362 (355 to 370)
**Scotland**				
1981-3	1.59 (1.55 to 1.64)	887 (833 to 941)	1.54 (1.50 to 1.59)	471 (437 to 506)
1990-2	1.85 (1.79 to 1.90)	962 (913 to 1012)	1.69 (1.64 to 1.74)	497 (464 to 531)
2000-2	2.34 (2.27 to 2.41)	1,062 (1,019 to 1,105)	1.98 (1.91 to 2.05)	530 (500 to 560)
2010-12	2.64 (2.55 to 2.72)	879 (845 to 912)	2.24 (2.16 to 2.32)	500 (474 to 526)

For peer review only

For peer review only - http://bmjopen.bmj.com/site/about/guidelines.xhtml

Table 2 – Changes in Relative Index of Inequality (RII), Slope Index of Inequality (SII), absolute and relative differences in mortality for those aged 35-79 years by educational attainment and Carstairs deprivation

		Educational attainment*			Carstairs deprivation		
		1991-1995	2006-2010	% change	1993	2008	% change
**RII**							
Scotland	Men	3.3	1.8	-45%	1.9	2.5	+31%
Scotland	Women	2	1.6	-20%	1.7	2.2	+24%
**SII (per 100,000 population per year)**							
Scotland	Men	1,634	591	-64%	982	934	-5%
Scotland	Women	601	317	-47%	504	509	+1%

* Data extracted from Mackenbach 2016.

For peer review only

STROBE 2007 (v4) Statement—Checklist of items that should be included in reports of *cross-sectional studies*

Section/Topic	Item #	Recommendation	Reported on page #
**Title and abstract**	1	(*a*) Indicate the study's design with a commonly used term in the title or the abstract	1
		(*b*) Provide in the abstract an informative and balanced summary of what was done and what was found	1
**Introduction**			
Background/rationale	2	Explain the scientific background and rationale for the investigation being reported	2-3
Objectives	3	State specific objectives, including any prespecified hypotheses	3
**Methods**			
Study design	4	Present key elements of study design early in the paper	4
Setting	5	Describe the setting, locations, and relevant dates, including periods of recruitment, exposure, follow-up, and data collection	4
Participants	6	(*a*) Give the eligibility criteria, and the sources and methods of selection of participants	4
Variables	7	Clearly define all outcomes, exposures, predictors, potential confounders, and effect modifiers. Give diagnostic criteria, if applicable	4
Data sources/ measurement	8*	For each variable of interest, give sources of data and details of methods of assessment (measurement). Describe comparability of assessment methods if there is more than one group	4
Bias	9	Describe any efforts to address potential sources of bias	4-5
Study size	10	Explain how the study size was arrived at	4
Quantitative variables	11	Explain how quantitative variables were handled in the analyses. If applicable, describe which groupings were chosen and why	4
Statistical methods	12	(*a*) Describe all statistical methods, including those used to control for confounding	4-5
		(*b*) Describe any methods used to examine subgroups and interactions	4-5
		(*c*) Explain how missing data were addressed	4-5
		(*d*) If applicable, describe analytical methods taking account of sampling strategy	n/a
		(*e*) Describe any sensitivity analyses	n/a
**Results**			

For peer review only

Participants	13*	(a) Report numbers of individuals at each stage of study—eg numbers potentially eligible, examined for eligibility, confirmed eligible, included in the study, completing follow-up, and analysed	Supplement
		(b) Give reasons for non-participation at each stage	n/a
		(c) Consider use of a flow diagram	n/a
Descriptive data	14*	(a) Give characteristics of study participants (eg demographic, clinical, social) and information on exposures and potential confounders	Supplement
		(b) Indicate number of participants with missing data for each variable of interest	n/a
Outcome data	15*	Report numbers of outcome events or summary measures	Supplement
Main results	16	(*a*) Give unadjusted estimates and, if applicable, confounder-adjusted estimates and their precision (eg, 95% confidence interval). Make clear which confounders were adjusted for and why they were included	5, 6 and supplement
		(*b*) Report category boundaries when continuous variables were categorized	n/a
		(*c*) If relevant, consider translating estimates of relative risk into absolute risk for a meaningful time period	n/a
Other analyses	17	Report other analyses done—eg analyses of subgroups and interactions, and sensitivity analyses	n/a
**Discussion**			
Key results	18	Summarise key results with reference to study objectives	7
Limitations	19	Discuss limitations of the study, taking into account sources of potential bias or imprecision. Discuss both direction and magnitude of any potential bias	8
Interpretation	20	Give a cautious overall interpretation of results considering objectives, limitations, multiplicity of analyses, results from similar studies, and other relevant evidence	8-9
Generalisability	21	Discuss the generalisability (external validity) of the study results	8-9
**Other information**			
Funding	22	Give the source of funding and the role of the funders for the present study and, if applicable, for the original study on which the present article is based	9

*Give information separately for cases and controls in case-control studies and, if applicable, for exposed and unexposed groups in cohort and cross-sectional studies.

Note: An Explanation and Elaboration article discusses each checklist item and gives methodological background and published examples of transparent reporting. The STROBE checklist is best used in conjunction with this article (freely available on the Web sites of PLoS Medicine at http://www.plosmedicine.org/, Annals of Internal Medicine at http://www.annals.org/, and Epidemiology at http://www.epidem.com/). Information on the STROBE Initiative is available at www.strobe-statement.org.

For peer review only

BMJ Open

How do trends in mortality inequalities by deprivation and education in Scotland and England & Wales compare?

Journal:	*BMJ Open*
Manuscript ID	bmjopen-2017-017590.R1
Article Type:	Research
Date Submitted by the Author:	07-Jun-2017

Complete List of Authors:	McCartney, Gerry; NHS Health Scotland<br>Popham, Frank; University of Glasgow, MRC/CSO Social and Public Health Sciences Unit<br>Katikireddi, Srinivasa; MRC/CSO Social & Public Health Sciences Unit, Evaluation of Social Interventions programme; NHS Lothian, Public Health and Health Policy<br>Walsh, David; Glasgow Centre for Population Health,<br>Schofield, Lauren; Information Services Division

**Primary Subject Heading**:	Public health
Secondary Subject Heading:	Epidemiology, Research methods
Keywords:	Health inequalities, Education, Deprivation, Scotland, England, Wales

SCHOLARONE™ Manuscripts

For peer review only

How do trends in mortality inequalities by deprivation and education in Scotland and England & Wales compare?

Gerry McCartney[1]*, Frank Popham[2], Srinivasa Vittal Katikireddi[2], David Walsh[3], Lauren Schofield[4]

[1] Public Health Science Directorate, NHS Health Scotland, Glasgow, Scotland.

[2] CSO/MRC Social and Public Health Sciences Unit, Institute of Health and Wellbeing, University of Glasgow, Glasgow, Scotland.

[3] Glasgow Centre for Population Health, Glasgow, Scotland.

[4] Public Health Intelligence, NHS National Services Scotland, Edinburgh, Scotland.

* Corresponding author. Gerry McCartney, Meridian Court, 5 Cadogan Street, Glasgow, gmccartney@nhs.net, 0141 201 2750.

Word count: 2,403 (excluding title page, abstract, summary box, references, figures, tables and notes).

For peer review only

For peer review only - http://bmjopen.bmj.com/site/about/guidelines.xhtml

Abstract

Objective

To compare the trends in mortality inequalities by educational attainment for with trends using area deprivation.

Setting

Scotland and England & Wales (E&W)

Participants

All people resident in Scotland and E&W between 1981 and 2011 aged 35-79 years.

Primary outcome measures

Absolute inequalities (measured using the Slope Index of Inequality (SII)) and relative inequalities (measured using the Relative Index of Inequality (RII)) in all-cause mortality.

Results

Relative inequalities in mortality by area deprivation have consistently increased for men and women in Scotland and E&W between 1981-3 and 2010-2. Absolute inequalities increased for men and women in Scotland, and for women in E&W, between 1981-3 and 2000-2 before subsequently falling. For men in E&W absolute inequalities were more stable until 2000-2 before a subsequent decline. Both absolute and relative inequalities were consistently higher in men and in Scotland. These trends contrast markedly with the reported declines in mortality inequalities by educational attainment and apparent improvement of Scotland's inequalities with those in E&W.

Conclusions

Trends in health inequalities differ when assessed using different measures of socioeconomic status, reflecting either genuinely variable trends in relation to different aspects of social stratification or varying error or bias. There are particular issues with the educational attainment data in Great Britain prior to 2001 that make these education-based estimates less certain.

Keywords

Health inequalities, education, deprivation, Scotland, England & Wales.

For peer review only - http://bmjopen.bmj.com/site/about/guidelines.xhtml

Strengths and limitations of the study

This study uses data on the whole population over time stratified into ten groups to calculate mortality inequalities and therefore avoids sampling bias and aggregation of heterogeneous groups.

Unlike educational attainment, the use of area deprivation as a measure of socio-economic position avoids difficulties relating to compositional change in the population over time.

Our area-based measure of socio-economic position applies a mean deprivation score to all people living within a postcode sector which will misclassify some individuals.

The measures used to calculate the Carstairs deprivation index are a pragmatic collection of indicators drawn from the census and do not fully capture the lived reality of deprivation and may be insensitive to changes in that experience over time.

Background

Health inequalities are systematic, avoidable and unjust differences in health outcomes[1] representing substantial unnecessary mortality and morbidity across countries.[2, 3] There has been a renewed policy focus on reducing health inequalities in recent years which has in turn led to debate about what should constitute success.[4]

Measures of population health tend to be seen as a relative phenomenon in that comparisons between populations are what help to contextualise whether a particular population health outcome is (relatively) good or bad. Health inequalities measures are similar in this way – it is how a health inequality measure for a population compares to those for other populations, including itself over time, that allows progress to be assessed and decisions made on priorities for action.[5] However, the health inequality measured for a single population can also be presented as either an absolute or relative difference. The interpretation of the trend in health inequalities within a country and differences in health inequality between countries can differ by whether assessed as absolute or relative difference.

Mackenbach et al. have argued from a pragmatic position that reduction in absolute difference should be considered a success because reductions in relative differences are more difficult to achieve when health is improving.[6, 7] However, relative measures of inequalities in mortality improved in both Great Britain and the USA using different measures prior to the changing political context in both countries from the 1980s, despite there being improvements in the mean health of the populations at this time.[8, 9] This suggests that it is possible and feasible to expect both absolute and relative measures of health inequalities to improve simultaneously given a conducive policy context.

In addition to relative and absolute measures of health inequality, there are also a variety of means of measuring socio-economic position (e.g. by income, social class, area deprivation, educational attainment) and many different health outcomes which are of interest (e.g. all-cause mortality, well-being, cause-specific morbidity, self-rated health). All of these options

For peer review only - http://bmjopen.bmj.com/site/about/guidelines.xhtml

have merit from a theoretical perspective and are likely to represent the operation of different social processes.[10, 11][12] Monitoring trends in health inequalities using relative and absolute measures, for a number of different social ranking and outcome measures, is therefore likely to be important to provide a comprehensive understanding of health inequality trends.[13] However, it is common for there to be either no available data for many measures or for the data to have substantial limitations, leading to pragmatic decisions being made on the preferred measures for monitoring health inequality trends within and between countries.

Recent observed declines in absolute measures of inequality for mortality rates between populations ranked by educational attainment between 1990 and 2010 in Europe have been interpreted as demonstrating substantial progress in achieving a reduction in health inequality.[7] However, these reductions need to be interpreted alongside other analyses because some of the data are not nationally representative (such as those drawn from cities), and because of problems with the educational attainment data upon which some of the analyses are based.[14, 15] Cautious interpretation of these data is necessary, as the priority given by policymakers to reducing health inequalities may be dependent on their understanding of these trends. Furthermore, the perceived success or failure of previous policy decisions may also be judged using these data – therefore, guiding future decision-making.

In order to assess whether the improvements in absolute inequalities reported by Mackenbach et al. for mortality inequalities by educational attainment[7] are also seen using other measures of social status, we compare the health inequalities trends for the same population strata (men and women aged 35-79 years) resident in Scotland and England & Wales using area deprivation. We then discuss the strengths and limitations of the different measures before reflecting on the implications for policy.

Methods

We report our methods here in line with the GATHER statement.[16]

Data

Individual mortality records were obtained from the National Records of Scotland (NRS) and the Office for National Statistics (ONS). Data were for residents of Scotland, and England & Wales only. Mortality records were linked to census wards (for England and Wales) and postcode sectors (for Scotland) of usual residence to allow allocation of area deprivation.

We extracted data on all deaths amongst those aged 35-79 years (to match the age strata used in Mackenbach et al.) in five year age bands, separately for men and women, for three years close to each census year (1981-1983[a], 1990-1992, 2000-2002, 2010-12) to provide more stable figures. The only exclusions were for those without a valid postcode (which tended to be for non-residents).

Socioeconomic exposure variable

[a] 1981-83 was used instead of 1980-82 because English deaths data were not available for 1980

For peer review only - http://bmjopen.bmj.com/site/about/guidelines.xhtml

The Carstairs deprivation score is derived from four Census variables calculated for small geographical areas: the proportion of economically active males seeking work; the proportion of people living in private households at a density of more than one person per room; the proportion of economically active males in occupational social class four or five on the Registrar General's categorisation; and the proportion of all persons in private households without access to a car or van.[17] Using the mean and standard deviations of this derived deprivation variable, postcode sectors (for Scotland, mean population 5,600 in 2011) and census wards (for England, mean population 6,540 in 2011) were ranked and divided into tenths (population weighted such that each decile had an equal population size) and each mortality record allocated a corresponding area deprivation score (1-10). The postcode sectors/wards and their deprivation scores were calculated according to the geographies and census data available at each time point. The deprivation scores for Scotland and E&W were calculated separately (i.e. the Scottish population was divided into ten equally sized units according to the deprivation score, and the E&W population was similarly divided into ten equally sized units).

<u>Statistical analysis</u>

We used the revised Census population estimates for each of the Census years published by NRS and the ONS as our denominator populations, multiplying the denominator by three to match the numerators (which were three years of deaths). As population undercounts were a feature of the 1981 and 1991 censuses,[18] census populations for both Scotland and England & Wales were constrained to the revised mid-year population estimates for these census years by age group and sex.

We standardised the mortality rates using the 1976 European standard population for each tenth of the population ranked by Carstairs deprivation, for comparability with Mackenbach et al.

We calculated mortality rates for each population, sex and time period using Poisson regression, in Stata, with population as the offset and used the margins command to standardise rates.[19]. The Relative Indices of Inequality (RIIs) were also obtained from Poisson models. For the RIIs, we calculated the mid-point of the cumulative distribution of people in each decile based on the mid-year population of 35-79 year olds for each country, sex and year combination. Following Mackenbach et al., Slope Indices of Inequality (SII) were calculated as 2*ASMR*(RII-1)/(RII+1) where ASMR = age standardised mortality rate for that country, sex, year combination. The SII confidence intervals (CIs) were calculated from the CIs for the RII and ASMR.

To compare with published mortality inequalities based on educational attainment, we interpolated the trends between Census years, using log-linear interpolation for the RIIs and linear interpolation for the SIIs, to obtain estimates for the same years for which Mackenbach et al. provide data (1993, 1993/4, 2007/8 and 2008). SII and RII estimates were published by Mackenbach using educational attainment data for Scotland but not E&W (as the latter were based on only two categories of educational attainment). The published time periods for the trends in inequalities (RII and SII for Scotland; rate ratio differences and absolute differences for Scotland and E&W) by educational attainment were 1991-1995 to 2006-2010 for Scotland, and 1991-1996 to 2006-2009 for E&W.[7]

For peer review only - http://bmjopen.bmj.com/site/about/guidelines.xhtml

Simple percentage changes for Scotland and E&W for men and women and for changes in RII and SII changes were then calculated using our Carstairs deprivation-based estimates and compared with the published estimates by Mackenbach et al.

Results

Mortality rates for those aged 35-79 years were consistently higher in the more deprived areas (for men and women, and in Scotland and E&W), with a linear relationship between deprivation and mortality (Supplement 1). Relative inequalities increased steadily between 1981-3 and 2010-12 in all groups, with RIIs consistently higher in men and in Scotland. The trends for absolute inequalities across the population were more mixed: for men and women in Scotland the SIIs increased between 1981-3 and 2000-2 before falling; whilst in E&W the trends were stable for men until 2000-2 before a subsequent decline, and increased for women until 2000-2 before a subsequent decline. The SII figures were again consistently higher for men and in Scotland (Table 1 and Figure 1).

Table 1 – Relative Indices of Inequality (RII) and Slope Indices of Inequality (SII) by Carstairs deprivation decile over time (men and women aged 35-79 years)

	Males		Females	
**Year**	**RII (95% CI)**	**SII per 100,000 per year (95% CI)**	**RII (95% CI)**	**SII per 100,000 per year (95% CI)**
**England & Wales**				
1981-3	1.61 (1.59 to 1.62)	768 (753 to 784)	1.43 (1.42 to 1.45)	325 (315 to 335)
1990-2	1.76 (1.74 to 1.78)	748 (734 to 762)	1.58 (1.56 to 1.60)	356 (347 to 366)
2000-2	2.12 (2.10 to 2.14)	758 (746 to 770)	1.86 (1.84 to 1.88)	395 (387 to 404)
2010-12	2.30 (2.27 to 2.32)	608 (598 to 617)	2.10 (2.08 to 2.13)	362 (355 to 370)
**Scotland**				
1981-3	1.59 (1.55 to 1.64)	887 (833 to 941)	1.54 (1.50 to 1.59)	471 (437 to 506)
1990-2	1.85 (1.79 to 1.90)	962 (913 to 1012)	1.69 (1.64 to 1.74)	497 (464 to 531)
2000-2	2.34 (2.27 to 2.41)	1,062 (1,019 to 1,105)	1.98 (1.91 to 2.05)	530 (500 to 560)
2010-12	2.64 (2.55 to 2.72)	879 (845 to 912)	2.24 (2.16 to 2.32)	500 (474 to 526)

These trends in mortality inequalities by Carstairs deprivation are markedly different from those reported by educational attainment. Between the early 1990s and late 2000s, the reported RIIs for mortality inequalities by educational attainment[7] declined in Scotland by 45% and 20% for men and women respectively. In contrast, the RIIs by Carstairs deprivation *increased* by 31% and 24% for men and women. The reported declines in educational attainment by SII for Scotland were even larger at 64% and 47% respectively,[7] but again these were markedly different from the observed stability amongst men (5% reduction) and

For peer review only - http://bmjopen.bmj.com/site/about/guidelines.xhtml

small increase of 1% for women using Carstairs deprivation for the same period (Figure 2, Table 2).

Table 2 – Changes in Relative Index of Inequality (RII), Slope Index of Inequality (SII), absolute and relative differences in mortality for those aged 35-79 years by educational attainment and Carstairs deprivation

		Educational attainment*			Carstairs deprivation		
		1991-1995	2006-2010	% change	1993	2008	% change
**RII**							
Scotland	Men	3.3	1.8	-45%	1.9	2.5	+31%
Scotland	Women	2	1.6	-20%	1.7	2.2	+24%
**SII (per 100,000 population per year)**							
Scotland	Men	1,634	591	-64%	982	934	-5%
Scotland	Women	601	317	-47%	504	509	+1%

* Data extracted from Mackenbach 2016.

There was no trend in RII or SII for E&W by educational attainment available for direct comparison, but the reported trends in relative differences were almost zero for educational attainment whereas our analyses showed increases of 38% and 23% for relative changes in Carstairs deprivation using the RII. The trends in absolute differences by educational attainment reported for E&W were -36% and -24% for men and women.[7] The equivalent changes in absolute inequalities (using the SII) for E&W using Carstairs deprivation were -12% for men and an increase of 2% for women (Supplement 1).

Discussion

Main results

Relative inequalities in mortality by area deprivation consistently increased for men and women in Scotland and E&W between 1981-3 and 2010-2. Absolute inequalities increased for men and women in Scotland, and for women in E&W, between 1981-3 and 2000-2 before subsequently falling. For men in E&W absolute inequalities were more stable until 2000-2 before a subsequent decline. Both absolute and relative inequalities were consistently higher in men and in Scotland. The increase in absolute inequalities between 1981 and 2001, and the increase in relative inequalities between 1981 and 2011 amongst men was much greater in Scotland than in E&W. The trends in inequalities amongst women were more similar, although consistently higher in Scotland.

A comparison of trends for inequalities by educational attainment with those by area deprivation show highly divergent patterns. Between the early 1990s and late 2000s, Mackenbach et al reported large decreases in absolute and relative inequalities in Scotland, whereas the trends by area deprivation showed large increases in relative inequalities and little change in absolute inequalities for the same period. Although the data for E&W could not be compared directly, the trends in relative and absolute inequalities using deprivation do not seem to be consistent with the trends using educational attainment.

For peer review only - http://bmjopen.bmj.com/site/about/guidelines.xhtml

Strengths and weaknesses

We have used data from the whole population based on area of residency to rank the population into ten approximately equal-sized groups to calculate RIIs and SIIs for four time points. In doing so, we have been able to include the distribution of mortality across the whole population (rather than just the extremes) and have been able to avoid aggregating heterogeneous populations into large groups. Area deprivation also has the advantage of avoiding compositional change over time, in that the population can be divided and ranked easily into ten groups at each time point, unlike educational attainment data which is subject to marked secular trends in attainment which impact differentially by age.[20] The deprivation-mortality relationship we describe in this paper is not subject to the lagged selection bias effects of some other approaches.[21]

As with all area-based measures of social ranking, the socioeconomic status applied to each individual is not a function of their personal socioeconomic status, but of the mean of all those living in that area. It therefore measures a different aspect of socioeconomic status and classifies some people who are individually socioeconomically disadvantaged as non-disadvantaged and vice-versa. The measures used to create the Carstairs deprivation index may also be becoming a less sensitive means of characterising socioeconomic position as car ownership becomes increasingly common and overcrowding of housing less common in the UK.[22-24] It is also possible that there is some reverse causation in the relationship between area deprivation and health status (particularly in relation to younger, healthier, individuals moving to less deprived areas [25, 26]). However, such population movement is unlikely to substantially undermine the results.[27, 28][29]

How this fits with the existing literature

The rise in health inequalities in Scotland and E&W and the causes of this are well described.[30] In particular, the rise in income inequalities and unemployment associated with the changed political context of the 1980s is likely to be a large part of any explanation.[22, 28, 31] It has been argued that the recent small declines in absolute inequalities for working age adults may be in part due to cohorts who were most adversely impacted by the application of neoliberalism and who were at high risk of death from alcohol- and drug-related deaths, suicide and violence, passing through the age of highest risk.[22, 32-34] In this way, there may be some lagged effects of political exposures during the 1980s and 1990s which contributed to the observed rise in health inequalities demonstrated here. Further exploration of how health inequalities have impacted on different age groups over time is merited.

The trends we describe for area deprivation here for E&W are very similar to those reported using the National Statistics Socio-Economic Classification (NS-SEC) (Webfigure 1) and for Scotland using another small-area measure of multiple deprivation.[35, 36] The similarity in the trends casts further doubt on the much more optimistic trends in inequalities described using educational attainment data to rank the population.[7]

Part of the reason for the markedly different trends may be the limited data available for educational attainment in Great Britain prior to 2001 which does not facilitate adequate stratification of the population to examine differences in outcomes.[14, 15, 37]. The secular trends in educational attainment, and the differences in these trends by age group, also make it important to examine narrow age groups in any cross-sectional analysis.[10]

For peer review only - http://bmjopen.bmj.com/site/about/guidelines.xhtml

Implications

Relative mortality inequalities using area deprivation in those aged 35-79 years has continuously increased since the early 1980s in Britain, and if there has been any decline in absolute inequalities since 2001 it has been small and limited to men. It is possible that there are different trends using different measures of socioeconomic status, or that one or more of the measures is subject to error or bias that has given rise to the divergent trends. There are particular issues with the educational attainment data in Great Britain prior to 2001 that make these estimates less certain. Further work to find adequate means to compare the extent of inequalities internationally should be undertaken.[38]

Notes

The Corresponding Author has the right to grant on behalf of all authors and does grant on behalf of all authors, an exclusive licence (or non exclusive for government employees) on a worldwide basis to the BMJ Publishing Group Ltd to permit this article (if accepted) to be published in BMJ editions and any other BMJPGL products and sublicences such use and exploit all subsidiary rights, as set out in our licence.

All authors have completed the Unified Competing Interest form (available on request from the corresponding author) and declare that no specific funding was received for this work. GM, DW and LS are employees of the National Health Service (NHS) in Scotland. FP and SVK are funded by the Medical Research Council and Chief Scientist's Office (MC_UU_12017/13 and SPHSU13), as part of the core funding for the MRC/CSO Social & Public Health Sciences Unit. In addition, SVK is funded by a NRS Senior Clinical Fellowship (SCAF/15/02).

We also declare that we have no financial relationships with any organisations that might have an interest in the submitted work in the previous three years and have no other relationships or activities that could appear to have influenced the submitted work.

We affirm that the manuscript is an honest, accurate, and transparent account of the study being reported; that no important aspects of the study have been omitted; and that any discrepancies from the study as planned (and, if relevant, registered) have been explained.

Ethical approval was not required for this study as the data were provided to us in aggregate form.

Data sharing – all of the available data are provided in the supplementary appendix.

GM, FP, VK and DW came up with the idea for this work. LS and FP did the data analysis. GM drafted the manuscript. All authors provided substantial edits and redrafting and approved the final draft.

References

1. Graham H. Understanding health inequalities. Maidenhead: Open University Press 2009.

For peer review only - http://bmjopen.bmj.com/site/about/guidelines.xhtml

2. Braveman PA, Kumanykia S, Fielding J, et al. Health Disparities and Health Equity: The Issue Is Justice. *American Journal of Public Health* 2011;101 (supplement 1):S149-55.

3. Woodward A, Kawachi I. Why reduce health inequalities?. *Journal of epidemiology and community health* 2000;54:923-9.

4. Closing the gap in a generation: Health equity through action on the social determinants of health. Final Report of the Commission on Social Determinants of Health 2008.

5. Popham F. Deprivation is a relative concept? Absolutely!. *Journal of epidemiology and community health* 2014;0:1-2.

6. Mackenbach JP. Should we aim to reduce relative or absolute inequalities in mortality?. *European Journal of Public Health* 2015;25:185 doi:10.1093/eurpub/cku217.

7. Mackenbach JP, Kulhanova I, Artnik B, et al. Changes in mortality inequalities over two decades: register based study of European countries. *BMJ* 2016;353 doi:doi: http://dx.doi.org/10.1136/bmj.i1732.

8. Thomas B, Dorling D, Davey Smith G. Inequalities in premature mortality in Britain: observational study from 1921 to 2007. *BMJ* 2010;341:c3639.

9. Krieger N, Rehkopf DH, Chen JT, et al. The fall and rise of US inequities in premature mortality: 1960-2002. *PLoS Medicine / Public Library of Science* 2008;5:e46.

10. Chen JT, Beckfield J, Waterman PD, et al. Can Changes in the Distributions of and Associations Between Education and Income Bias Temporal Comparisons of Health Disparities? An Exploration With Causal Graphs and Simulations. *American Journal of Epidemiology* 2013;177:870-81.

11. Geyer S, Hemström Ö, Peter R, et al. Education, income, and occupational class cannot be used interchangeably in social epidemiology. Empirical evidence against a common practice. *Journal of Epidemiology and Community Health* 2006;60:804-10 doi:10.1136/jech.2005.041319.

12. Wright EO. Understanding Class. London: Verso 2015.

13. Frank J, Haw S. Best Practice Guidelines for Monitoring Socioeconomic Inequalities in Health Status: Lessons from Scotland. *Millbank Quarterly* 2011;89:658-93.

14. McCartney G, Katikireddi SV, Walsh D. Interpreting the apparent absolute reduction in educational inequalities in mortality in much of Europe: a response to Mackenbach et al. *BMJ* 2016;353:i1732.

15. Flanagan L, McCartney G. How robust is the calculation of health inequality trends by educational attainment in England and Wales using the Longitudinal Study?. *Public health* 2015;129:621-8.

16. Stevens GA, Alkema L, Black RE, et al. Guidelines for Accurate and Transparent Health Estimates Reporting: the GATHER statement. *PLoS Medicine* 2016;13:e1002056 doi:10.1371/journal.pmed.1002056.

For peer review only - http://bmjopen.bmj.com/site/about/guidelines.xhtml

17. Carstairs V, Morris R. Deprivation and mortality: and alternative to social class?. *Community Medicine* 1989;11:210-9.

18. Dale A, Marsh C. The 1991 Census user's guide. London: HMSO 1993.

19. Muller CJ, MacLehose RF. Estimating predicted probabilities from logistic regression: different methods correspond to different target populations. *International Journal of Epidemiology* 2014;43:962-70.

20. Goujon A, S KC, Speringer M, et al. A harmonized dataset on global educational attainment between 1970 and 2060 - an analytical window into recent trends and future prospects in human capital development . *Journal of Demographic Economics* 2016;82:315-63 doi:https://doi.org/10.1017/dem.2016.10.

21. Dowd JB, Hamoudi A. Is life expectancy really falling for groups of low socio-economic status? Lagged selection bias and artefactual trends in mortality. *International Journal of Epidemiology* 2014;43:983-8 doi:10.1093/ije/dyu120.

22. Walsh D, McCartney G, Collins C, et al. History, politics and vulnerability: explaining excess mortality 2016.

23. Schofield L, Walsh D, Munoz-Arroyo R, et al. Dying younger in Scotland: Trends in mortality and deprivation relative to England and Wales, 1981–2011. *Health and Place* 2016;40:106-15.

24. Allik M, Brown D, Dundas R, et al. Developing a new small-area measure of deprivation using 2001 and 2011 census data from Scotland. *Health Place* 2016;39:122-30 doi:http://dx.doi.org/10.1016/j.healthplace.2016.03.006.

25. Norman P, Boyle P. Are health inequalities between differently deprived areas evident at different ages? A longitudinal study of census records in England and Wales, 1991–2001. Health Place 2014;26:88-93 doi:https://doi.org/10.1016/j.healthplace.2013.12.010.

26. Norman P, Boyle P, Rees P. Selective migration, health and deprivation: a longitudinal analysis. Soc Sci Med 2005;60:2755-71 doi:https://doi.org/10.1016/j.socscimed.2004.11.008.

27. Katikireddi SV, Whitley E, Lewsey J, et al. Socioeconomic status as an effect modifier of alcohol consumption and harm: analysis of linked cohort data. *Lancet Public Health* 2017 doi:http://dx.doi.org/10.1016/S2468-2667(17)30078-6.

28. McCartney G, Collins C, Mackenzie M. What (or who) causes health inequalities: Theories, evidence and implications?. *Health Policy* 2013;113:221-7.

29. Popham F, Boyle P, O'Reilly D, et al. Selective internal migration. Does it explain Glasgow's worsening mortality record? Scottish Longitudinal Study (SLS) Research Paper Series. Research working paper 5 2009. 30. Marmot M, Allen J, Goldblatt P, et al. Fair Society, Healthy Lives. The Marmot review 2010.

31. Beeston C, McCartney G, Ford J, et al. Health Inequalities Policy review for the Scottish Ministerial Task Force on Health Inequalities 2013.

For peer review only - http://bmjopen.bmj.com/site/about/guidelines.xhtml

For peer review only

32. McCartney G, Bouttell J, Craig N, et al. Explaining trends in alcohol-related harms in Scotland 1991-2011 (II): policy, social norms, the alcohol market, clinical changes and a synthesis. *Public health* 2016;132:24-32.

33. McCartney G, Bouttell J, Craig N, et al. Explaining trends in alcohol-related harms in Scotland, 1991-2011 (I): the role of incomes, effects of socio-economic and political adversity and demographic change 2016;132:13-23.

34. Parkinson J, Minton J, Lewsey j, et al. Recent cohort effects in suicide in Scotland: a legacy of the 1980s? . *Journal of Epidemiology and Community Health* 2016 doi:10.1136/jech-2016-207296.

35. Trend in life expectancy at birth and at age 65 by socio-economic position based on the National Statistics Socio-economic Classification, England and Wales: 1982—1986 to 2007—2011 2015.

36. Long-Term Monitoring of Health Inequalities 2015. Edinburgh, Scottish Government, 2016.

37. Schneider SL. The application of the ISCED-97 to the UK's educational qualifications. In: Schneider SL, ed. The International Standard Classification of Education: an Evaluation of Content and Criterion Validity for 15 European Countries. Mannheim: MZES 2008.

38. Popham F, Dibben C, Bambra C. Are health inequalities really not the smallest in the Nordic welfare states? A comparison of mortality inequality in 37 countries. *Journal of Epidemiology & Community Health* 2013; 67:412-8 doi:10.1136/jech-2012-201525.

For peer review only

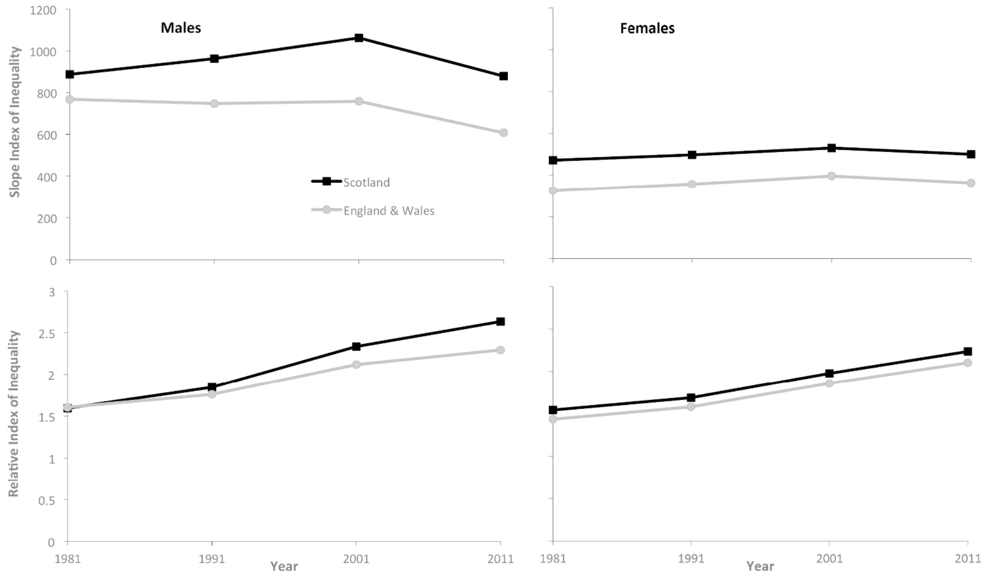

Figure 1 – Trends in SII and RII by Carstairs area deprivation (1982-2011, Scotland and England & Wales, men and women

239x139mm (300 x 300 DPI)

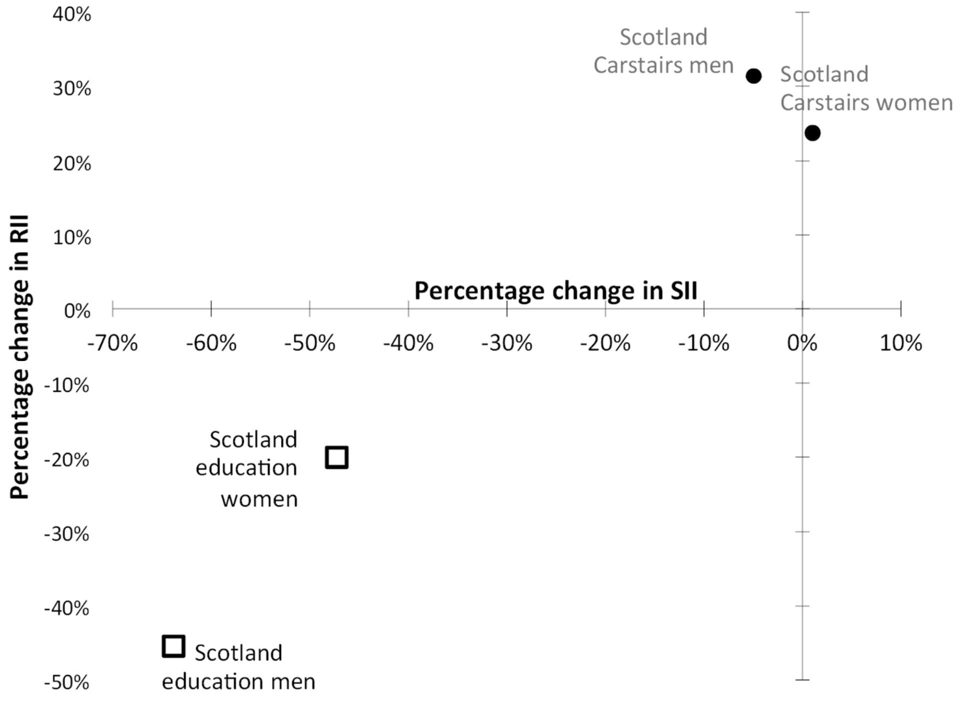

Figure 2 – Percentage change in SII and RII by Carstairs area deprivation and educational attainment (1993 to 2008, Scotland)

125x94mm (300 x 300 DPI)

Crude and age-adjusted rates

group	country	sex	year	decile	crude_rate	age_adj_rate	age_adj_rate_lower_CI	age_adj_rate_upper_CI	Age-adjusted mortality per 100,000 per year
E&W 1981 Women	E&W	Women	1981	1	0.009612889	0.007755622	0.007672978	0.007838266	775.5622
E&W 1981 Women	E&W	Women	1981	2	0.010476029	0.007906884	0.007825425	0.007988344	790.6884
E&W 1981 Women	E&W	Women	1981	3	0.011498301	0.008332084	0.008249331	0.008414837	833.2084
E&W 1981 Women	E&W	Women	1981	4	0.011785442	0.008367835	0.008285735	0.008449935	836.7835
E&W 1981 Women	E&W	Women	1981	5	0.012349643	0.008772567	0.008687877	0.008857258	877.2567
E&W 1981 Women	E&W	Women	1981	6	0.012604976	0.008939335	0.008853824	0.009024845	893.9335
E&W 1981 Women	E&W	Women	1981	7	0.013215572	0.009388286	0.009299824	0.009476748	938.8286
E&W 1981 Women	E&W	Women	1981	8	0.013763275	0.009750938	0.00966083	0.009841046	975.0938
E&W 1981 Women	E&W	Women	1981	9	0.014304536	0.01028942	0.010195368	0.010383472	1028.942
E&W 1981 Women	E&W	Women	1981	10	0.015758992	0.01145973	0.011357135	0.011562326	1145.973
E&W 1991 Women	E&W	Women	1991	1	0.007720282	0.006422884	0.006349762	0.006496005	642.2884
E&W 1991 Women	E&W	Women	1991	2	0.008475934	0.006741511	0.006666873	0.00681615	674.1511
E&W 1991 Women	E&W	Women	1991	3	0.009114578	0.006902071	0.006827492	0.006976651	690.2071
E&W 1991 Women	E&W	Women	1991	4	0.009720711	0.00722439	0.007147987	0.007300793	722.439
E&W 1991 Women	E&W	Women	1991	5	0.010306351	0.007550758	0.007472328	0.007629189	755.0758
E&W 1991 Women	E&W	Women	1991	6	0.010769808	0.007825069	0.007744471	0.007905667	782.5069
E&W 1991 Women	E&W	Women	1991	7	0.011524274	0.008384602	0.008300254	0.00846895	838.4602
E&W 1991 Women	E&W	Women	1991	8	0.012323026	0.008901691	0.008813651	0.00898973	890.1691
E&W 1991 Women	E&W	Women	1991	9	0.012662662	0.009270408	0.009179103	0.009361713	927.0408
E&W 1991 Women	E&W	Women	1991	10	0.013763391	0.010391625	0.010289922	0.010493329	1039.163
E&W 2001 Women	E&W	Women	2001	1	0.00579012	0.004941082	0.004879425	0.005002738	494.1082
E&W 2001 Women	E&W	Women	2001	2	0.00664026	0.005331322	0.005268285	0.005394358	533.1322
E&W 2001 Women	E&W	Women	2001	3	0.007079005	0.005542715	0.005478404	0.005607025	554.2715
E&W 2001 Women	E&W	Women	2001	4	0.007456318	0.005833322	0.005766699	0.005899944	583.3322
E&W 2001 Women	E&W	Women	2001	5	0.008136969	0.00624155	0.006172043	0.006311057	624.155
E&W 2001 Women	E&W	Women	2001	6	0.008767891	0.006741833	0.006668222	0.006815443	674.1833
E&W 2001 Women	E&W	Women	2001	7	0.009152499	0.007223277	0.007145289	0.007301265	722.3277
E&W 2001 Women	E&W	Women	2001	8	0.009599881	0.007714746	0.007632123	0.00779737	771.4746
E&W 2001 Women	E&W	Women	2001	9	0.010201663	0.008228177	0.008141838	0.008314516	822.8177
E&W 2001 Women	E&W	Women	2001	10	0.010587698	0.009121022	0.009022989	0.009219056	912.1022

For peer review only

E&W 2011 Women	E&W	Women	2011	1	0.00452512	0.003655192	0.003606717	0.003703668	365.5192
E&W 2011 Women	E&W	Women	2011	2	0.005046366	0.004001625	0.003950601	0.004052648	400.1625
E&W 2011 Women	E&W	Women	2011	3	0.005369199	0.004198358	0.004145849	0.004250867	419.8358
E&W 2011 Women	E&W	Women	2011	4	0.005613362	0.004515388	0.004459541	0.004571235	451.5388
E&W 2011 Women	E&W	Women	2011	5	0.006173427	0.004944128	0.004884818	0.005003437	494.4128
E&W 2011 Women	E&W	Women	2011	6	0.00644843	0.005288613	0.005225253	0.005351974	528.8613
E&W 2011 Women	E&W	Women	2011	7	0.006972373	0.005895116	0.005826416	0.005963816	589.5116
E&W 2011 Women	E&W	Women	2011	8	0.007102966	0.006229234	0.006155617	0.006302851	622.9234
E&W 2011 Women	E&W	Women	2011	9	0.00742091	0.006763139	0.006682834	0.006843444	676.3139
E&W 2011 Women	E&W	Women	2011	10	0.007504293	0.007255963	0.007166445	0.007345481	725.5963
E&W 1981 Men	E&W	Men	1981	1	0.013836215	0.013282844	0.01316358	0.013402108	1328.284
E&W 1981 Men	E&W	Men	1981	2	0.015771348	0.014010972	0.013892168	0.014129777	1401.097
E&W 1981 Men	E&W	Men	1981	3	0.01732869	0.014876871	0.014755229	0.014998513	1487.687
E&W 1981 Men	E&W	Men	1981	4	0.017783471	0.014983149	0.014861826	0.015104471	1498.315
E&W 1981 Men	E&W	Men	1981	5	0.018750443	0.01581156	0.015685979	0.015937141	1581.156
E&W 1981 Men	E&W	Men	1981	6	0.019528808	0.016484809	0.016356086	0.016613532	1648.481
E&W 1981 Men	E&W	Men	1981	7	0.020527071	0.017374747	0.017241317	0.017508177	1737.475
E&W 1981 Men	E&W	Men	1981	8	0.021516646	0.018263609	0.018126787	0.018400431	1826.361
E&W 1981 Men	E&W	Men	1981	9	0.022342745	0.019196938	0.019054611	0.019339266	1919.694
E&W 1981 Men	E&W	Men	1981	10	0.024349277	0.021351602	0.021197785	0.02150542	2135.16
E&W 1991 Men	E&W	Men	1991	1	0.01103878	0.010488629	0.010388499	0.010588758	1048.863
E&W 1991 Men	E&W	Men	1991	2	0.01226422	0.011254249	0.011150527	0.011357971	1125.425
E&W 1991 Men	E&W	Men	1991	3	0.01325232	0.01165966	0.011554955	0.011764365	1165.966
E&W 1991 Men	E&W	Men	1991	4	0.014156857	0.012279371	0.012171604	0.012387137	1227.937
E&W 1991 Men	E&W	Men	1991	5	0.014950535	0.012817366	0.012706626	0.012928105	1281.737
E&W 1991 Men	E&W	Men	1991	6	0.015800782	0.013509515	0.013394194	0.013624835	1350.951
E&W 1991 Men	E&W	Men	1991	7	0.017159268	0.014640691	0.014519764	0.014761619	1464.069
E&W 1991 Men	E&W	Men	1991	8	0.018230566	0.015567663	0.015441112	0.015694214	1556.766
E&W 1991 Men	E&W	Men	1991	9	0.019024402	0.016314241	0.016183029	0.016445452	1631.424
E&W 1991 Men	E&W	Men	1991	10	0.021058194	0.018420098	0.018274859	0.018565338	1842.01
E&W 2001 Men	E&W	Men	2001	1	0.008202026	0.007523487	0.007444123	0.007602851	752.3487
E&W 2001 Men	E&W	Men	2001	2	0.009261247	0.008104411	0.00802296	0.008185863	810.4411

For peer review only

E&W 2001 Men	E&W	Men	2001	3	0.010043937	0.008723544	0.008638119	0.008808969	872.3544
E&W 2001 Men	E&W	Men	2001	4	0.010621459	0.009262298	0.009173214	0.009351383	926.2298
E&W 2001 Men	E&W	Men	2001	5	0.011491073	0.009944548	0.009851023	0.010038073	994.4548
E&W 2001 Men	E&W	Men	2001	6	0.01228122	0.010788395	0.010688931	0.010887858	1078.839
E&W 2001 Men	E&W	Men	2001	7	0.01297947	0.011653791	0.011547806	0.011759775	1165.379
E&W 2001 Men	E&W	Men	2001	8	0.013801846	0.01256548	0.012453197	0.012677762	1256.548
E&W 2001 Men	E&W	Men	2001	9	0.015039413	0.013770089	0.013651084	0.013889093	1377.009
E&W 2001 Men	E&W	Men	2001	10	0.016328106	0.015628966	0.015493243	0.01576469	1562.897
E&W 2011 Men	E&W	Men	2011	1	0.006531269	0.005324398	0.005264408	0.005384389	532.4398
E&W 2011 Men	E&W	Men	2011	2	0.007209251	0.005844454	0.005780896	0.005908012	584.4454
E&W 2011 Men	E&W	Men	2011	3	0.00771371	0.006289675	0.006222895	0.006356455	628.9675
E&W 2011 Men	E&W	Men	2011	4	0.008046375	0.006782732	0.006711645	0.00685382	678.2732
E&W 2011 Men	E&W	Men	2011	5	0.008744033	0.007441684	0.00736579	0.007517578	744.1684
E&W 2011 Men	E&W	Men	2011	6	0.009159381	0.008064742	0.007983004	0.00814648	806.4742
E&W 2011 Men	E&W	Men	2011	7	0.009774983	0.008875782	0.008787907	0.008963658	887.5782
E&W 2011 Men	E&W	Men	2011	8	0.010086383	0.009604045	0.009508757	0.009699333	960.4045
E&W 2011 Men	E&W	Men	2011	9	0.010646058	0.010524807	0.010420662	0.010628953	1052.481
E&W 2011 Men	E&W	Men	2011	10	0.011066971	0.011640176	0.011522596	0.011757755	1164.018
Scotland 1981 Women	Scotland	Women	1981	1	0.010525108	0.008241884	0.007898118	0.008585651	824.1884
Scotland 1981 Women	Scotland	Women	1981	2	0.013282962	0.00911192	0.008803342	0.009420497	911.192
Scotland 1981 Women	Scotland	Women	1981	3	0.013353083	0.009459393	0.009169682	0.009749104	945.9393
Scotland 1981 Women	Scotland	Women	1981	4	0.014387775	0.01028801	0.009965086	0.010610935	1028.801
Scotland 1981 Women	Scotland	Women	1981	5	0.014238784	0.010217297	0.009906958	0.010527636	1021.73
Scotland 1981 Women	Scotland	Women	1981	6	0.014408361	0.01067589	0.010387509	0.010964272	1067.589
Scotland 1981 Women	Scotland	Women	1981	7	0.014307882	0.011156961	0.010869841	0.011444081	1115.696
Scotland 1981 Women	Scotland	Women	1981	8	0.015429545	0.011470359	0.011197754	0.011742964	1147.036
Scotland 1981 Women	Scotland	Women	1981	9	0.015946955	0.012255514	0.011984849	0.012526179	1225.551
Scotland 1981 Women	Scotland	Women	1981	10	0.017832188	0.01393502	0.013631905	0.014238135	1393.502
Scotland 1991 Women	Scotland	Women	1991	1	0.007778107	0.006961045	0.006652506	0.007269583	696.1045
Scotland 1991 Women	Scotland	Women	1991	2	0.010644362	0.008145254	0.00784715	0.008443357	814.5254
Scotland 1991 Women	Scotland	Women	1991	3	0.010573639	0.008200427	0.007914969	0.008485886	820.0427
Scotland 1991 Women	Scotland	Women	1991	4	0.011319426	0.008554462	0.008286397	0.008822528	855.4462

For peer review only

Scotland 1991 Women	Scotland	Women	1991	5	0.011419762	0.008703245	0.008439098	0.008967393	870.3245
Scotland 1991 Women	Scotland	Women	1991	6	0.011959812	0.00912001	0.008847431	0.009392588	912.001
Scotland 1991 Women	Scotland	Women	1991	7	0.01254952	0.009723669	0.009461309	0.009986029	972.3669
Scotland 1991 Women	Scotland	Women	1991	8	0.014283347	0.010841657	0.010559872	0.011123442	1084.166
Scotland 1991 Women	Scotland	Women	1991	9	0.014621061	0.010985495	0.010716051	0.011254939	1098.55
Scotland 1991 Women	Scotland	Women	1991	10	0.017393045	0.013147151	0.012832815	0.013461487	1314.715
Scotland 2001 Women	Scotland	Women	2001	1	0.006054715	0.005369293	0.005119773	0.005618813	536.9293
Scotland 2001 Women	Scotland	Women	2001	2	0.007473842	0.006167007	0.005932055	0.006401959	616.7007
Scotland 2001 Women	Scotland	Women	2001	3	0.007940882	0.006723365	0.006462115	0.006984616	672.3365
Scotland 2001 Women	Scotland	Women	2001	4	0.008454126	0.006752374	0.00650612	0.006998627	675.2374
Scotland 2001 Women	Scotland	Women	2001	5	0.008833125	0.006991448	0.006751617	0.007231278	699.1448
Scotland 2001 Women	Scotland	Women	2001	6	0.009997091	0.007983593	0.007739673	0.008227513	798.3593
Scotland 2001 Women	Scotland	Women	2001	7	0.01012218	0.008270903	0.008026704	0.008515101	827.0903
Scotland 2001 Women	Scotland	Women	2001	8	0.011031647	0.008757081	0.00851662	0.008997541	875.7081
Scotland 2001 Women	Scotland	Women	2001	9	0.012063884	0.009551886	0.009302581	0.00980119	955.1886
Scotland 2001 Women	Scotland	Women	2001	10	0.014525514	0.011720127	0.011420843	0.01201941	1172.013
Scotland 2011 Women	Scotland	Women	2011	1	0.005188653	0.004390383	0.00421379	0.004566976	439.0383
Scotland 2011 Women	Scotland	Women	2011	2	0.005999477	0.004874167	0.004690536	0.005057797	487.4167
Scotland 2011 Women	Scotland	Women	2011	3	0.006649881	0.005285443	0.005095168	0.005475718	528.5443
Scotland 2011 Women	Scotland	Women	2011	4	0.007424119	0.005853295	0.005651062	0.006055528	585.3295
Scotland 2011 Women	Scotland	Women	2011	5	0.007461833	0.006041823	0.005833749	0.006249897	604.1823
Scotland 2011 Women	Scotland	Women	2011	6	0.00826023	0.006869481	0.006642285	0.007096677	686.9481
Scotland 2011 Women	Scotland	Women	2011	7	0.008382318	0.006941867	0.006709153	0.007174581	694.1867
Scotland 2011 Women	Scotland	Women	2011	8	0.00930957	0.007758332	0.00750849	0.008008174	775.8332
Scotland 2011 Women	Scotland	Women	2011	9	0.010235269	0.008278501	0.008024174	0.008532828	827.8501
Scotland 2011 Women	Scotland	Women	2011	10	0.01181909	0.010062415	0.009766534	0.010358295	1006.241
Scotland 1981 Men	Scotland	Men	1981	1	0.014829473	0.014645599	0.014114115	0.015177084	1464.56
Scotland 1981 Men	Scotland	Men	1981	2	0.018356204	0.01548973	0.015027761	0.0159517	1548.973
Scotland 1981 Men	Scotland	Men	1981	3	0.019749633	0.017033235	0.016591014	0.017475456	1703.323
Scotland 1981 Men	Scotland	Men	1981	4	0.020395399	0.0174934	0.017016793	0.017970006	1749.34
Scotland 1981 Men	Scotland	Men	1981	5	0.020899901	0.018031108	0.017562668	0.018499549	1803.111
Scotland 1981 Men	Scotland	Men	1981	6	0.021253547	0.018943333	0.018504819	0.019381847	1894.333

For peer review only

Scotland 1981 Men	Scotland	Men	1981	7	0.021440803	0.019655468	0.019223348	0.020087587	1965.547
Scotland 1981 Men	Scotland	Men	1981	8	0.023382957	0.020479833	0.020064179	0.020895487	2047.983
Scotland 1981 Men	Scotland	Men	1981	9	0.023369206	0.021274203	0.020864997	0.021683409	2127.42
Scotland 1981 Men	Scotland	Men	1981	10	0.026627273	0.02451257	0.024055239	0.0249699	2451.257
Scotland 1991 Men	Scotland	Men	1991	1	0.010887721	0.011516785	0.011076989	0.011956582	1151.679
Scotland 1991 Men	Scotland	Men	1991	2	0.01369037	0.012734436	0.012317722	0.01315115	1273.444
Scotland 1991 Men	Scotland	Men	1991	3	0.014336067	0.013406395	0.013000268	0.013812523	1340.64
Scotland 1991 Men	Scotland	Men	1991	4	0.015868404	0.014157409	0.013775834	0.014538983	1415.741
Scotland 1991 Men	Scotland	Men	1991	5	0.016307012	0.014769597	0.014385004	0.01515419	1476.96
Scotland 1991 Men	Scotland	Men	1991	6	0.016837016	0.015184471	0.014790523	0.015578419	1518.447
Scotland 1991 Men	Scotland	Men	1991	7	0.018238995	0.016798337	0.016411561	0.017185113	1679.834
Scotland 1991 Men	Scotland	Men	1991	8	0.019793804	0.01748933	0.017087907	0.017890753	1748.933
Scotland 1991 Men	Scotland	Men	1991	9	0.021303564	0.018721615	0.018327746	0.019115484	1872.161
Scotland 1991 Men	Scotland	Men	1991	10	0.025642796	0.022471745	0.02201256	0.02293093	2247.174
Scotland 2001 Men	Scotland	Men	2001	1	0.008092963	0.007992258	0.007663932	0.008320584	799.2258
Scotland 2001 Men	Scotland	Men	2001	2	0.009987633	0.009536619	0.009219267	0.009853972	953.6619
Scotland 2001 Men	Scotland	Men	2001	3	0.011075126	0.010522013	0.010168719	0.010875308	1052.201
Scotland 2001 Men	Scotland	Men	2001	4	0.011944081	0.01074405	0.010409199	0.011078901	1074.405
Scotland 2001 Men	Scotland	Men	2001	5	0.012804428	0.011731175	0.011391259	0.012071091	1173.117
Scotland 2001 Men	Scotland	Men	2001	6	0.01411566	0.012887867	0.012549888	0.013225846	1288.787
Scotland 2001 Men	Scotland	Men	2001	7	0.01443252	0.013449304	0.013108893	0.013789715	1344.93
Scotland 2001 Men	Scotland	Men	2001	8	0.016276902	0.015007962	0.014660355	0.015355569	1500.796
Scotland 2001 Men	Scotland	Men	2001	9	0.017687748	0.016079247	0.015721943	0.01643655	1607.925
Scotland 2001 Men	Scotland	Men	2001	10	0.022427071	0.020676718	0.02023799	0.021115446	2067.672
Scotland 2011 Men	Scotland	Men	2011	1	0.006641684	0.005838869	0.00562563	0.006052108	583.8869
Scotland 2011 Men	Scotland	Men	2011	2	0.008213557	0.006959545	0.00673004	0.007189049	695.9545
Scotland 2011 Men	Scotland	Men	2011	3	0.009326207	0.007787182	0.007543687	0.008030678	778.7182
Scotland 2011 Men	Scotland	Men	2011	4	0.009918607	0.008362189	0.008105543	0.008618836	836.2189
Scotland 2011 Men	Scotland	Men	2011	5	0.010430598	0.009189839	0.008916447	0.00946323	918.9839
Scotland 2011 Men	Scotland	Men	2011	6	0.01124958	0.010073004	0.009780454	0.010365554	1007.3
Scotland 2011 Men	Scotland	Men	2011	7	0.011551229	0.010486904	0.010182382	0.010791425	1048.69
Scotland 2011 Men	Scotland	Men	2011	8	0.012963478	0.011963323	0.011630806	0.012295839	1196.332

For peer review only

Scotland 2011 Men	Scotland	Men	2011	9	0.014115218	0.01268553	0.01234699	0.01302407	1268.553
Scotland 2011 Men	Scotland	Men	2011	10	0.017255414	0.01641974	0.016013286	0.016826194	1641.974

For peer review only

Descriptive graphs – age-adjusted mortality rates per 100,000 per year by sex, deprivation tenth and country

For peer review only

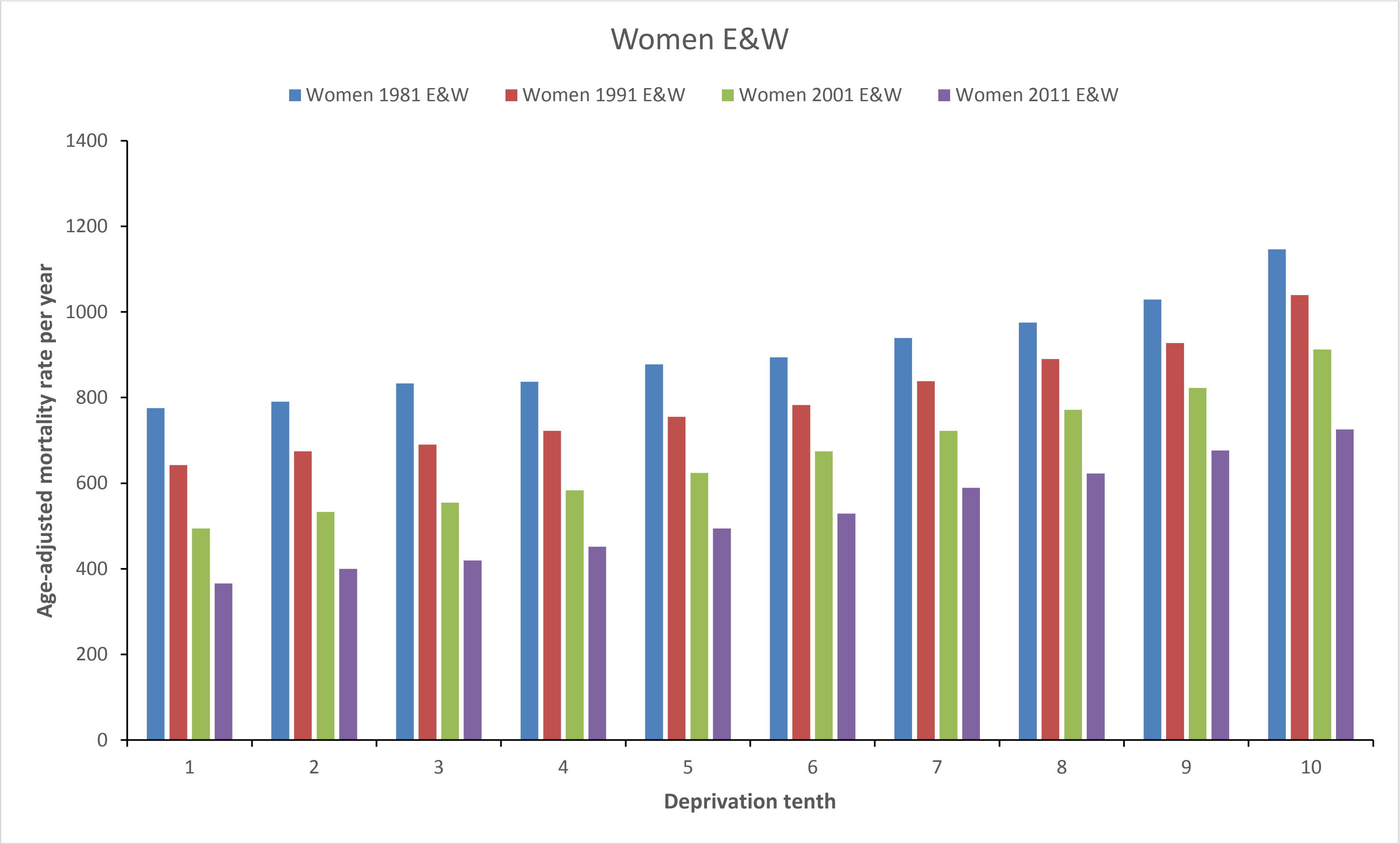
Women E&W
Women 1981 E&W
Women 1991 E&W
Women 2001 E&W
Women 2011 E&W
Age-adjusted mortality rate per year
1400
1200
1000
800
600
400
200
0
1
2
3
4
5
6
7
8
9
10
Deprivation tenth

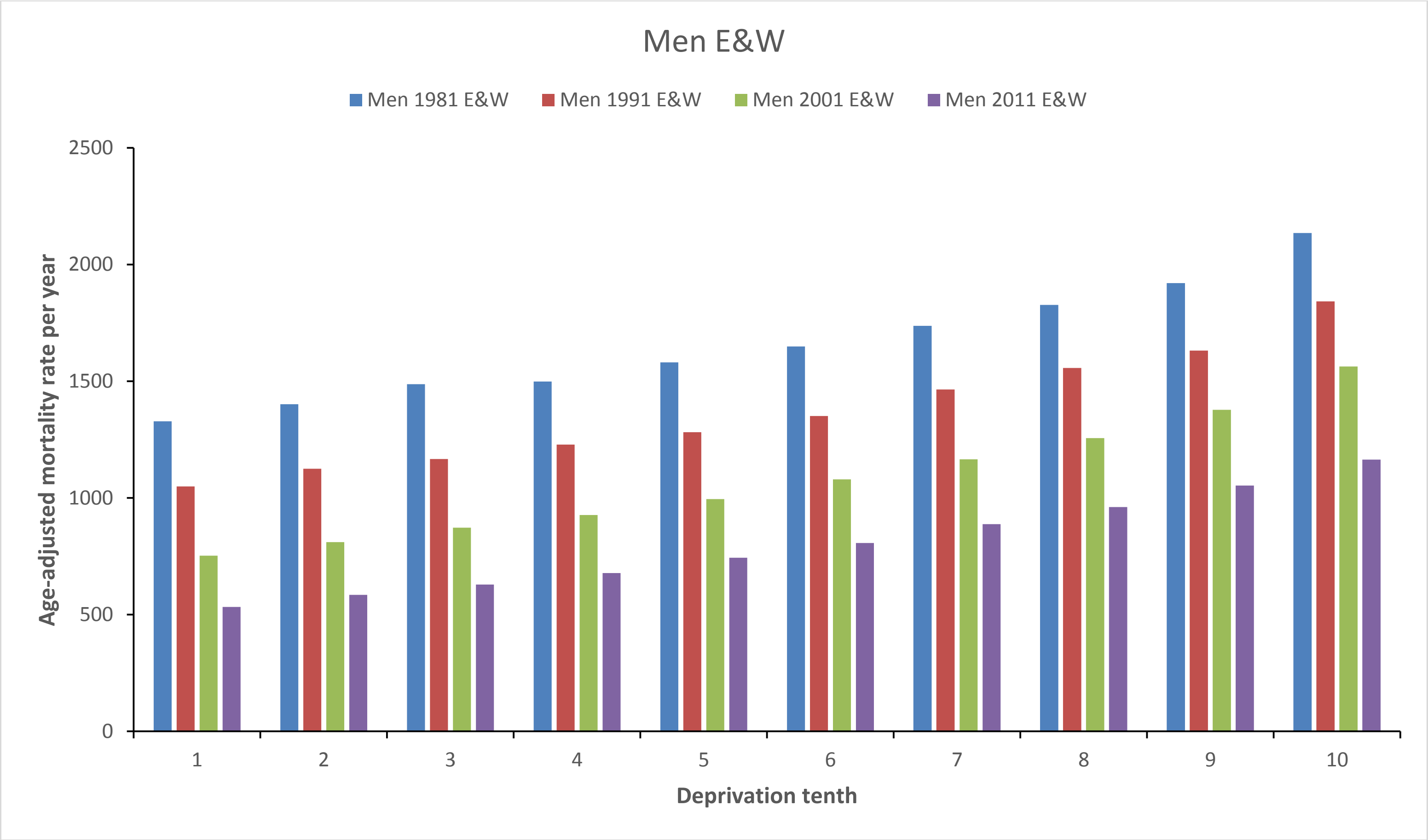
Men E&W
Men 1981 E&W
Men 1991 E&W
Men 2001 E&W
Men 2011 E&W
Age-adjusted mortality rate per year
2500
2000
1500
1000
500
0
1
2
3
4
5
6
7
8
9
10
Deprivation tenth

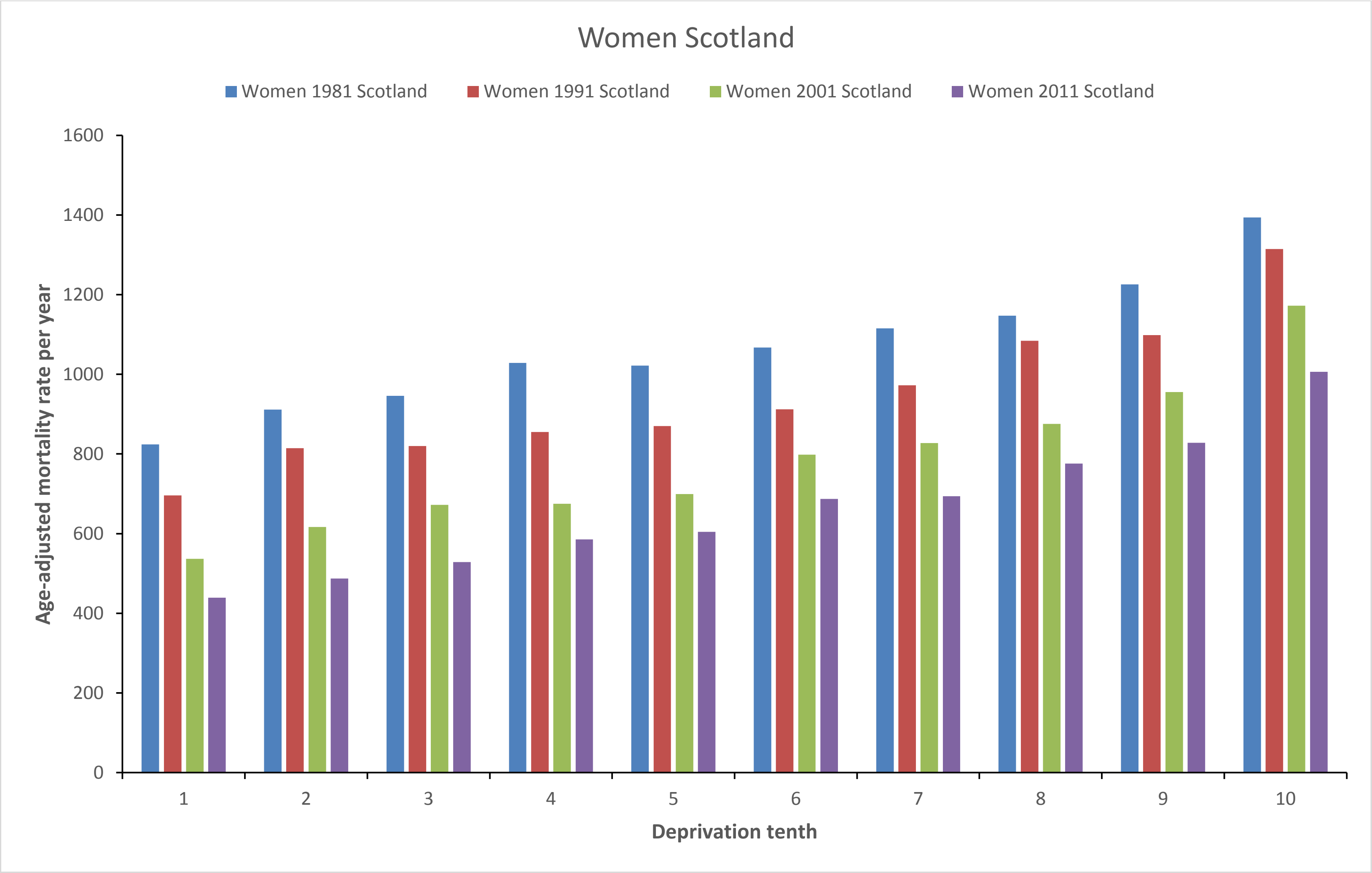
Women Scotland
Women 1981 Scotland
Women 1991 Scotland
Women 2001 Scotland
Women 2011 Scotland
Age-adjusted mortality rate per year
0
200
400
600
800
1000
1200
1400
1600
1
2
3
4
5
6
7
8
9
10
Deprivation tenth

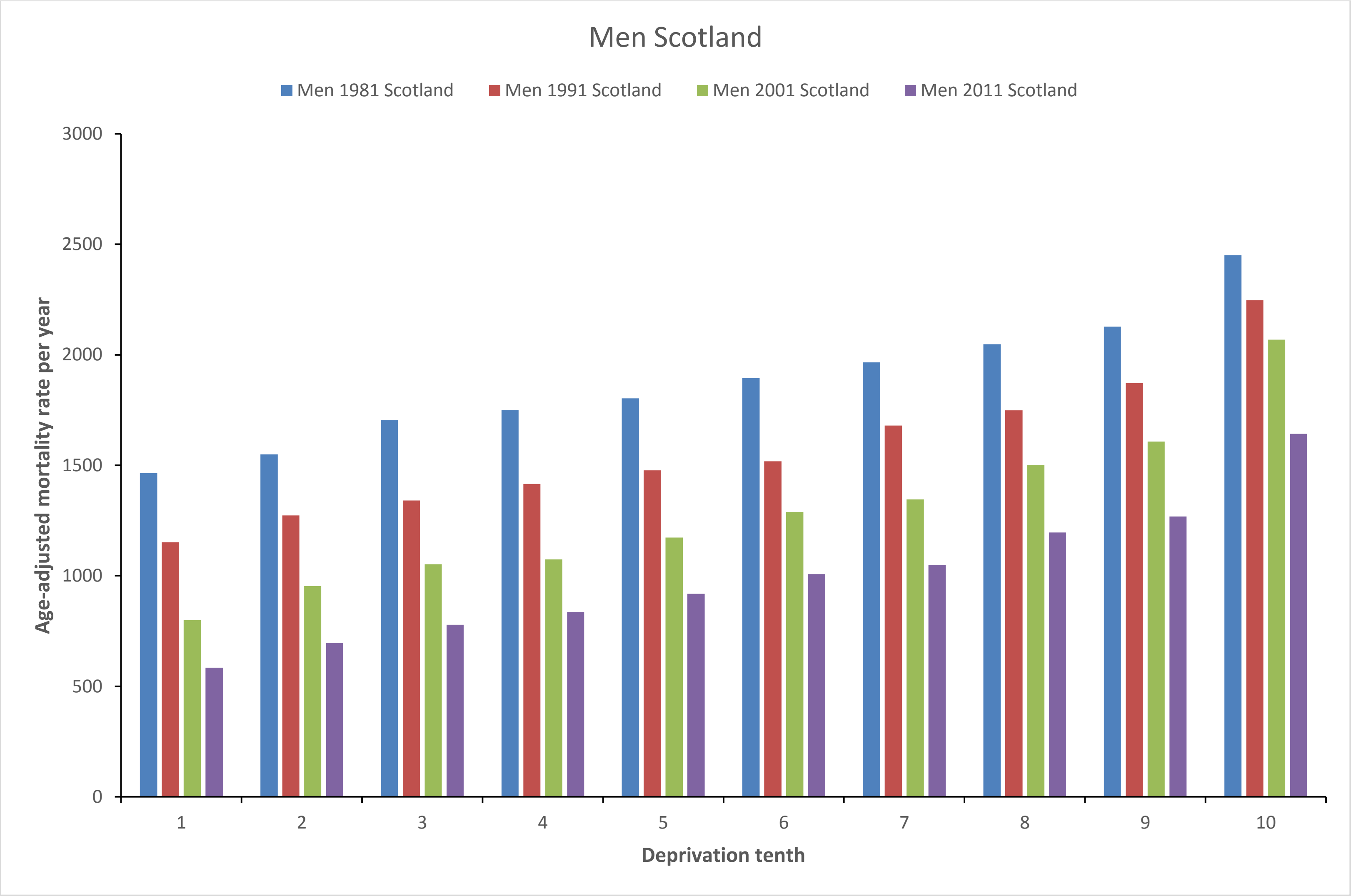
Men Scotland
Men 1981 Scotland
Men 1991 Scotland
Men 2001 Scotland
Men 2011 Scotland
Age-adjusted mortality rate per year
3000
2500
2000
1500
1000
500
0
1
2
3
4
5
6
7
8
9
10
Deprivation tenth

Relative Index of Inequality and Slope Index of Inequality

group	country	year	sex	SII	SII -lower ci	SI -upper CI	RII	RII -lower	RII _upper	SII per 100,000 per year		
E&W 1981 Men	E&W	1981	Men	0.007681	0.007525	0.007837645	1.6054	1.5913	1.619499	768.1188	752.5133	783.7645453
E&W 1991 Men	E&W	1991	Men	0.007477	0.007338	0.007616669	1.7599	1.7433	1.776685	747.7353	733.837	761.6668559
E&W 2001 Men	E&W	2001	Men	0.007581	0.007463	0.007699371	2.1199	2.098	2.142026	758.1331	746.35	769.9370795
E&W 2011 Men	E&W	2011	Men	0.006076	0.005981	0.006170969	2.2948	2.2686	2.321268	607.6128	598.1439	617.0969031
Scotland 1981 Men	Scotland	1981	Men	0.008873	0.008331	0.009418183	1.5937	1.5528	1.635678	887.267	833.1399	941.8182932
Scotland 1991 Men	Scotland	1991	Men	0.009623	0.00913	0.01011968	1.8457	1.7942	1.898683	962.3261	913.0035	1011.967998
Scotland 2001 Men	Scotland	2001	Men	0.010619	0.010193	0.011046661	2.3369	2.2673	2.408671	1061.894	1019.289	1104.666135
Scotland 2011 Men	Scotland	2011	Men	0.008786	0.008449	0.009123655	2.6362	2.5511	2.724258	878.5842	844.9002	912.3654795
E&W 1981 Women	E&W	1981	Women	0.003245	0.003145	0.003345687	1.4349	1.4202	1.449761	324.519	314.5077	334.5687
E&W 1991 Women	E&W	1991	Women	0.003561	0.003467	0.003656371	1.5807	1.5633	1.598297	356.1266	346.65	365.6371
E&W 2001 Women	E&W	2001	Women	0.003954	0.003868	0.004040811	1.8584	1.836	1.881023	395.4196	386.7829	404.0811
E&W 2011 Women	E&W	2011	Women	0.003622	0.003548	0.003695703	2.1035	2.0754	2.13204	362.1699	354.7871	369.5703
Scotland 1981 Women	Scotland	1981	Women	0.004713	0.004368	0.005062102	1.5436	1.4993	1.589246	471.3322	436.7923	506.2102
Scotland 1991 Women	Scotland	1991	Women	0.004971	0.00464	0.005305539	1.6877	1.6352	1.741814	497.1226	463.9918	530.5539
Scotland 2001 Women	Scotland	2001	Women	0.005301	0.005001	0.005602494	1.9778	1.9109	2.047052	530.0732	500.1122	560.2494
Scotland 2011 Women	Scotland	2011	Women	0.004999	0.004739	0.005259704	2.2371	2.1557	2.321455	499.8823	473.9428	525.9704

For peer review only

Interpolated 2008 for Scotland and 2007.5 for E&W figures		Linear interpolation	Log linear interpolation
E&W	Men	2.2336	2.231999
Scotland	Men	2.5464	2.542626
E&W	Women	2.0177	2.014243
Scotland	Women	2.1593	2.15591

Interpolated 1993 for Scotland and 1993.5 for E&W figures			
E&W	Men	1.8499	1.843737
Scotland	Men	1.944	1.934912
E&W	Women	1.6501	1.645948
Scotland	Women	1.7457	1.742076

For peer review only

Percentage change

% change over time periods		by Carstairs deprivation				By educational attainment	Mackenbach comparable figures are for 1991-5 to 2006-2010 for Scotland, and 1991-6 to 2006-9 for E&W Scotland midpoints: 1993 and 2008; E&W midpoints 1993.5 and 2007.5
**RII**		**1982-1991**	**1991-2001**	**1991-2011**	**1993-2008**	**1993-2008**	
E&W	Men	9.6%	1.4%		20.7%		20.7%
Scotland	Men	15.8%	10.3%		31.0%	-45.5%	31.0%
E&W	Women	10.2%			22.3%		22.3%
Scotland	Women	9.3%			23.7%	-20.0%	23.7%
**SII**							
E&W	Men	-2.7%			-12.0%		-12.0%
Scotland	Men	5.5%			-5.0%	-63.8%	-5.0%
E&W	Women	9.7%			2.1%		2.1%
Scotland	Women	5.5%			1.0%	-47.3%	1.0%

For peer review only

		Educational attainment			Carstairs deprivation		
		1991-1995	2006-2010	% change	1993	2008	% change
**RII**							
Scotland	Men	3.3	1.8	-45%	1.9	2.5	31%
Scotland	Women	2	1.6	-20%	1.7	2.2	24%
England & Wales	Men				1.8	2.5	38%
England & Wales	Women				1.6	2.0	23%
**Relative differences**							
Scotland	Men	1.81	1.83	1%			
Scotland	Women	1.57	1.75	11%			
England & Wales	Men	1.55	1.57	1%			
England & Wales	Women	1.46	1.46	0%			
**SII**							
Scotland	Men	1,634	591	-64%	982	934	-5%
Scotland	Women	601	317	-47%	504	509	1%
England & Wales	Men				750	660	-12%
England & Wales	Women				366	374	2%
**Absolute differences**							
Scotland	Men	681	502	-26%			
Scotland	Women	335	307	-8%			
England & Wales	Men	494	317	-36%			
England & Wales	Women	254	193	-24%			

For peer review only

STROBE 2007 (v4) Statement—Checklist of items that should be included in reports of *cross-sectional studies*

**Section/Topic**	**Item #**	**Recommendation**	**Reported on page #**
**Title and abstract**	1	(*a*) Indicate the study's design with a commonly used term in the title or the abstract	1
		(*b*) Provide in the abstract an informative and balanced summary of what was done and what was found	1
**Introduction**			
Background/rationale	2	Explain the scientific background and rationale for the investigation being reported	2-3
Objectives	3	State specific objectives, including any prespecified hypotheses	3
**Methods**			
Study design	4	Present key elements of study design early in the paper	4
Setting	5	Describe the setting, locations, and relevant dates, including periods of recruitment, exposure, follow-up, and data collection	4
Participants	6	(*a*) Give the eligibility criteria, and the sources and methods of selection of participants	4
Variables	7	Clearly define all outcomes, exposures, predictors, potential confounders, and effect modifiers. Give diagnostic criteria, if applicable	4
Data sources/ measurement	8*	For each variable of interest, give sources of data and details of methods of assessment (measurement). Describe comparability of assessment methods if there is more than one group	4
Bias	9	Describe any efforts to address potential sources of bias	4-5
Study size	10	Explain how the study size was arrived at	4
Quantitative variables	11	Explain how quantitative variables were handled in the analyses. If applicable, describe which groupings were chosen and why	4
Statistical methods	12	(*a*) Describe all statistical methods, including those used to control for confounding	4-5
		(*b*) Describe any methods used to examine subgroups and interactions	4-5
		(*c*) Explain how missing data were addressed	4-5
		(*d*) If applicable, describe analytical methods taking account of sampling strategy	n/a
		(*e*) Describe any sensitivity analyses	n/a
**Results**			

For peer review only

Participants	13*	(a) Report numbers of individuals at each stage of study—eg numbers potentially eligible, examined for eligibility, confirmed eligible, included in the study, completing follow-up, and analysed	Supplement
		(b) Give reasons for non-participation at each stage	n/a
		(c) Consider use of a flow diagram	n/a
Descriptive data	14*	(a) Give characteristics of study participants (eg demographic, clinical, social) and information on exposures and potential confounders	Supplement
		(b) Indicate number of participants with missing data for each variable of interest	n/a
Outcome data	15*	Report numbers of outcome events or summary measures	Supplement
Main results	16	(*a*) Give unadjusted estimates and, if applicable, confounder-adjusted estimates and their precision (eg, 95% confidence interval). Make clear which confounders were adjusted for and why they were included	5, 6 and supplement
		(*b*) Report category boundaries when continuous variables were categorized	n/a
		(*c*) If relevant, consider translating estimates of relative risk into absolute risk for a meaningful time period	n/a
Other analyses	17	Report other analyses done—eg analyses of subgroups and interactions, and sensitivity analyses	n/a
**Discussion**			
Key results	18	Summarise key results with reference to study objectives	7
Limitations	19	Discuss limitations of the study, taking into account sources of potential bias or imprecision. Discuss both direction and magnitude of any potential bias	8
Interpretation	20	Give a cautious overall interpretation of results considering objectives, limitations, multiplicity of analyses, results from similar studies, and other relevant evidence	8-9
Generalisability	21	Discuss the generalisability (external validity) of the study results	8-9
**Other information**			
Funding	22	Give the source of funding and the role of the funders for the present study and, if applicable, for the original study on which the present article is based	9

*Give information separately for cases and controls in case-control studies and, if applicable, for exposed and unexposed groups in cohort and cross-sectional studies.

Note: An Explanation and Elaboration article discusses each checklist item and gives methodological background and published examples of transparent reporting. The STROBE checklist is best used in conjunction with this article (freely available on the Web sites of PLoS Medicine at http://www.plosmedicine.org/, Annals of Internal Medicine at http://www.annals.org/, and Epidemiology at http://www.epidem.com/). Information on the STROBE Initiative is available at www.strobe-statement.org.

For peer review only

BMJ Open

How do trends in mortality inequalities by deprivation and education in Scotland and England & Wales compare? A repeat cross-sectional study.

Journal:	*BMJ Open*
Manuscript ID	bmjopen-2017-017590.R2
Article Type:	Research
Date Submitted by the Author:	09-Jun-2017

Complete List of Authors:	McCartney, Gerry; NHS Health Scotland<br>Popham, Frank; University of Glasgow, MRC/CSO Social and Public Health Sciences Unit<br>Katikireddi, Srinivasa; MRC/CSO Social & Public Health Sciences Unit, Evaluation of Social Interventions programme; NHS Lothian, Public Health and Health Policy<br>Walsh, David; Glasgow Centre for Population Health,<br>Schofield, Lauren; Information Services Division

**Primary Subject Heading**:	Public health
Secondary Subject Heading:	Epidemiology, Research methods
Keywords:	Health inequalities, Education, Deprivation, Scotland, England, Wales

For peer review only

SCHOLARONE™
Manuscripts

How do trends in mortality inequalities by deprivation and education in Scotland and England & Wales compare? A repeat cross-sectional study.

Gerry McCartney[1]*, Frank Popham[2], Srinivasa Vittal Katikireddi[2], David Walsh[3], Lauren Schofield[4]

[1] Public Health Science Directorate, NHS Health Scotland, Glasgow, Scotland.

[2] CSO/MRC Social and Public Health Sciences Unit, Institute of Health and Wellbeing, University of Glasgow, Glasgow, Scotland.

[3] Glasgow Centre for Population Health, Glasgow, Scotland.

[4] Public Health Intelligence, NHS National Services Scotland, Edinburgh, Scotland.

* Corresponding author. Gerry McCartney, Meridian Court, 5 Cadogan Street, Glasgow, gmccartney@nhs.net, 0141 201 2750.

Word count: 2,403 (excluding title page, abstract, summary box, references, figures, tables and notes).

For peer review only

Abstract

Objective

To compare the trends in mortality inequalities by educational attainment for with trends using area deprivation.

Setting

Scotland and England & Wales (E&W)

Participants

All people resident in Scotland and E&W between 1981 and 2011 aged 35-79 years.

Primary outcome measures

Absolute inequalities (measured using the Slope Index of Inequality (SII)) and relative inequalities (measured using the Relative Index of Inequality (RII)) in all-cause mortality.

Results

Relative inequalities in mortality by area deprivation have consistently increased for men and women in Scotland and E&W between 1981-3 and 2010-2. Absolute inequalities increased for men and women in Scotland, and for women in E&W, between 1981-3 and 2000-2 before subsequently falling. For men in E&W absolute inequalities were more stable until 2000-2 before a subsequent decline. Both absolute and relative inequalities were consistently higher in men and in Scotland. These trends contrast markedly with the reported declines in mortality inequalities by educational attainment and apparent improvement of Scotland's inequalities with those in E&W.

Conclusions

Trends in health inequalities differ when assessed using different measures of socioeconomic status, reflecting either genuinely variable trends in relation to different aspects of social stratification or varying error or bias. There are particular issues with the educational attainment data in Great Britain prior to 2001 that make these education-based estimates less certain.

Keywords

Health inequalities, education, deprivation, Scotland, England & Wales.

For peer review only - http://bmjopen.bmj.com/site/about/guidelines.xhtml

Strengths and limitations of the study

This study uses data on the whole population over time stratified into ten groups to calculate mortality inequalities and therefore avoids sampling bias and aggregation of heterogeneous groups.

Unlike educational attainment, the use of area deprivation as a measure of socio-economic position avoids difficulties relating to compositional change in the population over time.

Our area-based measure of socio-economic position applies a mean deprivation score to all people living within a postcode sector which will misclassify some individuals.

The measures used to calculate the Carstairs deprivation index are a pragmatic collection of indicators drawn from the census and do not fully capture the lived reality of deprivation and may be insensitive to changes in that experience over time.

Background

Health inequalities are systematic, avoidable and unjust differences in health outcomes[1] representing substantial unnecessary mortality and morbidity across countries.[2, 3] There has been a renewed policy focus on reducing health inequalities in recent years which has in turn led to debate about what should constitute success.[4]

Measures of population health tend to be seen as a relative phenomenon in that comparisons between populations are what help to contextualise whether a particular population health outcome is (relatively) good or bad. Health inequalities measures are similar in this way – it is how a health inequality measure for a population compares to those for other populations, including itself over time, that allows progress to be assessed and decisions made on priorities for action.[5] However, the health inequality measured for a single population can also be presented as either an absolute or relative difference. The interpretation of the trend in health inequalities within a country and differences in health inequality between countries can differ by whether assessed as absolute or relative difference.

Mackenbach et al. have argued from a pragmatic position that reduction in absolute difference should be considered a success because reductions in relative differences are more difficult to achieve when health is improving.[6, 7] However, relative measures of inequalities in mortality improved in both Great Britain and the USA using different measures prior to the changing political context in both countries from the 1980s, despite there being improvements in the mean health of the populations at this time.[8, 9] This suggests that it is possible and feasible to expect both absolute and relative measures of health inequalities to improve simultaneously given a conducive policy context.

In addition to relative and absolute measures of health inequality, there are also a variety of means of measuring socio-economic position (e.g. by income, social class, area deprivation, educational attainment) and many different health outcomes which are of interest (e.g. all-cause mortality, well-being, cause-specific morbidity, self-rated health). All of these options

For peer review only - http://bmjopen.bmj.com/site/about/guidelines.xhtml

have merit from a theoretical perspective and are likely to represent the operation of different social processes.[10, 11][12] Monitoring trends in health inequalities using relative and absolute measures, for a number of different social ranking and outcome measures, is therefore likely to be important to provide a comprehensive understanding of health inequality trends.[13] However, it is common for there to be either no available data for many measures or for the data to have substantial limitations, leading to pragmatic decisions being made on the preferred measures for monitoring health inequality trends within and between countries.

Recent observed declines in absolute measures of inequality for mortality rates between populations ranked by educational attainment between 1990 and 2010 in Europe have been interpreted as demonstrating substantial progress in achieving a reduction in health inequality.[7] However, these reductions need to be interpreted alongside other analyses because some of the data are not nationally representative (such as those drawn from cities), and because of problems with the educational attainment data upon which some of the analyses are based.[14, 15] Cautious interpretation of these data is necessary, as the priority given by policymakers to reducing health inequalities may be dependent on their understanding of these trends. Furthermore, the perceived success or failure of previous policy decisions may also be judged using these data – therefore, guiding future decision-making.

In order to assess whether the improvements in absolute inequalities reported by Mackenbach et al. for mortality inequalities by educational attainment[7] are also seen using other measures of social status, we compare the health inequalities trends for the same population strata (men and women aged 35-79 years) resident in Scotland and England & Wales using area deprivation. We then discuss the strengths and limitations of the different measures before reflecting on the implications for policy.

For peer review only

Methods

We report our methods here in line with the GATHER statement.[16]

Data

Individual mortality records were obtained from the National Records of Scotland (NRS) and the Office for National Statistics (ONS). Data were for residents of Scotland, and England & Wales only. Mortality records were linked to census wards (for England and Wales) and postcode sectors (for Scotland) of usual residence to allow allocation of area deprivation.

We extracted data on all deaths amongst those aged 35-79 years (to match the age strata used in Mackenbach et al.) in five year age bands, separately for men and women, for three years close to each census year (1981-1983[a], 1990-1992, 2000-2002, 2010-12) to provide more stable figures. The only exclusions were for those without a valid postcode (which tended to be for non-residents).

Socioeconomic exposure variable

[a] 1981-83 was used instead of 1980-82 because English deaths data were not available for 1980

For peer review only - http://bmjopen.bmj.com/site/about/guidelines.xhtml

The Carstairs deprivation score is derived from four Census variables calculated for small geographical areas: the proportion of economically active males seeking work; the proportion of people living in private households at a density of more than one person per room; the proportion of economically active males in occupational social class four or five on the Registrar General's categorisation; and the proportion of all persons in private households without access to a car or van.[17] Using the mean and standard deviations of this derived deprivation variable, postcode sectors (for Scotland, mean population 5,600 in 2011) and census wards (for England, mean population 6,540 in 2011) were ranked and divided into tenths (population weighted such that each decile had an equal population size) and each mortality record allocated a corresponding area deprivation score (1-10). The postcode sectors/wards and their deprivation scores were calculated according to the geographies and census data available at each time point. The deprivation scores for Scotland and E&W were calculated separately (i.e. the Scottish population was divided into ten equally sized units according to the deprivation score, and the E&W population was similarly divided into ten equally sized units).

Statistical analysis

We used the revised Census population estimates for each of the Census years published by NRS and the ONS as our denominator populations, multiplying the denominator by three to match the numerators (which were three years of deaths). As population undercounts were a feature of the 1981 and 1991 censuses,[18] census populations for both Scotland and England & Wales were constrained to the revised mid-year population estimates for these census years by age group and sex.

We standardised the mortality rates using the 1976 European standard population for each tenth of the population ranked by Carstairs deprivation, for comparability with Mackenbach et al.

We calculated mortality rates for each population, sex and time period using Poisson regression, in Stata, with population as the offset and used the margins command to standardise rates.[19]. The Relative Indices of Inequality (RIIs) were also obtained from Poisson models. For the RIIs, we calculated the mid-point of the cumulative distribution of people in each decile based on the mid-year population of 35-79 year olds for each country, sex and year combination. Following Mackenbach et al., Slope Indices of Inequality (SII) were calculated as 2*ASMR*(RII-1)/(RII+1) where ASMR = age standardised mortality rate for that country, sex, year combination. The SII confidence intervals (CIs) were calculated from the CIs for the RII and ASMR.

To compare with published mortality inequalities based on educational attainment, we interpolated the trends between Census years, using log-linear interpolation for the RIIs and linear interpolation for the SIIs, to obtain estimates for the same years for which Mackenbach et al. provide data (1993, 1993/4, 2007/8 and 2008). SII and RII estimates were published by Mackenbach using educational attainment data for Scotland but not E&W (as the latter were based on only two categories of educational attainment). The published time periods for the trends in inequalities (RII and SII for Scotland; rate ratio differences and absolute differences for Scotland and E&W) by educational attainment were 1991-1995 to 2006-2010 for Scotland, and 1991-1996 to 2006-2009 for E&W.[7]

For peer review only - http://bmjopen.bmj.com/site/about/guidelines.xhtml

Simple percentage changes for Scotland and E&W for men and women and for changes in RII and SII changes were then calculated using our Carstairs deprivation-based estimates and compared with the published estimates by Mackenbach et al.

Results

Mortality rates for those aged 35-79 years were consistently higher in the more deprived areas (for men and women, and in Scotland and E&W), with a linear relationship between deprivation and mortality (Supplement 1). Relative inequalities increased steadily between 1981-3 and 2010-12 in all groups, with RIIs consistently higher in men and in Scotland. The trends for absolute inequalities across the population were more mixed: for men and women in Scotland the SIIs increased between 1981-3 and 2000-2 before falling; whilst in E&W the trends were stable for men until 2000-2 before a subsequent decline, and increased for women until 2000-2 before a subsequent decline. The SII figures were again consistently higher for men and in Scotland (Table 1 and Figure 1).

Table 1 – Relative Indices of Inequality (RII) and Slope Indices of Inequality (SII) by Carstairs deprivation decile over time (men and women aged 35-79 years)

	Males		Females	
**Year**	**RII (95% CI)**	**SII per 100,000 per year (95% CI)**	**RII (95% CI)**	**SII per 100,000 per year (95% CI)**
**England & Wales**				
1981-3	1.61 (1.59 to 1.62)	768 (753 to 784)	1.43 (1.42 to 1.45)	325 (315 to 335)
1990-2	1.76 (1.74 to 1.78)	748 (734 to 762)	1.58 (1.56 to 1.60)	356 (347 to 366)
2000-2	2.12 (2.10 to 2.14)	758 (746 to 770)	1.86 (1.84 to 1.88)	395 (387 to 404)
2010-12	2.30 (2.27 to 2.32)	608 (598 to 617)	2.10 (2.08 to 2.13)	362 (355 to 370)
**Scotland**				
1981-3	1.59 (1.55 to 1.64)	887 (833 to 941)	1.54 (1.50 to 1.59)	471 (437 to 506)
1990-2	1.85 (1.79 to 1.90)	962 (913 to 1012)	1.69 (1.64 to 1.74)	497 (464 to 531)
2000-2	2.34 (2.27 to 2.41)	1,062 (1,019 to 1,105)	1.98 (1.91 to 2.05)	530 (500 to 560)
2010-12	2.64 (2.55 to 2.72)	879 (845 to 912)	2.24 (2.16 to 2.32)	500 (474 to 526)

These trends in mortality inequalities by Carstairs deprivation are markedly different from those reported by educational attainment. Between the early 1990s and late 2000s, the reported RIIs for mortality inequalities by educational attainment[7] declined in Scotland by 45% and 20% for men and women respectively. In contrast, the RIIs by Carstairs deprivation *increased* by 31% and 24% for men and women. The reported declines in educational attainment by SII for Scotland were even larger at 64% and 47% respectively,[7] but again these were markedly different from the observed stability amongst men (5% reduction) and

For peer review only - http://bmjopen.bmj.com/site/about/guidelines.xhtml

small increase of 1% for women using Carstairs deprivation for the same period (Figure 2, Table 2).

Table 2 – Changes in Relative Index of Inequality (RII), Slope Index of Inequality (SII), absolute and relative differences in mortality for those aged 35-79 years by educational attainment and Carstairs deprivation

		Educational attainment*			Carstairs deprivation		
		1991-1995	2006-2010	% change	1993	2008	% change
**RII**							
Scotland	Men	3.3	1.8	-45%	1.9	2.5	+31%
Scotland	Women	2	1.6	-20%	1.7	2.2	+24%
**SII (per 100,000 population per year)**							
Scotland	Men	1,634	591	-64%	982	934	-5%
Scotland	Women	601	317	-47%	504	509	+1%

* Data extracted from Mackenbach 2016.

There was no trend in RII or SII for E&W by educational attainment available for direct comparison, but the reported trends in relative differences were almost zero for educational attainment whereas our analyses showed increases of 38% and 23% for relative changes in Carstairs deprivation using the RII. The trends in absolute differences by educational attainment reported for E&W were -36% and -24% for men and women.[7] The equivalent changes in absolute inequalities (using the SII) for E&W using Carstairs deprivation were -12% for men and an increase of 2% for women (Supplement 1).

Discussion

Main results

Relative inequalities in mortality by area deprivation consistently increased for men and women in Scotland and E&W between 1981-3 and 2010-2. Absolute inequalities increased for men and women in Scotland, and for women in E&W, between 1981-3 and 2000-2 before subsequently falling. For men in E&W absolute inequalities were more stable until 2000-2 before a subsequent decline. Both absolute and relative inequalities were consistently higher in men and in Scotland. The increase in absolute inequalities between 1981 and 2001, and the increase in relative inequalities between 1981 and 2011 amongst men was much greater in Scotland than in E&W. The trends in inequalities amongst women were more similar, although consistently higher in Scotland.

A comparison of trends for inequalities by educational attainment with those by area deprivation show highly divergent patterns. Between the early 1990s and late 2000s, Mackenbach et al reported large decreases in absolute and relative inequalities in Scotland, whereas the trends by area deprivation showed large increases in relative inequalities and little change in absolute inequalities for the same period. Although the data for E&W could not be compared directly, the trends in relative and absolute inequalities using deprivation do not seem to be consistent with the trends using educational attainment.

For peer review only - http://bmjopen.bmj.com/site/about/guidelines.xhtml

Strengths and weaknesses

We have used data from the whole population based on area of residency to rank the population into ten approximately equal-sized groups to calculate RIIs and SIIs for four time points. In doing so, we have been able to include the distribution of mortality across the whole population (rather than just the extremes) and have been able to avoid aggregating heterogeneous populations into large groups. Area deprivation also has the advantage of avoiding compositional change over time, in that the population can be divided and ranked easily into ten groups at each time point, unlike educational attainment data which is subject to marked secular trends in attainment which impact differentially by age.[20] The deprivation-mortality relationship we describe in this paper is not subject to the lagged selection bias effects of some other approaches.[21]

As with all area-based measures of social ranking, the socioeconomic status applied to each individual is not a function of their personal socioeconomic status, but of the mean of all those living in that area. It therefore measures a different aspect of socioeconomic status and classifies some people who are individually socioeconomically disadvantaged as non-disadvantaged and vice-versa. The measures used to create the Carstairs deprivation index may also be becoming a less sensitive means of characterising socioeconomic position as car ownership becomes increasingly common and overcrowding of housing less common in the UK.[22-24] It is also possible that there is some reverse causation in the relationship between area deprivation and health status (particularly in relation to younger, healthier, individuals moving to less deprived areas [25, 26]). However, such population movement is unlikely to substantially undermine the results.[27, 28][29]

How this fits with the existing literature

The rise in health inequalities in Scotland and E&W and the causes of this are well described.[30] In particular, the rise in income inequalities and unemployment associated with the changed political context of the 1980s is likely to be a large part of any explanation.[22, 28, 31] It has been argued that the recent small declines in absolute inequalities for working age adults may be in part due to cohorts who were most adversely impacted by the application of neoliberalism and who were at high risk of death from alcohol- and drug-related deaths, suicide and violence, passing through the age of highest risk.[22, 32-34] In this way, there may be some lagged effects of political exposures during the 1980s and 1990s which contributed to the observed rise in health inequalities demonstrated here. Further exploration of how health inequalities have impacted on different age groups over time is merited.

The trends we describe for area deprivation here for E&W are very similar to those reported using the National Statistics Socio-Economic Classification (NS-SEC) (Webfigure 1) and for Scotland using another small-area measure of multiple deprivation.[35, 36] The similarity in the trends casts further doubt on the much more optimistic trends in inequalities described using educational attainment data to rank the population.[7]

Part of the reason for the markedly different trends may be the limited data available for educational attainment in Great Britain prior to 2001 which does not facilitate adequate stratification of the population to examine differences in outcomes.[14, 15, 37]. The secular trends in educational attainment, and the differences in these trends by age group, also make it important to examine narrow age groups in any cross-sectional analysis.[10]

For peer review only - http://bmjopen.bmj.com/site/about/guidelines.xhtml

Implications

Relative mortality inequalities using area deprivation in those aged 35-79 years has continuously increased since the early 1980s in Britain, and if there has been any decline in absolute inequalities since 2001 it has been small and limited to men. It is possible that there are different trends using different measures of socioeconomic status, or that one or more of the measures is subject to error or bias that has given rise to the divergent trends. There are particular issues with the educational attainment data in Great Britain prior to 2001 that make these estimates less certain. Further work to find adequate means to compare the extent of inequalities internationally should be undertaken.[38]

Figure 1 – Trends in SII and RII by Carstairs area deprivation (1982-2011, Scotland and England & Wales, men and women)

Figure 2 – Percentage change in SII and RII by Carstairs area deprivation and educational attainment (1993 to 2008, Scotland)

Notes

The Corresponding Author has the right to grant on behalf of all authors and does grant on behalf of all authors, an exclusive licence (or non exclusive for government employees) on a worldwide basis to the BMJ Publishing Group Ltd to permit this article (if accepted) to be published in BMJ editions and any other BMJPGL products and sublicences such use and exploit all subsidiary rights, as set out in our licence.

All authors have completed the Unified Competing Interest form (available on request from the corresponding author) and declare that no specific funding was received for this work. GM, DW and LS are employees of the National Health Service (NHS) in Scotland. FP and SVK are funded by the Medical Research Council and Chief Scientist's Office (MC_UU_12017/13 and SPHSU13), as part of the core funding for the MRC/CSO Social & Public Health Sciences Unit. In addition, SVK is funded by a NRS Senior Clinical Fellowship (SCAF/15/02).

We also declare that we have no financial relationships with any organisations that might have an interest in the submitted work in the previous three years and have no other relationships or activities that could appear to have influenced the submitted work.

We affirm that the manuscript is an honest, accurate, and transparent account of the study being reported; that no important aspects of the study have been omitted; and that any discrepancies from the study as planned (and, if relevant, registered) have been explained.

Ethical approval was not required for this study as the data were provided to us in aggregate form.

Data sharing – all of the available data are provided in the supplementary appendix.

For peer review only - http://bmjopen.bmj.com/site/about/guidelines.xhtml

GM, FP, VK and DW came up with the idea for this work. LS and FP did the data analysis. GM drafted the manuscript. All authors provided substantial edits and redrafting and approved the final draft.

References

1. Graham H. Understanding health inequalities. Maidenhead: Open University Press 2009.

2. Braveman PA, Kumanykia S, Fielding J, et al. Health Disparities and Health Equity: The Issue Is Justice. *American Journal of Public Health* 2011;101 (supplement 1):S149-55.

3. Woodward A, Kawachi I. Why reduce health inequalities?. *Journal of epidemiology and community health* 2000;54:923-9.

4. Closing the gap in a generation: Health equity through action on the social determinants of health. Final Report of the Commission on Social Determinants of Health 2008.

5. Popham F. Deprivation is a relative concept? Absolutely!. *Journal of epidemiology and community health* 2014;0:1-2.

6. Mackenbach JP. Should we aim to reduce relative or absolute inequalities in mortality?. *European Journal of Public Health* 2015;25:185 doi:10.1093/eurpub/cku217.

7. Mackenbach JP, Kulhanova I, Artnik B, et al. Changes in mortality inequalities over two decades: register based study of European countries. *BMJ* 2016;353 doi:doi: http://dx.doi.org/10.1136/bmj.i1732.

8. Thomas B, Dorling D, Davey Smith G. Inequalities in premature mortality in Britain: observational study from 1921 to 2007. *BMJ* 2010;341:c3639.

9. Krieger N, Rehkopf DH, Chen JT, et al. The fall and rise of US inequities in premature mortality: 1960-2002. *PLoS Medicine / Public Library of Science* 2008;5:e46.

10. Chen JT, Beckfield J, Waterman PD, et al. Can Changes in the Distributions of and Associations Between Education and Income Bias Temporal Comparisons of Health Disparities? An Exploration With Causal Graphs and Simulations. *American Journal of Epidemiology* 2013;177:870-81.

11. Geyer S, Hemström Ö, Peter R, et al. Education, income, and occupational class cannot be used interchangeably in social epidemiology. Empirical evidence against a common practice. *Journal of Epidemiology and Community Health* 2006;60:804-10 doi:10.1136/jech.2005.041319.

12. Wright EO. Understanding Class. London: Verso 2015.

13. Frank J, Haw S. Best Practice Guidelines for Monitoring Socioeconomic Inequalities in Health Status: Lessons from Scotland. *Millbank Quarterly* 2011;89:658-93.

For peer review only - http://bmjopen.bmj.com/site/about/guidelines.xhtml

14. McCartney G, Katikireddi SV, Walsh D. Interpreting the apparent absolute reduction in educational inequalities in mortality in much of Europe: a response to Mackenbach et al. *BMJ* 2016;353:i1732.

15. Flanagan L, McCartney G. How robust is the calculation of health inequality trends by educational attainment in England and Wales using the Longitudinal Study?. *Public health* 2015;129:621-8.

16. Stevens GA, Alkema L, Black RE, et al. Guidelines for Accurate and Transparent Health Estimates Reporting: the GATHER statement. *PLoS Medicine* 2016;13:e1002056 doi:10.1371/journal.pmed.1002056.

17. Carstairs V, Morris R. Deprivation and mortality: and alternative to social class?. *Community Medicine* 1989;11:210-9.

18. Dale A, Marsh C. The 1991 Census user's guide. London: HMSO 1993.

19. Muller CJ, MacLehose RF. Estimating predicted probabilities from logistic regression: different methods correspond to different target populations. *International Journal of Epidemiology* 2014;43:962-70.

20. Goujon A, S KC, Speringer M, et al. A harmonized dataset on global educational attainment between 1970 and 2060 - an analytical window into recent trends and future prospects in human capital development . *Journal of Demographic Economics* 2016;82:315-63 doi:https://doi.org/10.1017/dem.2016.10.

21. Dowd JB, Hamoudi A. Is life expectancy really falling for groups of low socio-economic status? Lagged selection bias and artefactual trends in mortality. *International Journal of Epidemiology* 2014;43:983-8 doi:10.1093/ije/dyu120.

22. Walsh D, McCartney G, Collins C, et al. History, politics and vulnerability: explaining excess mortality 2016.

23. Schofield L, Walsh D, Munoz-Arroyo R, et al. Dying younger in Scotland: Trends in mortality and deprivation relative to England and Wales, 1981–2011. *Health and Place* 2016;40:106-15.

24. Allik M, Brown D, Dundas R, et al. Developing a new small-area measure of deprivation using 2001 and 2011 census data from Scotland. *Health Place* 2016;39:122-30 doi:http://dx.doi.org/10.1016/j.healthplace.2016.03.006.

25. Norman P, Boyle P. Are health inequalities between differently deprived areas evident at different ages? A longitudinal study of census records in England and Wales, 1991–2001. Health Place 2014;26:88-93 doi:https://doi.org/10.1016/j.healthplace.2013.12.010.

26. Norman P, Boyle P, Rees P. Selective migration, health and deprivation: a longitudinal analysis. Soc Sci Med 2005;60:2755-71 doi:https://doi.org/10.1016/j.socscimed.2004.11.008.

For peer review only - http://bmjopen.bmj.com/site/about/guidelines.xhtml

27. Katikireddi SV, Whitley E, Lewsey J, et al. Socioeconomic status as an effect modifier of alcohol consumption and harm: analysis of linked cohort data*. Lancet Public Health* 2017 doi:http://dx.doi.org/10.1016/S2468-2667(17)30078-6.

28. McCartney G, Collins C, Mackenzie M. What (or who) causes health inequalities: Theories, evidence and implications?*. Health Policy* 2013;113:221-7.

29. Popham F, Boyle P, O'Reilly D, et al. Selective internal migration. Does it explain Glasgow's worsening mortality record? Scottish Longitudinal Study (SLS) Research Paper Series. Research working paper 5 2009. 30. Marmot M, Allen J, Goldblatt P, et al. Fair Society, Healthy Lives. The Marmot review 2010.

31. Beeston C, McCartney G, Ford J, et al. Health Inequalities Policy review for the Scottish Ministerial Task Force on Health Inequalities 2013.

32. McCartney G, Bouttell J, Craig N, et al. Explaining trends in alcohol-related harms in Scotland 1991-2011 (II): policy, social norms, the alcohol market, clinical changes and a synthesis*. Public health* 2016;132:24-32.

33. McCartney G, Bouttell J, Craig N, et al. Explaining trends in alcohol-related harms in Scotland, 1991-2011 (I): the role of incomes, effects of socio-economic and political adversity and demographic change 2016;132:13-23.

34. Parkinson J, Minton J, Lewsey j, et al. Recent cohort effects in suicide in Scotland: a legacy of the 1980s? *. Journal of Epidemiology and Community Health* 2016 doi:10.1136/jech-2016-207296.

35. Trend in life expectancy at birth and at age 65 by socio-economic position based on the National Statistics Socio-economic Classification, England and Wales: 1982—1986 to 2007—2011 2015.

36. Long-Term Monitoring of Health Inequalities 2015. Edinburgh, Scottish Government, 2016.

37. Schneider SL. The application of the ISCED-97 to the UK's educational qualifications. In: Schneider SL, ed. The International Standard Classification of Education: an Evaluation of Content and Criterion Validity for 15 European Countries. Mannheim: MZES 2008.

38. Popham F, Dibben C, Bambra C. Are health inequalities really not the smallest in the Nordic welfare states? A comparison of mortality inequality in 37 countries*. Journal of Epidemiology & Community Health* 2013; 67:412-8 doi:10.1136/jech-2012-201525.

For peer review only - http://bmjopen.bmj.com/site/about/guidelines.xhtml

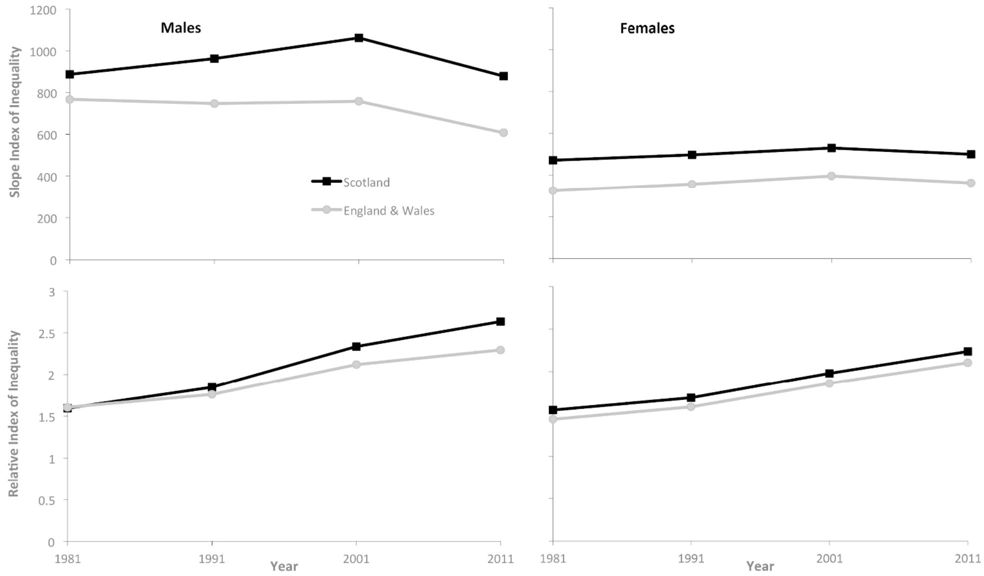

Figure 1 – Trends in SII and RII by Carstairs area deprivation (1982-2011, Scotland and England & Wales, men and women

239x139mm (300 x 300 DPI)

review only

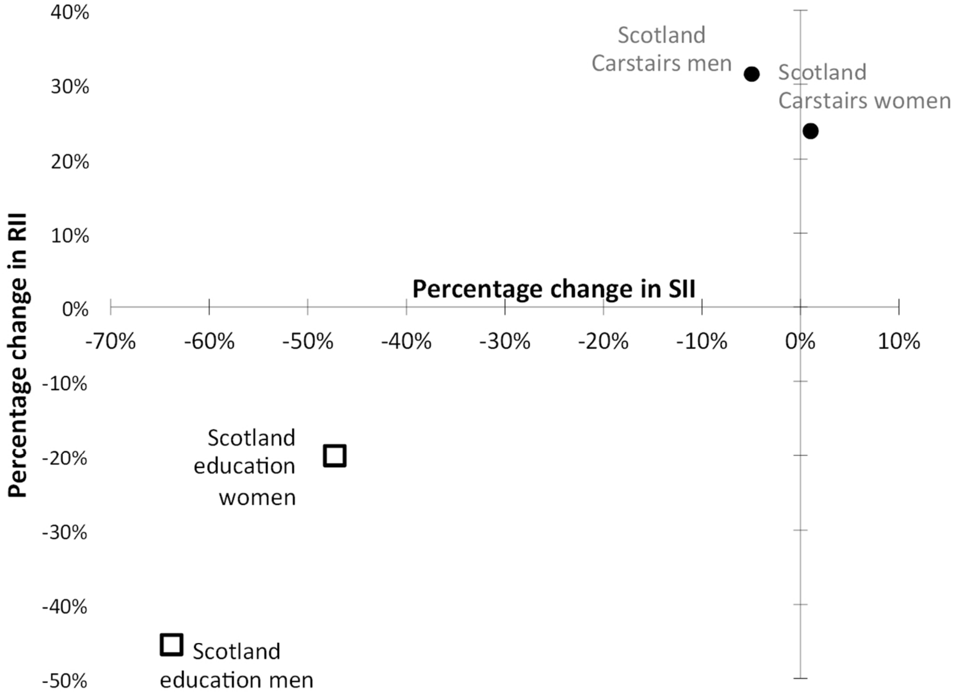

Figure 2 – Percentage change in SII and RII by Carstairs area deprivation and educational attainment (1993 to 2008, Scotland)

125x94mm (300 x 300 DPI)

For peer review only - http://bmjopen.bmj.com/site/about/guidelines.xhtml

Crude and age-adjusted rates

group	country	sex	year	decile	crude_rate	age_adj_rate	age_adj_rate_lower_CI	age_adj_rate_upper_CI	Age-adjusted mortality per 100,000 per year
E&W 1981 Women	E&W	Women	1981	1	0.009612889	0.007755622	0.007672978	0.007838266	775.5622
E&W 1981 Women	E&W	Women	1981	2	0.010476029	0.007906884	0.007825425	0.007988344	790.6884
E&W 1981 Women	E&W	Women	1981	3	0.011498301	0.008332084	0.008249331	0.008414837	833.2084
E&W 1981 Women	E&W	Women	1981	4	0.011785442	0.008367835	0.008285735	0.008449935	836.7835
E&W 1981 Women	E&W	Women	1981	5	0.012349643	0.008772567	0.008687877	0.008857258	877.2567
E&W 1981 Women	E&W	Women	1981	6	0.012604976	0.008939335	0.008853824	0.009024845	893.9335
E&W 1981 Women	E&W	Women	1981	7	0.013215572	0.009388286	0.009299824	0.009476748	938.8286
E&W 1981 Women	E&W	Women	1981	8	0.013763275	0.009750938	0.00966083	0.009841046	975.0938
E&W 1981 Women	E&W	Women	1981	9	0.014304536	0.01028942	0.010195368	0.010383472	1028.942
E&W 1981 Women	E&W	Women	1981	10	0.015758992	0.01145973	0.011357135	0.011562326	1145.973
E&W 1991 Women	E&W	Women	1991	1	0.007720282	0.006422884	0.006349762	0.006496005	642.2884
E&W 1991 Women	E&W	Women	1991	2	0.008475934	0.006741511	0.006666873	0.00681615	674.1511
E&W 1991 Women	E&W	Women	1991	3	0.009114578	0.006902071	0.006827492	0.006976651	690.2071
E&W 1991 Women	E&W	Women	1991	4	0.009720711	0.00722439	0.007147987	0.007300793	722.439
E&W 1991 Women	E&W	Women	1991	5	0.010306351	0.007550758	0.007472328	0.007629189	755.0758
E&W 1991 Women	E&W	Women	1991	6	0.010769808	0.007825069	0.007744471	0.007905667	782.5069
E&W 1991 Women	E&W	Women	1991	7	0.011524274	0.008384602	0.008300254	0.00846895	838.4602
E&W 1991 Women	E&W	Women	1991	8	0.012323026	0.008901691	0.008813651	0.00898973	890.1691
E&W 1991 Women	E&W	Women	1991	9	0.012662662	0.009270408	0.009179103	0.009361713	927.0408
E&W 1991 Women	E&W	Women	1991	10	0.013763391	0.010391625	0.010289922	0.010493329	1039.163
E&W 2001 Women	E&W	Women	2001	1	0.00579012	0.004941082	0.004879425	0.005002738	494.1082
E&W 2001 Women	E&W	Women	2001	2	0.00664026	0.005331322	0.005268285	0.005394358	533.1322
E&W 2001 Women	E&W	Women	2001	3	0.007079005	0.005542715	0.005478404	0.005607025	554.2715
E&W 2001 Women	E&W	Women	2001	4	0.007456318	0.005833322	0.005766699	0.005899944	583.3322
E&W 2001 Women	E&W	Women	2001	5	0.008136969	0.00624155	0.006172043	0.006311057	624.155
E&W 2001 Women	E&W	Women	2001	6	0.008767891	0.006741833	0.006668222	0.006815443	674.1833
E&W 2001 Women	E&W	Women	2001	7	0.009152499	0.007223277	0.007145289	0.007301265	722.3277
E&W 2001 Women	E&W	Women	2001	8	0.009599881	0.007714746	0.007632123	0.00779737	771.4746
E&W 2001 Women	E&W	Women	2001	9	0.010201663	0.008228177	0.008141838	0.008314516	822.8177
E&W 2001 Women	E&W	Women	2001	10	0.010587698	0.009121022	0.009022989	0.009219056	912.1022

For peer review only

E&W 2011 Women	E&W	Women	2011	1	0.00452512	0.003655192	0.003606717	0.003703668	365.5192
E&W 2011 Women	E&W	Women	2011	2	0.005046366	0.004001625	0.003950601	0.004052648	400.1625
E&W 2011 Women	E&W	Women	2011	3	0.005369199	0.004198358	0.004145849	0.004250867	419.8358
E&W 2011 Women	E&W	Women	2011	4	0.005613362	0.004515388	0.004459541	0.004571235	451.5388
E&W 2011 Women	E&W	Women	2011	5	0.006173427	0.004944128	0.004884818	0.005003437	494.4128
E&W 2011 Women	E&W	Women	2011	6	0.00644843	0.005288613	0.005225253	0.005351974	528.8613
E&W 2011 Women	E&W	Women	2011	7	0.006972373	0.005895116	0.005826416	0.005963816	589.5116
E&W 2011 Women	E&W	Women	2011	8	0.007102966	0.006229234	0.006155617	0.006302851	622.9234
E&W 2011 Women	E&W	Women	2011	9	0.00742091	0.006763139	0.006682834	0.006843444	676.3139
E&W 2011 Women	E&W	Women	2011	10	0.007504293	0.007255963	0.007166445	0.007345481	725.5963
E&W 1981 Men	E&W	Men	1981	1	0.013836215	0.013282844	0.01316358	0.013402108	1328.284
E&W 1981 Men	E&W	Men	1981	2	0.015771348	0.014010972	0.013892168	0.014129777	1401.097
E&W 1981 Men	E&W	Men	1981	3	0.01732869	0.014876871	0.014755229	0.014998513	1487.687
E&W 1981 Men	E&W	Men	1981	4	0.017783471	0.014983149	0.014861826	0.015104471	1498.315
E&W 1981 Men	E&W	Men	1981	5	0.018750443	0.01581156	0.015685979	0.015937141	1581.156
E&W 1981 Men	E&W	Men	1981	6	0.019528808	0.016484809	0.016356086	0.016613532	1648.481
E&W 1981 Men	E&W	Men	1981	7	0.020527071	0.017374747	0.017241317	0.017508177	1737.475
E&W 1981 Men	E&W	Men	1981	8	0.021516646	0.018263609	0.018126787	0.018400431	1826.361
E&W 1981 Men	E&W	Men	1981	9	0.022342745	0.019196938	0.019054611	0.019339266	1919.694
E&W 1981 Men	E&W	Men	1981	10	0.024349277	0.021351602	0.021197785	0.02150542	2135.16
E&W 1991 Men	E&W	Men	1991	1	0.01103878	0.010488629	0.010388499	0.010588758	1048.863
E&W 1991 Men	E&W	Men	1991	2	0.01226422	0.011254249	0.011150527	0.011357971	1125.425
E&W 1991 Men	E&W	Men	1991	3	0.01325232	0.01165966	0.011554955	0.011764365	1165.966
E&W 1991 Men	E&W	Men	1991	4	0.014156857	0.012279371	0.012171604	0.012387137	1227.937
E&W 1991 Men	E&W	Men	1991	5	0.014950535	0.012817366	0.012706626	0.012928105	1281.737
E&W 1991 Men	E&W	Men	1991	6	0.015800782	0.013509515	0.013394194	0.013624835	1350.951
E&W 1991 Men	E&W	Men	1991	7	0.017159268	0.014640691	0.014519764	0.014761619	1464.069
E&W 1991 Men	E&W	Men	1991	8	0.018230566	0.015567663	0.015441112	0.015694214	1556.766
E&W 1991 Men	E&W	Men	1991	9	0.019024402	0.016314241	0.016183029	0.016445452	1631.424
E&W 1991 Men	E&W	Men	1991	10	0.021058194	0.018420098	0.018274859	0.018565338	1842.01
E&W 2001 Men	E&W	Men	2001	1	0.008202026	0.007523487	0.007444123	0.007602851	752.3487
E&W 2001 Men	E&W	Men	2001	2	0.009261247	0.008104411	0.00802296	0.008185863	810.4411

For peer review only

E&W 2001 Men	E&W	Men	2001	3	0.010043937	0.008723544	0.008638119	0.008808969	872.3544
E&W 2001 Men	E&W	Men	2001	4	0.010621459	0.009262298	0.009173214	0.009351383	926.2298
E&W 2001 Men	E&W	Men	2001	5	0.011491073	0.009944548	0.009851023	0.010038073	994.4548
E&W 2001 Men	E&W	Men	2001	6	0.01228122	0.010788395	0.010688931	0.010887858	1078.839
E&W 2001 Men	E&W	Men	2001	7	0.01297947	0.011653791	0.011547806	0.011759775	1165.379
E&W 2001 Men	E&W	Men	2001	8	0.013801846	0.01256548	0.012453197	0.012677762	1256.548
E&W 2001 Men	E&W	Men	2001	9	0.015039413	0.013770089	0.013651084	0.013889093	1377.009
E&W 2001 Men	E&W	Men	2001	10	0.016328106	0.015628966	0.015493243	0.01576469	1562.897
E&W 2011 Men	E&W	Men	2011	1	0.006531269	0.005324398	0.005264408	0.005384389	532.4398
E&W 2011 Men	E&W	Men	2011	2	0.007209251	0.005844454	0.005780896	0.005908012	584.4454
E&W 2011 Men	E&W	Men	2011	3	0.00771371	0.006289675	0.006222895	0.006356455	628.9675
E&W 2011 Men	E&W	Men	2011	4	0.008046375	0.006782732	0.006711645	0.00685382	678.2732
E&W 2011 Men	E&W	Men	2011	5	0.008744033	0.007441684	0.00736579	0.007517578	744.1684
E&W 2011 Men	E&W	Men	2011	6	0.009159381	0.008064742	0.007983004	0.00814648	806.4742
E&W 2011 Men	E&W	Men	2011	7	0.009774983	0.008875782	0.008787907	0.008963658	887.5782
E&W 2011 Men	E&W	Men	2011	8	0.010086383	0.009604045	0.009508757	0.009699333	960.4045
E&W 2011 Men	E&W	Men	2011	9	0.010646058	0.010524807	0.010420662	0.010628953	1052.481
E&W 2011 Men	E&W	Men	2011	10	0.011066971	0.011640176	0.011522596	0.011757755	1164.018
Scotland 1981 Women	Scotland	Women	1981	1	0.010525108	0.008241884	0.007898118	0.008585651	824.1884
Scotland 1981 Women	Scotland	Women	1981	2	0.013282962	0.00911192	0.008803342	0.009420497	911.192
Scotland 1981 Women	Scotland	Women	1981	3	0.013353083	0.009459393	0.009169682	0.009749104	945.9393
Scotland 1981 Women	Scotland	Women	1981	4	0.014387775	0.01028801	0.009965086	0.010610935	1028.801
Scotland 1981 Women	Scotland	Women	1981	5	0.014238784	0.010217297	0.009906958	0.010527636	1021.73
Scotland 1981 Women	Scotland	Women	1981	6	0.014408361	0.01067589	0.010387509	0.010964272	1067.589
Scotland 1981 Women	Scotland	Women	1981	7	0.014307882	0.011156961	0.010869841	0.011444081	1115.696
Scotland 1981 Women	Scotland	Women	1981	8	0.015429545	0.011470359	0.011197754	0.011742964	1147.036
Scotland 1981 Women	Scotland	Women	1981	9	0.015946955	0.012255514	0.011984849	0.012526179	1225.551
Scotland 1981 Women	Scotland	Women	1981	10	0.017832188	0.01393502	0.013631905	0.014238135	1393.502
Scotland 1991 Women	Scotland	Women	1991	1	0.007778107	0.006961045	0.006652506	0.007269583	696.1045
Scotland 1991 Women	Scotland	Women	1991	2	0.010644362	0.008145254	0.00784715	0.008443357	814.5254
Scotland 1991 Women	Scotland	Women	1991	3	0.010573639	0.008200427	0.007914969	0.008485886	820.0427
Scotland 1991 Women	Scotland	Women	1991	4	0.011319426	0.008554462	0.008286397	0.008822528	855.4462

For peer review only

Scotland 1991 Women	Scotland	Women	1991	5	0.011419762	0.008703245	0.008439098	0.008967393	870.3245
Scotland 1991 Women	Scotland	Women	1991	6	0.011959812	0.00912001	0.008847431	0.009392588	912.001
Scotland 1991 Women	Scotland	Women	1991	7	0.01254952	0.009723669	0.009461309	0.009986029	972.3669
Scotland 1991 Women	Scotland	Women	1991	8	0.014283347	0.010841657	0.010559872	0.011123442	1084.166
Scotland 1991 Women	Scotland	Women	1991	9	0.014621061	0.010985495	0.010716051	0.011254939	1098.55
Scotland 1991 Women	Scotland	Women	1991	10	0.017393045	0.013147151	0.012832815	0.013461487	1314.715
Scotland 2001 Women	Scotland	Women	2001	1	0.006054715	0.005369293	0.005119773	0.005618813	536.9293
Scotland 2001 Women	Scotland	Women	2001	2	0.007473842	0.006167007	0.005932055	0.006401959	616.7007
Scotland 2001 Women	Scotland	Women	2001	3	0.007940882	0.006723365	0.006462115	0.006984616	672.3365
Scotland 2001 Women	Scotland	Women	2001	4	0.008454126	0.006752374	0.00650612	0.006998627	675.2374
Scotland 2001 Women	Scotland	Women	2001	5	0.008833125	0.006991448	0.006751617	0.007231278	699.1448
Scotland 2001 Women	Scotland	Women	2001	6	0.009997091	0.007983593	0.007739673	0.008227513	798.3593
Scotland 2001 Women	Scotland	Women	2001	7	0.01012218	0.008270903	0.008026704	0.008515101	827.0903
Scotland 2001 Women	Scotland	Women	2001	8	0.011031647	0.008757081	0.00851662	0.008997541	875.7081
Scotland 2001 Women	Scotland	Women	2001	9	0.012063884	0.009551886	0.009302581	0.00980119	955.1886
Scotland 2001 Women	Scotland	Women	2001	10	0.014525514	0.011720127	0.011420843	0.01201941	1172.013
Scotland 2011 Women	Scotland	Women	2011	1	0.005188653	0.004390383	0.00421379	0.004566976	439.0383
Scotland 2011 Women	Scotland	Women	2011	2	0.005999477	0.004874167	0.004690536	0.005057797	487.4167
Scotland 2011 Women	Scotland	Women	2011	3	0.006649881	0.005285443	0.005095168	0.005475718	528.5443
Scotland 2011 Women	Scotland	Women	2011	4	0.007424119	0.005853295	0.005651062	0.006055528	585.3295
Scotland 2011 Women	Scotland	Women	2011	5	0.007461833	0.006041823	0.005833749	0.006249897	604.1823
Scotland 2011 Women	Scotland	Women	2011	6	0.00826023	0.006869481	0.006642285	0.007096677	686.9481
Scotland 2011 Women	Scotland	Women	2011	7	0.008382318	0.006941867	0.006709153	0.007174581	694.1867
Scotland 2011 Women	Scotland	Women	2011	8	0.00930957	0.007758332	0.00750849	0.008008174	775.8332
Scotland 2011 Women	Scotland	Women	2011	9	0.010235269	0.008278501	0.008024174	0.008532828	827.8501
Scotland 2011 Women	Scotland	Women	2011	10	0.01181909	0.010062415	0.009766534	0.010358295	1006.241
Scotland 1981 Men	Scotland	Men	1981	1	0.014829473	0.014645599	0.014114115	0.015177084	1464.56
Scotland 1981 Men	Scotland	Men	1981	2	0.018356204	0.01548973	0.015027761	0.0159517	1548.973
Scotland 1981 Men	Scotland	Men	1981	3	0.019749633	0.017033235	0.016591014	0.017475456	1703.323
Scotland 1981 Men	Scotland	Men	1981	4	0.020395399	0.0174934	0.017016793	0.017970006	1749.34
Scotland 1981 Men	Scotland	Men	1981	5	0.020899901	0.018031108	0.017562668	0.018499549	1803.111
Scotland 1981 Men	Scotland	Men	1981	6	0.021253547	0.018943333	0.018504819	0.019381847	1894.333

For peer review only

Scotland 1981 Men	Scotland	Men	1981	7	0.021440803	0.019655468	0.019223348	0.020087587	1965.547
Scotland 1981 Men	Scotland	Men	1981	8	0.023382957	0.020479833	0.020064179	0.020895487	2047.983
Scotland 1981 Men	Scotland	Men	1981	9	0.023369206	0.021274203	0.020864997	0.021683409	2127.42
Scotland 1981 Men	Scotland	Men	1981	10	0.026627273	0.02451257	0.024055239	0.0249699	2451.257
Scotland 1991 Men	Scotland	Men	1991	1	0.010887721	0.011516785	0.011076989	0.011956582	1151.679
Scotland 1991 Men	Scotland	Men	1991	2	0.01369037	0.012734436	0.012317722	0.01315115	1273.444
Scotland 1991 Men	Scotland	Men	1991	3	0.014336067	0.013406395	0.013000268	0.013812523	1340.64
Scotland 1991 Men	Scotland	Men	1991	4	0.015868404	0.014157409	0.013775834	0.014538983	1415.741
Scotland 1991 Men	Scotland	Men	1991	5	0.016307012	0.014769597	0.014385004	0.01515419	1476.96
Scotland 1991 Men	Scotland	Men	1991	6	0.016837016	0.015184471	0.014790523	0.015578419	1518.447
Scotland 1991 Men	Scotland	Men	1991	7	0.018238995	0.016798337	0.016411561	0.017185113	1679.834
Scotland 1991 Men	Scotland	Men	1991	8	0.019793804	0.01748933	0.017087907	0.017890753	1748.933
Scotland 1991 Men	Scotland	Men	1991	9	0.021303564	0.018721615	0.018327746	0.019115484	1872.161
Scotland 1991 Men	Scotland	Men	1991	10	0.025642796	0.022471745	0.02201256	0.02293093	2247.174
Scotland 2001 Men	Scotland	Men	2001	1	0.008092963	0.007992258	0.007663932	0.008320584	799.2258
Scotland 2001 Men	Scotland	Men	2001	2	0.009987633	0.009536619	0.009219267	0.009853972	953.6619
Scotland 2001 Men	Scotland	Men	2001	3	0.011075126	0.010522013	0.010168719	0.010875308	1052.201
Scotland 2001 Men	Scotland	Men	2001	4	0.011944081	0.01074405	0.010409199	0.011078901	1074.405
Scotland 2001 Men	Scotland	Men	2001	5	0.012804428	0.011731175	0.011391259	0.012071091	1173.117
Scotland 2001 Men	Scotland	Men	2001	6	0.01411566	0.012887867	0.012549888	0.013225846	1288.787
Scotland 2001 Men	Scotland	Men	2001	7	0.01443252	0.013449304	0.013108893	0.013789715	1344.93
Scotland 2001 Men	Scotland	Men	2001	8	0.016276902	0.015007962	0.014660355	0.015355569	1500.796
Scotland 2001 Men	Scotland	Men	2001	9	0.017687748	0.016079247	0.015721943	0.01643655	1607.925
Scotland 2001 Men	Scotland	Men	2001	10	0.022427071	0.020676718	0.02023799	0.021115446	2067.672
Scotland 2011 Men	Scotland	Men	2011	1	0.006641684	0.005838869	0.00562563	0.006052108	583.8869
Scotland 2011 Men	Scotland	Men	2011	2	0.008213557	0.006959545	0.00673004	0.007189049	695.9545
Scotland 2011 Men	Scotland	Men	2011	3	0.009326207	0.007787182	0.007543687	0.008030678	778.7182
Scotland 2011 Men	Scotland	Men	2011	4	0.009918607	0.008362189	0.008105543	0.008618836	836.2189
Scotland 2011 Men	Scotland	Men	2011	5	0.010430598	0.009189839	0.008916447	0.00946323	918.9839
Scotland 2011 Men	Scotland	Men	2011	6	0.01124958	0.010073004	0.009780454	0.010365554	1007.3
Scotland 2011 Men	Scotland	Men	2011	7	0.011551229	0.010486904	0.010182382	0.010791425	1048.69
Scotland 2011 Men	Scotland	Men	2011	8	0.012963478	0.011963323	0.011630806	0.012295839	1196.332

For peer review only

Scotland 2011 Men	Scotland	Men	2011	9	0.014115218	0.01268553	0.01234699	0.01302407	1268.553
Scotland 2011 Men	Scotland	Men	2011	10	0.017255414	0.01641974	0.016013286	0.016826194	1641.974

For peer review only

Descriptive graphs – age-adjusted mortality rates per 100,000 per year by sex, deprivation tenth and country

For peer review only

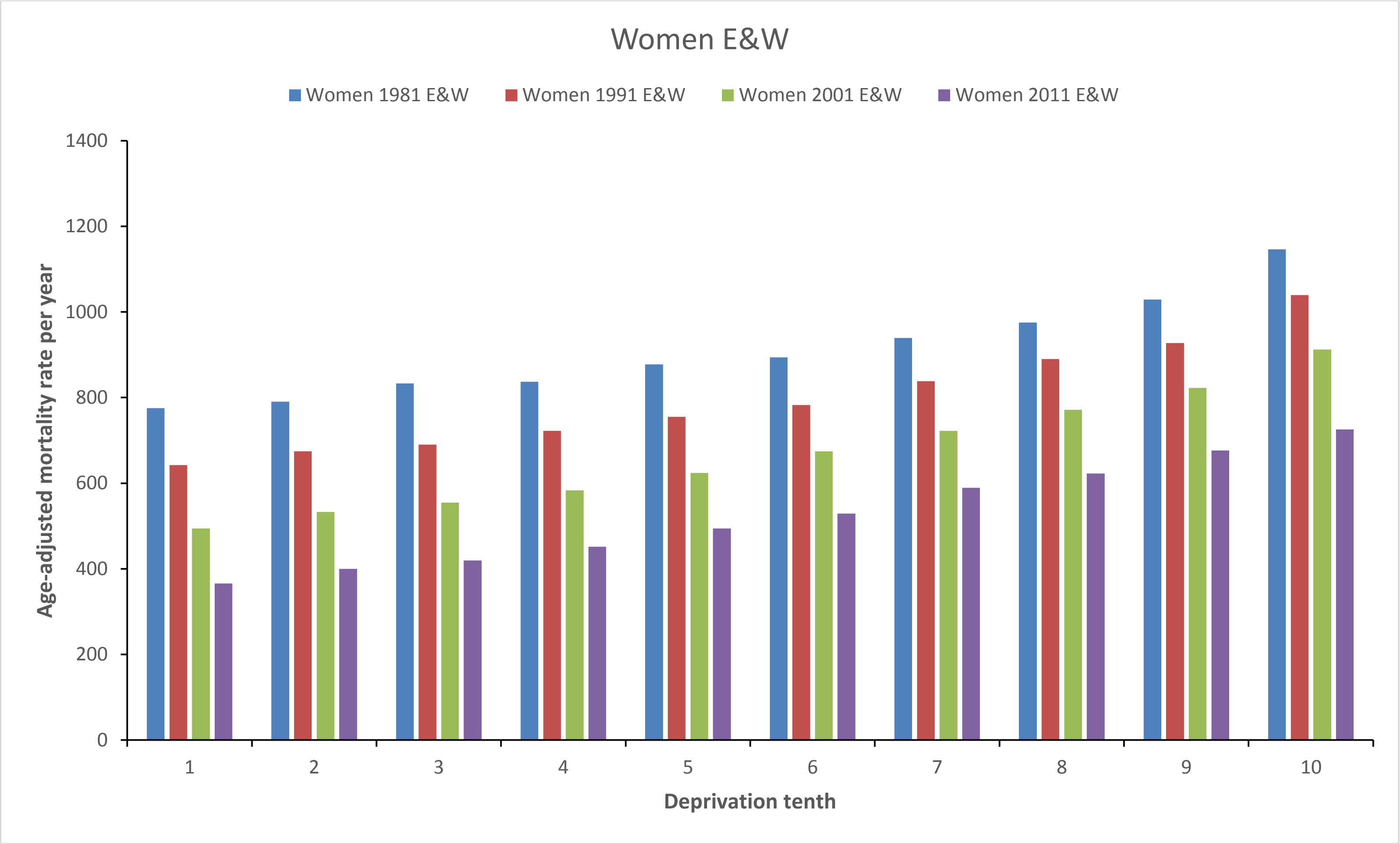
Women E&W
Women 1981 E&W
Women 1991 E&W
Women 2001 E&W
Women 2011 E&W
Age-adjusted mortality rate per year
1400
1200
1000
800
600
400
200
0
1
2
3
4
5
6
7
8
9
10
Deprivation tenth

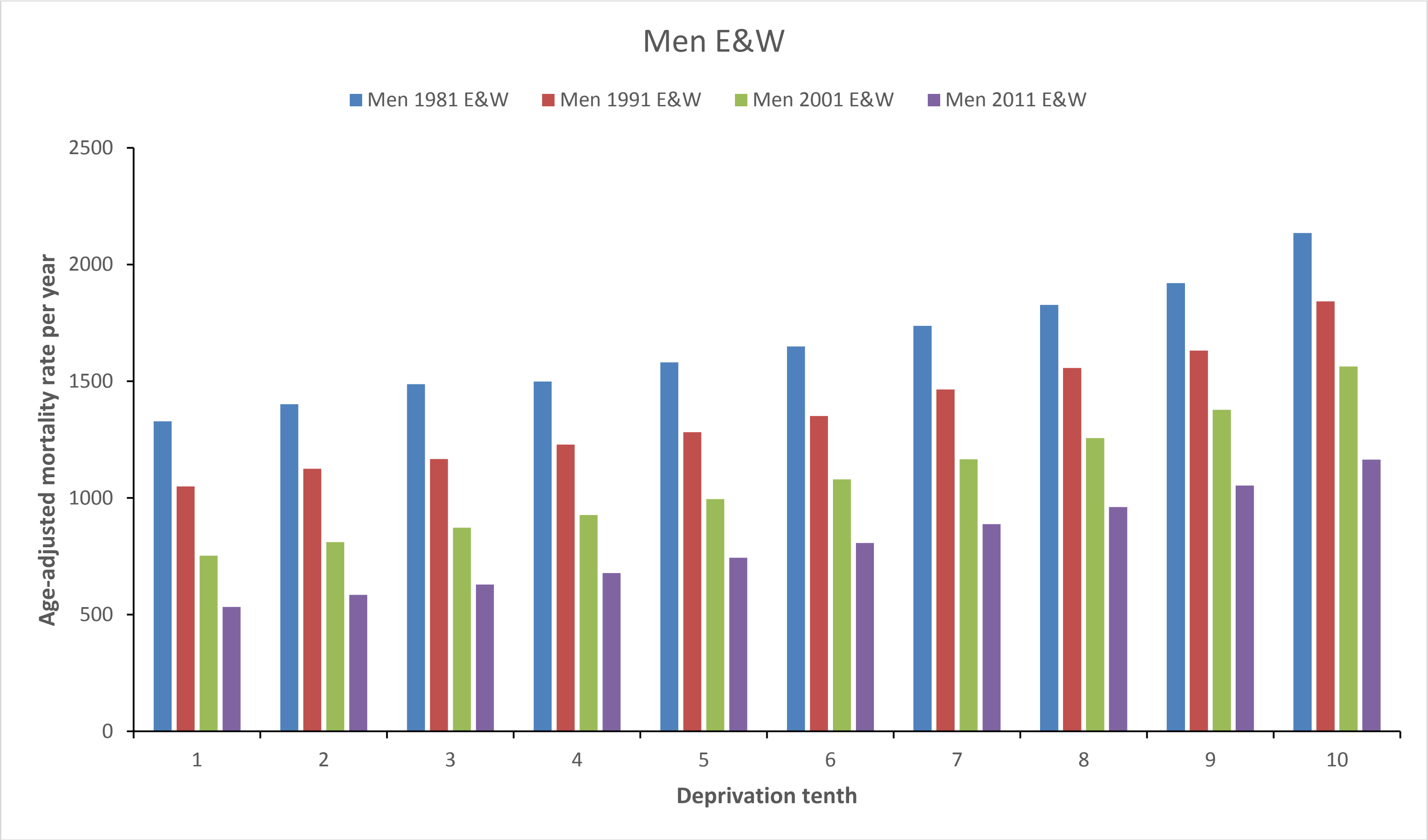
Men E&W
Men 1981 E&W
Men 1991 E&W
Men 2001 E&W
Men 2011 E&W
Age-adjusted mortality rate per year
2500
2000
1500
1000
500
0
1
2
3
4
5
6
7
8
9
10
Deprivation tenth

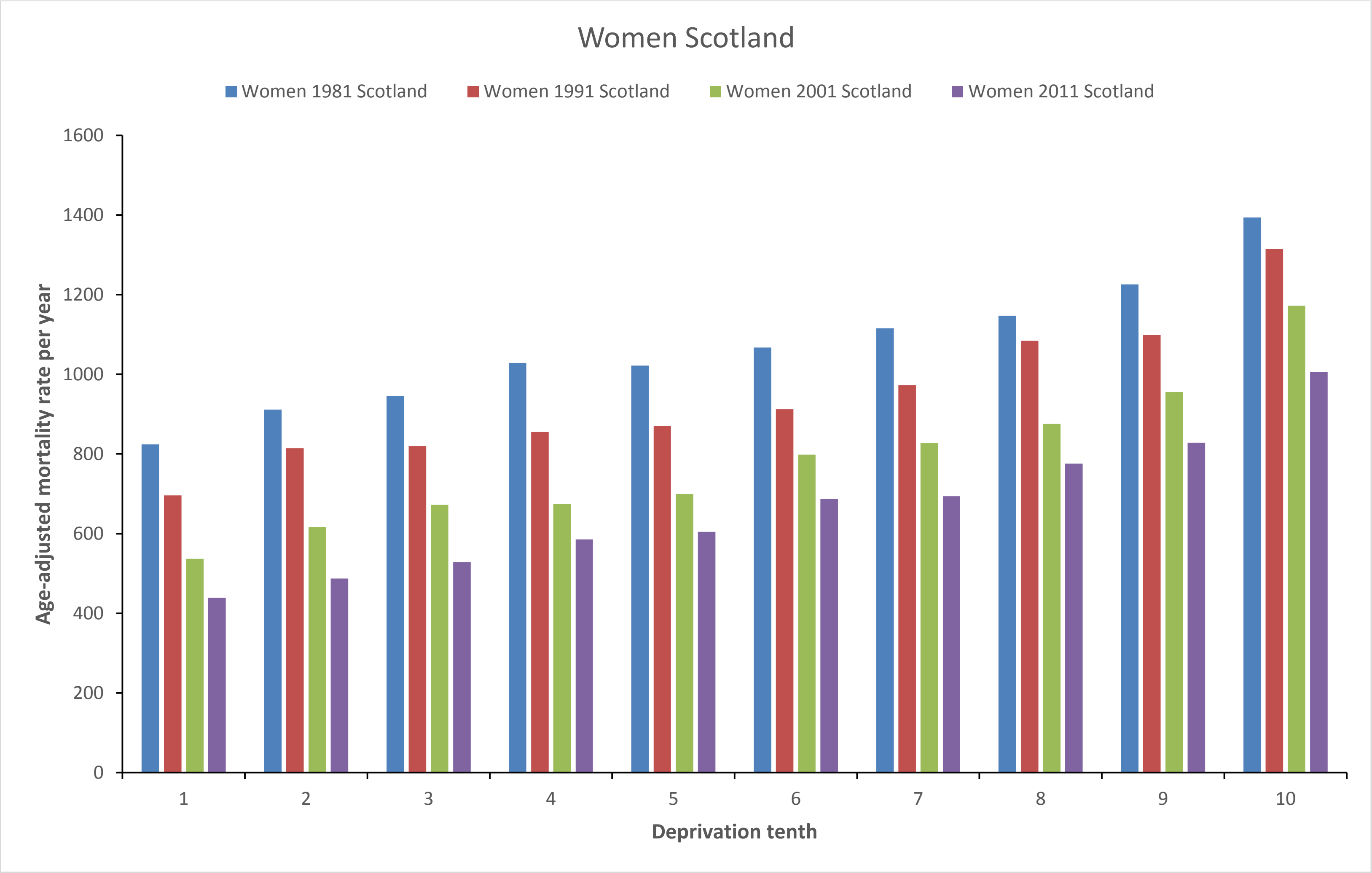
Women Scotland
Women 1981 Scotland
Women 1991 Scotland
Women 2001 Scotland
Women 2011 Scotland
Age-adjusted mortality rate per year
0
200
400
600
800
1000
1200
1400
1600
1
2
3
4
5
6
7
8
9
10
Deprivation tenth

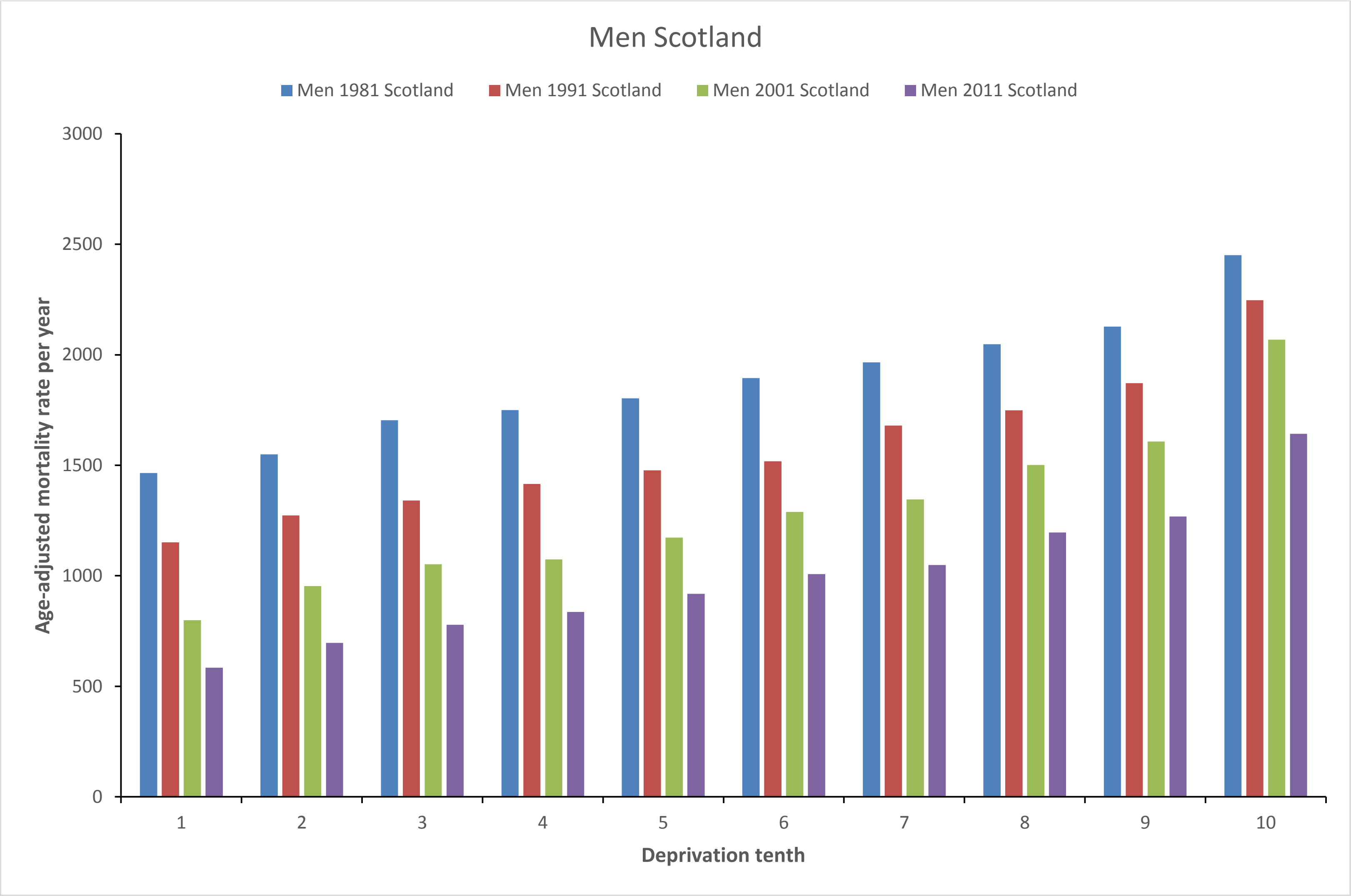
Men Scotland
Men 1981 Scotland
Men 1991 Scotland
Men 2001 Scotland
Men 2011 Scotland
Age-adjusted mortality rate per year
3000
2500
2000
1500
1000
500
0
1
2
3
4
5
6
7
8
9
10
Deprivation tenth

Relative Index of Inequality and Slope Index of Inequality

group	country	year	sex	SII	SII -lower ci	SI -upper CI	RII	RII -lower	RII _upper	SII per 100,000 per year		
E&W 1981 Men	E&W	1981	Men	0.007681	0.007525	0.007837645	1.6054	1.5913	1.619499	768.1188	752.5133	783.7645453
E&W 1991 Men	E&W	1991	Men	0.007477	0.007338	0.007616669	1.7599	1.7433	1.776685	747.7353	733.837	761.6668559
E&W 2001 Men	E&W	2001	Men	0.007581	0.007463	0.007699371	2.1199	2.098	2.142026	758.1331	746.35	769.9370795
E&W 2011 Men	E&W	2011	Men	0.006076	0.005981	0.006170969	2.2948	2.2686	2.321268	607.6128	598.1439	617.0969031
Scotland 1981 Men	Scotland	1981	Men	0.008873	0.008331	0.009418183	1.5937	1.5528	1.635678	887.267	833.1399	941.8182932
Scotland 1991 Men	Scotland	1991	Men	0.009623	0.00913	0.01011968	1.8457	1.7942	1.898683	962.3261	913.0035	1011.967998
Scotland 2001 Men	Scotland	2001	Men	0.010619	0.010193	0.011046661	2.3369	2.2673	2.408671	1061.894	1019.289	1104.666135
Scotland 2011 Men	Scotland	2011	Men	0.008786	0.008449	0.009123655	2.6362	2.5511	2.724258	878.5842	844.9002	912.3654795
E&W 1981 Women	E&W	1981	Women	0.003245	0.003145	0.003345687	1.4349	1.4202	1.449761	324.519	314.5077	334.5687
E&W 1991 Women	E&W	1991	Women	0.003561	0.003467	0.003656371	1.5807	1.5633	1.598297	356.1266	346.65	365.6371
E&W 2001 Women	E&W	2001	Women	0.003954	0.003868	0.004040811	1.8584	1.836	1.881023	395.4196	386.7829	404.0811
E&W 2011 Women	E&W	2011	Women	0.003622	0.003548	0.003695703	2.1035	2.0754	2.13204	362.1699	354.7871	369.5703
Scotland 1981 Women	Scotland	1981	Women	0.004713	0.004368	0.005062102	1.5436	1.4993	1.589246	471.3322	436.7923	506.2102
Scotland 1991 Women	Scotland	1991	Women	0.004971	0.00464	0.005305539	1.6877	1.6352	1.741814	497.1226	463.9918	530.5539
Scotland 2001 Women	Scotland	2001	Women	0.005301	0.005001	0.005602494	1.9778	1.9109	2.047052	530.0732	500.1122	560.2494
Scotland 2011 Women	Scotland	2011	Women	0.004999	0.004739	0.005259704	2.2371	2.1557	2.321455	499.8823	473.9428	525.9704

For peer review only

Interpolated 2008 for Scotland and 2007.5 for E&W figures		Linear interpolation	Log linear interpolation
E&W	Men	2.2336	2.231999
Scotland	Men	2.5464	2.542626
E&W	Women	2.0177	2.014243
Scotland	Women	2.1593	2.15591

Interpolated 1993 for Scotland and 1993.5 for E&W figures			
E&W	Men	1.8499	1.843737
Scotland	Men	1.944	1.934912
E&W	Women	1.6501	1.645948
Scotland	Women	1.7457	1.742076

For peer review only

Percentage change

% change over time periods		by Carstairs deprivation				By educational attainment	Mackenbach comparable figures are for 1991-5 to 2006-2010 for Scotland, and 1991-6 to 2006-9 for E&W
		1982-1991	1991-2001	1991-2011	1993-2008	1993-2008	Scotland midpoints: 1993 and 2008; E&W midpoints 1993.5 and 2007.5
**RII**							
E&W	Men	9.6%	1.4%		20.7%		20.7%
Scotland	Men	15.8%	10.3%		31.0%	-45.5%	31.0%
E&W	Women	10.2%			22.3%		22.3%
Scotland	Women	9.3%			23.7%	-20.0%	23.7%
**SII**							
E&W	Men	-2.7%			-12.0%		-12.0%
Scotland	Men	5.5%			-5.0%	-63.8%	-5.0%
E&W	Women	9.7%			2.1%		2.1%
Scotland	Women	5.5%			1.0%	-47.3%	1.0%

For peer review only

		Educational attainment			Carstairs deprivation		
		1991-1995	2006-2010	% change	1993	2008	% change
**RII**							
Scotland	Men	3.3	1.8	-45%	1.9	2.5	31%
Scotland	Women	2	1.6	-20%	1.7	2.2	24%
England & Wales	Men				1.8	2.5	38%
England & Wales	Women				1.6	2.0	23%
**Relative differences**							
Scotland	Men	1.81	1.83	1%			
Scotland	Women	1.57	1.75	11%			
England & Wales	Men	1.55	1.57	1%			
England & Wales	Women	1.46	1.46	0%			
**SII**							
Scotland	Men	1,634	591	-64%	982	934	-5%
Scotland	Women	601	317	-47%	504	509	1%
England & Wales	Men				750	660	-12%
England & Wales	Women				366	374	2%
**Absolute differences**							
Scotland	Men	681	502	-26%			
Scotland	Women	335	307	-8%			
England & Wales	Men	494	317	-36%			
England & Wales	Women	254	193	-24%			

For peer review only

For peer review only - http://bmjopen.bmj.com/site/about/guidelines.xhtml

STROBE 2007 (v4) Statement—Checklist of items that should be included in reports of *cross-sectional studies*

Section/Topic	Item #	Recommendation	Reported on page #
**Title and abstract**	1	(*a*) Indicate the study's design with a commonly used term in the title or the abstract	1
		(*b*) Provide in the abstract an informative and balanced summary of what was done and what was found	1
**Introduction**			
Background/rationale	2	Explain the scientific background and rationale for the investigation being reported	2-3
Objectives	3	State specific objectives, including any prespecified hypotheses	3
**Methods**			
Study design	4	Present key elements of study design early in the paper	4
Setting	5	Describe the setting, locations, and relevant dates, including periods of recruitment, exposure, follow-up, and data collection	4
Participants	6	(*a*) Give the eligibility criteria, and the sources and methods of selection of participants	4
Variables	7	Clearly define all outcomes, exposures, predictors, potential confounders, and effect modifiers. Give diagnostic criteria, if applicable	4
Data sources/ measurement	8*	For each variable of interest, give sources of data and details of methods of assessment (measurement). Describe comparability of assessment methods if there is more than one group	4
Bias	9	Describe any efforts to address potential sources of bias	4-5
Study size	10	Explain how the study size was arrived at	4
Quantitative variables	11	Explain how quantitative variables were handled in the analyses. If applicable, describe which groupings were chosen and why	4
Statistical methods	12	(*a*) Describe all statistical methods, including those used to control for confounding	4-5
		(*b*) Describe any methods used to examine subgroups and interactions	4-5
		(*c*) Explain how missing data were addressed	4-5
		(*d*) If applicable, describe analytical methods taking account of sampling strategy	n/a
		(*e*) Describe any sensitivity analyses	n/a
**Results**			

For peer review only

Participants	13*	(a) Report numbers of individuals at each stage of study—eg numbers potentially eligible, examined for eligibility, confirmed eligible, included in the study, completing follow-up, and analysed	Supplement
		(b) Give reasons for non-participation at each stage	n/a
		(c) Consider use of a flow diagram	n/a
Descriptive data	14*	(a) Give characteristics of study participants (eg demographic, clinical, social) and information on exposures and potential confounders	Supplement
		(b) Indicate number of participants with missing data for each variable of interest	n/a
Outcome data	15*	Report numbers of outcome events or summary measures	Supplement
Main results	16	(*a*) Give unadjusted estimates and, if applicable, confounder-adjusted estimates and their precision (eg, 95% confidence interval). Make clear which confounders were adjusted for and why they were included	5, 6 and supplement
		(*b*) Report category boundaries when continuous variables were categorized	n/a
		(*c*) If relevant, consider translating estimates of relative risk into absolute risk for a meaningful time period	n/a
Other analyses	17	Report other analyses done—eg analyses of subgroups and interactions, and sensitivity analyses	n/a
**Discussion**			
Key results	18	Summarise key results with reference to study objectives	7
Limitations	19	Discuss limitations of the study, taking into account sources of potential bias or imprecision. Discuss both direction and magnitude of any potential bias	8
Interpretation	20	Give a cautious overall interpretation of results considering objectives, limitations, multiplicity of analyses, results from similar studies, and other relevant evidence	8-9
Generalisability	21	Discuss the generalisability (external validity) of the study results	8-9
**Other information**			
Funding	22	Give the source of funding and the role of the funders for the present study and, if applicable, for the original study on which the present article is based	9

*Give information separately for cases and controls in case-control studies and, if applicable, for exposed and unexposed groups in cohort and cross-sectional studies.

Note: An Explanation and Elaboration article discusses each checklist item and gives methodological background and published examples of transparent reporting. The STROBE checklist is best used in conjunction with this article (freely available on the Web sites of PLoS Medicine at http://www.plosmedicine.org/, Annals of Internal Medicine at http://www.annals.org/, and Epidemiology at http://www.epidem.com/). Information on the STROBE Initiative is available at www.strobe-statement.org.

For peer review only
